# Quantifying treatment response to a macrophage-targeted therapy in combination with immune checkpoint inhibitors after exposure to conventional chemotherapy

**DOI:** 10.3389/fimmu.2025.1565953

**Published:** 2025-04-28

**Authors:** Shelby N. Bess, Gaven K. Smart, Timothy J. Muldoon

**Affiliations:** Department of Biomedical Engineering, University of Arkansas, Fayetteville, AR, United States

**Keywords:** macrophages, metabolism, cancer, immune checkpoint inhibitor, chemotherapy, spheroids

## Abstract

**Background:**

Conventional chemotherapeutic agents, such as 5-fluorouracil (5-FU), can exert anti-tumor effects through immunogenic cell death (ICD) induction. Researchers have found hallmarks that quantify ICD (such as the translocation of HMGB1 and calreticulin). Although chemotherapeutic agents can induce ICD, they increase the expression of immune checkpoints, limiting their effectiveness. Studies have emphasized the importance of investigating the heterogeneous responses of cells co-localized in a solid tumor (macrophages, tumor cells, etc.) to ICD induction. However, these studies were performed *in vivo*, which limits the collection of information on cell-cell interactions due to model complexity.

**Methods:**

In this study, we used a multicellular spheroid model in conjunction with single spheroid imaging to understand the structural and metabolic changes of a simulated solid tumor model. In addition to using the spheroid model, conventional 2D co-culture monolayers were used to quantify ICD hallmarks and changes in macrophage functional behavior while correlating immune responses after exposure to the combinatory regimen of immune checkpoint inhibitors and an ICD inducer.

**Results:**

Results indicate that the combination of two immune checkpoint inhibitors in addition to a chemotherapy agent reduced spheroid growth (~46%) and reduced M2 macrophage expression and cellular proliferation while modulating cellular metabolism, ICD hallmarks, and phagocytic function.

**Conclusions:**

Overall, this study not only quantified microregional metabolic and structural changes in a simulated spheroid model but also quantified changes in ICD hallmarks and macrophage functional behavior. It was also found that correlations between spheroid structure and ICD hallmarks through immunofluorescence markers could exist after exposure to the combinatory regimen of immune checkpoint inhibitors and an ICD inducer.

## 1 Introduction

Cancer is the second-leading cause of death in the United States, accounting for an estimated 2,000,000 new cases and 600,000 new deaths in 2024 (1). Colorectal cancer (CRC), specifically, is expected to have an estimated 153,000 new cases and 53,000 new deaths in 2024 (1). Patients with locally advanced CRC (stage II and stage III) and metastatic CRC were traditionally treated with surgery followed by adjuvant and neoadjuvant chemotherapy regimens such as FOLFOX (a combination of 5-fluoruracil (5-FU), leucovorin, and oxaliplatin) (2). Even though it is considered the gold standard of CRC treatment, systemic side effects (such as nausea, fatigue, decrease in white blood cells, etc.), low survival rates (~10%), and high recurrence rates (~30-40%) still occur and are still a major concern for clinicians (3). Therefore, researchers and clinicians are exploring new therapeutic interventions to overcome these limitations, with immunotherapy becoming a popular target.

Immunotherapy is used to treat cancer by enhancing or stimulating the immune system or components of the immune system (T-cells, macrophages, dendritic cells (DCs), etc.) to target and inhibit the proliferation of cancer cells while limiting negative systemic effects associated with untargeted chemotherapy approaches (4). Several clinically approved approaches, such as cancer vaccines, adoptive cell transfer (ACT) therapies, and monoclonal antibody therapies, more specifically immune checkpoint inhibitors, have gained clinical traction for treating CRC in recent years (5). Immune checkpoint inhibitors are ligand-mediated inhibitory pathways that help the immune system maintain homeostasis through the regulation of the duration and amplitude of immune responses (6, 7). Several immune checkpoints have been used to treat numerous cancer types, such as melanoma, bladder, non-small cell lung cancer (NSCLC), and CRC, with the most popular being cytotoxic T-lymphocyte antigen-4 (CTLA-4) and programmed cell death ligand-1 (PD-L1) (8).

PD-L1 is a well-studied immune checkpoint with the primary function of suppressing immune responses to regulate autoimmunity and tolerance (9, 10). PD-L1 is commonly expressed on T-cells, B-cells, macrophages, cancer cells, and DCs and is upregulated through the release of pro-tumor cytokines (such as interleukin-4 (IL-4), IL-10, and vascular endothelial growth factor (VEGF)) from cancer cells and is associated with poor prognosis (11, 12). The binding of PD-1/PD-L1 results in T-cell apoptosis, which is problematic in solid tumors as some T-cells to survive apoptosis to become memory T-cells (13). This makes targeting the PD-L1/PD-1 axis an active target for CRC immunotherapy to improve anti-tumor immune response. However, the targeting of PD-L1/PD-1 cannot be used to treat all cancer types, such as pancreatic, prostate, and gastric cancers (14, 15).

Recent studies have emphasized the importance of the tumor microenvironment’s heterogeneous cell population and its response to immune checkpoint inhibitors (16). In CRC, patients typically have a poor response to immune checkpoint inhibitors due to their low immunogenicity and low tumor-infiltrated CD8+ T-cells, leading to metastatic growth and a decrease in overall survival (17, 18). Researchers have found that one way to enhance a tumor’s poor immunogenicity is to utilize immunologic cell death (ICD) (19, 20). ICD is a type of cancer cell death that is triggered via certain chemotherapeutic drugs, radiotherapy, and physicochemical therapies through the activation of the immune system against cancer in immunocompetent hosts (21, 22). ICD comprises the release of damage-associated molecular patterns (DAMPs) from apoptotic cancer cells that lead to the activation of tumor-specific immune responses (the induction of mitochondrial reactive oxygen species and cell stress), eliciting long-term efficacy of antitumor drugs through the combination of antitumor immunity and direct cancer cell killing (23). More specifically, the two hallmarks associated with immunogenic cell death, the secretion of adenosine triphosphate (ATP) from dying tumor cells and the nuclear to cytoplasmic translocation of high mobility group B1 (HMGB1), are the gold standards for accurately predicting the ICD-inducing capacity of therapeutic regimens such as chemotherapeutic agents. The release of ATP during the zeiosis (blebbing) phase of apoptosis has been regarded as a find me signal, as it can constitute a chemoattractant for dendritic cell precursors, leading to the adaptive immune response and changes in the glycolytic metabolism pathway to cancer cells mediated by IFN-*y*-producing CD8+ T cells. During the late stages of apoptosis, HMGB1 is released from the nucleus of damaged cells, which is vital for activating dendritic cells and facilitating antigen presentation to T cells. FOLFOX and 5-FU have been shown to induce ICD effectively; however, they increase the expression of immune checkpoints [such as PD-L1 and cluster of differentiation 47 (CD47)] in tumor cells and surrounding immune cells through NF-KB signaling induced by chemotherapy, leading to potential chemotherapy resistance (23).

CD47 is a widely expressed transmembrane protein with an array of cellular functions and multiple binding partners (24). CD47 is a don’t eat me signal that inhibits phagocytosis through binding with signal-regulating protein alpha (SIRPα) on the surface of phagocytic cells, such as macrophages (25, 26). The upregulation of CD47 not only increases a cancer cell’s selfness but also leads to the blocking of cross-presentation by antigen-presenting cells (APCs) (27, 28). The blockade of CD47/SIRPα is an emerging target in cancer immunotherapy (29). Published phase I clinical trials have shown some clinical benefits of targeting this immune checkpoint; however, monotherapies blocking CD47/SIRPα fail to act as a curative treatment (30, 31). Because CD47 is widely expressed on normal and tumor cells, substantial doses or frequent dosages may be necessary to achieve effective therapeutic CD47 blockage (~40-60% CD47 receptor occupancy for induction of phagocytosis).

A recent study has shown that the *in vivo* blockade of CD47/SIRPα, in addition to an ICD inducer, resulted in increased survival and a reduction in tumor size (14). However, studying the cell-cell interactions between macrophages and cancer cells in an *in vivo* model is difficult due to the high model complexity and lack of control of variables (38). Three-dimensional *in vitro* culture methods represent an excellent alternative to traditional *in vivo* models, as they allow for better control of variables (oxygen and nutrient gradients, pH, temperature, etc.), enhanced reproducibility, and help facilitate the study of cellular and molecular mechanisms (32, 33). In this study, we aim to use a multicellular spheroid model in conjunction with single spheroid imaging to understand the microregional structural and metabolic changes of a simulated solid tumor model, in addition to using conventional 2D co-culture monolayers to not only quantify characteristic features of ICD (HMGB1 and ATP) and changes in macrophage functional behavior but also help validate and correlate immune responses after exposure to the combinatory regimen of immune checkpoint inhibitors and an ICD inducer.

## 2 Methods

### 2.1 Cancer cell and macrophage culture

For all 3D multicellular spheroid culture experiments, the methods used for culturing of murine RAW 264.7 (ATCC^©^, TIB-71) and CT26 colorectal adenocarcinoma (ATCC^©^, CRL-2638) cancer cells before all experiments were performed as described in Bess et al. (34). For all 2D co-culture experiments, the methods used for culturing of murine RAW 264.7 (ATCC^©^, TIB-71) and CT26 colorectal adenocarcinoma (ATCC^©^, CRL-2638) cancer cells before all experiments were performed as described in Bess et al. (34). For this study, 2D co-cultures were modeled to mimic the proliferative edge of the 3D multicellular spheroid model described below.

### 2.2 3D multicellular spheroid culture

Methodologies from Bess et al. (34) were used to create the 3D multicellular spheroids. Briefly, RAW 264.7 macrophages and CT26 cells were brought to specific concentrations before the creation of a single-cell suspension. 20 μL hanging drops (n = 50 ± 5) of the cell suspension were placed on an inverted petri dish lid and placed over a petri dish containing PBS. Dishes were incubated in a 37°C incubator at 5% CO_2_ on an orbital shaker at 70 RPM. After 3 days, the hanging drops were washed from the lid with RPMI media, centrifuged, and transferred to 6-well plates. Spheroids developed further for seven additional days under the same conditions with media changes occurring every two days through careful collection and transfer procedures.

### 2.3 Therapeutic treatments and schedules

**Figure 1:**
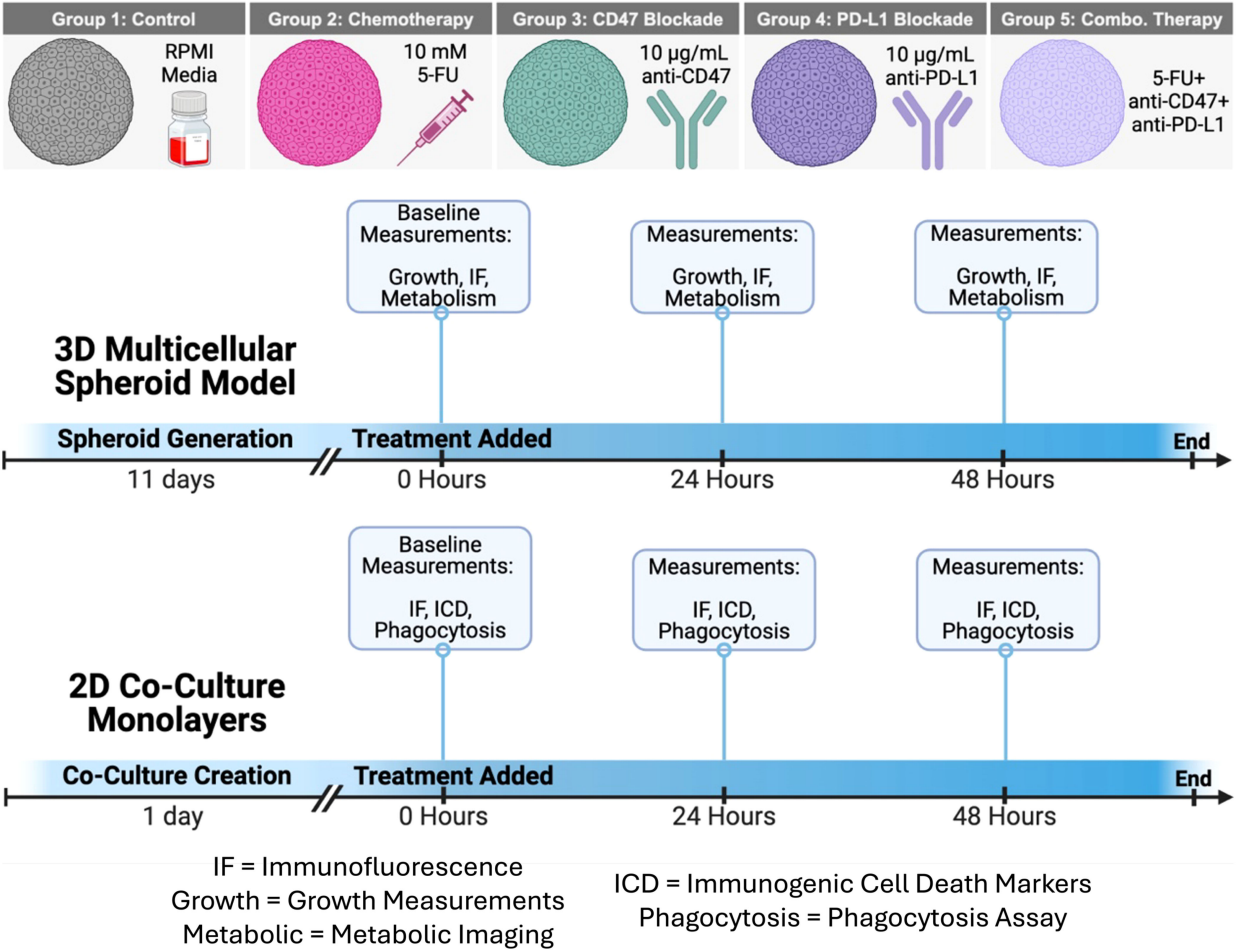
Schematic of therapeutic treatments and treatment schedule for 3D multicellular spheroids and 2D co-culture monolayers. Growth, Growth Curves; IF, Immunofluorescence Staining; Metabolism, Metabolic Imaging; ICD, Immunogenic Cell Death Hallmarks; Phagocytosis, Phagocytosis Assay. Figure created in BioRender.

For 2D and 3D cultures, the control group received no treatment and received only RPMI culture medium. For the chemotherapy group, 5-fluorouracil (5-FU) powder (Sigma Aldrich, #F6627-10G) was diluted in DMSO at a concentration of 40 mg/mL. A second dilution was created in RPMI culture medium to bring the 5-FU concentration to 10 mM. For the two immunotherapy groups, anti-CD47 (BioXCell, #BE0270) and anti-PD-L1 (BioXCell, #BE0361) were shipped at 9.89 mg/mL and 8.06 mg/mL, respectively, and stored at 4°C before dilution. On the day of treatment, anti-PD-L1 and anti-CD47 were diluted with RPMI culture medium to a concentration of 10 µg/mL. The combination treatment group received a cocktail of 5-FU, anti-CD47, and anti-PD-L1. At the 0-hour timepoint, no treatments were given to model baseline measurements. After this initial time point, a single therapeutic dose was added to the spheroids or 2D co-cultures and allowed to incubate for 24 and 48 hours (**Figure 1**). All cultures were maintained under identical conditions throughout the study.

### 2.4 Immunofluorescence staining and imaging

Immunofluorescence staining was performed on 2D co-cultures to quantify immunogenic cell death hallmarks (HMGB1 and calreticulin) and validate changes in macrophage (CD80/CD206, SIRP-α, and PD-1) and cancer cell (CD47 and PD-L1) markers, while additional immunofluorescence staining was performed to characterize individual cell populations and the structural micro-regions within the multicellular spheroid model. **Table 1** summarizes the immunofluorescence antibodies used.

**Table 1:** Immunofluorescence antibodies used to characterize 2D co-culture monolayers and 3D spheroid cultures.

*Target*	Marker	Conjugate	Vendor and Item Number	Culture System
*M1 Macrophages*	CD80	Brilliant Violet™ 421	Biolegend^®^, 104726	2D, 3D
*M2 Macrophages*	CD206	AlexaFluor™ 594	Biolegend^®^, 141726	2D, 3D
*All Macrophages*	CD68	AlexaFluor™ 488	Biolegend^®^, 137012	2D, 3D
*PD-L1*	PD-L1	AlexaFluor™ 488	Novus Biologics^®^, NBP1767AF488	2D, 3D
*PD-1*	PD-1	AlexaFluor™ 594	Novus Biologics^®^, NBP175518AF594	2D, 3D
*CD47*	CD47	AlexaFluor™ 405	Novus Biologics^®^, NBP231106AF405	2D, 3D
*SIRP-α*	SIRP-α	AlexaFluor™ 594	Novus Biologics^®^, NBP177045AF594	2D, 3D
*HMGB1*	HMGB1	Dylight™ 488	ThermoFisher^®^, PA522721	2D
*Calreticulin*	Calreticulin	Dylight™ 594	Novus Biologics^®^, FAB38981M	2D
*Proliferation*	Ki67	AlexaFluor™ 488	Novus Biologics^®^, NB500170AF488	3D
*Apoptosis*	CC3	AlexaFluor™ 594	Novus Biologics^®^, NB10056708AF594	3D
*Acute Hypoxia*	HIF-1α	FITC	ThermoFisher^®^, MA545251	3D
*Chronic Hypoxia*	HIF-2α	DyLight™ 650	ThermoFisher^®^, PA522694	3D

To perform staining on 2D co-cultures, co-cultures were washed with PBS for 1 minute. 10% neutral buffered formalin was added to the spheroids and allowed to incubate at room temperature for 10 minutes. Co-cultures were then washed three times with PBS for 1 minute each. 0.2% Triton-X100 was added to the co-cultures and allowed to incubate at room temperature for 15 minutes. Co-cultures were then washed three times with PBS for 1 minute each. Before antibody addition, 2% bovine serum albumin (BSA) was added and allowed to incubate for 60 minutes at room temperature. Primary antibodies were added and allowed to incubate overnight at 4°C. Co-cultures were washed three times with PBS for 1 minute each before counterstaining with DAPI for 5 minutes, where applicable. Images were acquired using an inverted laser scanning confocal microscope (Olympus Fluoview FV10i-LiV) with a 60X (1.2 N.A., water immersion) objective.

For 3D spheroids, all staining was performed in suspension within a microcentrifuge tube using the methodologies described in Bess et al. (34). Spheroids were then washed with PBS, then added to a glass slide, and mounted with Fluoromount G and a coverslip. Images were acquired with a wide-field upright microscope (Nikon, Eclipse Ci) with a 10X/0.3NA objective lens (Nikon, CFI Plan Fluor 10X), a digital camera (Nikon, DS-Fi2), and a PC-based camera control unit (Nikon, DS-U3). Experiments were performed in triplicate with n = 40 spheroids imaged.

### 2.5 Phagocytosis assay

The methods used to perform analysis of macrophage phagocytosis function in 2D co-cultures were performed as described in Bess et al. (35). Briefly, CT26 cells were harvested and stained with 120 ng/mL of pHrodo-SE (Invitrogen^TM^, P36600), while RAW 264.7 macrophages were harvested and stained with 1 μM 5-chloromethylfluorescein diacetate (CMFDA, Invitrogen^TM^ CellTracker^TM^, C2925) for 30 minutes at room temperature. After PBS washing for both cell types, CT26 and RAW 264.7 cells were co-incubated together for 30 minutes at 37°C and 5% CO_2_. After incubation, dishes were moved to a confocal microscope (Olympus Fluoview FV10i-LiV) with a 60X (1.2 N.A., water-immersion) objective with controllable temperature and humidified gas delivery (5% CO_2_). The degree of phagocytosis was analyzed by randomly selecting six microscopic fields of view.

### 2.6 3D growth curve measurements

Growth curves were used to assess spheroid growth before and after treatment (34). Spheroids were imaged with a wide-field upright microscope (Nikon, Eclipse Ci) using a 4X/0.13NA objective lens (Nikon, CFI Plan Fluor 10X), digital camera (Nikon, DS-Fi2), and PC-based camera control unit (Nikon, DS-U3). The diameter of each spheroid within each FOV was measured using ImageJ software. Experiments were performed in triplicate, with n = 50 spheroids at each time point.

### 2.7 Live-spheroid metabolic imaging and processing of spheroid immunofluorescence and multiphoton images

Methodologies previously described in Bess et al. (34) were used to capture NAD(P)H and FAD autofluorescence images along with fluorescence lifetime images (FLIM) using multiphoton microscopy. Data analysis methodologies from Bess et al. (34) were also used to pre-process spheroid images and capture intensity distributions of immunofluorescence markers and NADH and FAD autofluorescence images of multicellular spheroids using a novel radial line profiling MATLAB script.

### 2.8 Statistics

An ordinary two-way analysis of variance (ANOVA) and Tukey’s multiple comparisons test were used to determine the statistical significance. A *p*-value of < 0.05 is considered statistically significant.

## 3 Results

### 3.1 Combination therapy shows decrease in spheroid growth

**Figure 2:**
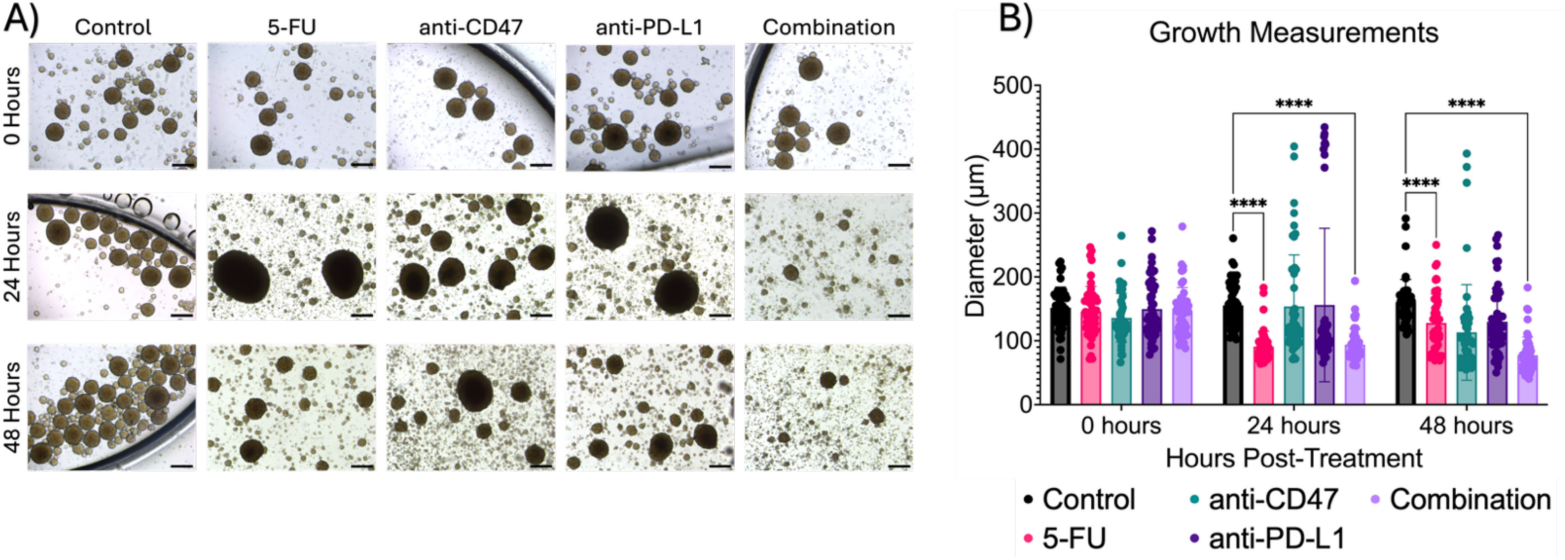
Combination regimen shows significant changes in spheroid diameter (µm) over time. **(A)** Representative brightfield images of spheroids before and after treatment. **(B)** Growth measurements for treatment groups before and after treatment. ****p < 0.0001. Plots were made in GraphPad Prism ^®^. Scale bars are 20 µm.

To study the growth effects of the combination therapy, spheroid diameters were measured (**Figure 2**). The control group showed a consistent diameter range over time (~150 to 160 µm). After 24 hours, the 5-FU group (90.832 ± 24.008 µm) and the combination group (93.937 ± 24.751 µm) showed a significant decrease in diameter when compared to the control (p < 0.0001). Similar trends were also observed after 48 hours (p < 0.0001). Additional statistical comparisons were made within each group (**Supplementary Figure 1**) to investigate time effects. Statistical differences were observed 24 (p < 0.0001) and 48 (p = 0.0019) hours post-treatment for 5-FU-treated spheroids. The anti-CD47 and anti-PD-L1 groups also showed statistical differences after 48 hours (p = 0.0006 and p = 0.0397, respectively). Additional statistical differences were observed within the combination group across all time points (p < 0.0001).

Lastly, we investigated whether the therapeutic regimens showed changes in the distribution of spheroid diameters (**Supplementary Figure 2**). Within the control group, ~50% of spheroids fell between 140 and 160 µm across all time points. For 5-FU-treated spheroids, ~40% of spheroids fell between 120 and 140 µm before treatment with a shift of ~80% of spheroids falling in the diameter range to 80 and 100 µm after 24 hours, with similar trends holding after 48 hours. For anti-CD47-treated spheroids, ~50% of spheroids fall in the diameter range of 120 and 140 µm before treatment, with a shift of ~50% of spheroids shifting to a diameter range of 100 and 120 µm after 24 hours. After 48 hours, another shift occurs with 70% of spheroids falling in the diameter range of 60 and 100 µm. Interestingly, for anti-PD-L1-treated spheroids, ~30 to 60% of spheroids fall within the diameter range of 100 and 120 µm across all time points. In the combination group, before treatment, 68% of spheroids fall within the diameter range of 120 and 160 µm. 24 hours post-treatment, 76% of spheroids shift to a diameter range of 80 and 100 µm with another shift that occurs with 75% of spheroids falling in the diameter range of 60 and 80 µm after 48 hours. Overall, results indicate the combination therapy slows spheroid growth over time.

### 3.2 Treatments show significant changes in macrophage populations in spheroid microregions

**Figure 3:**
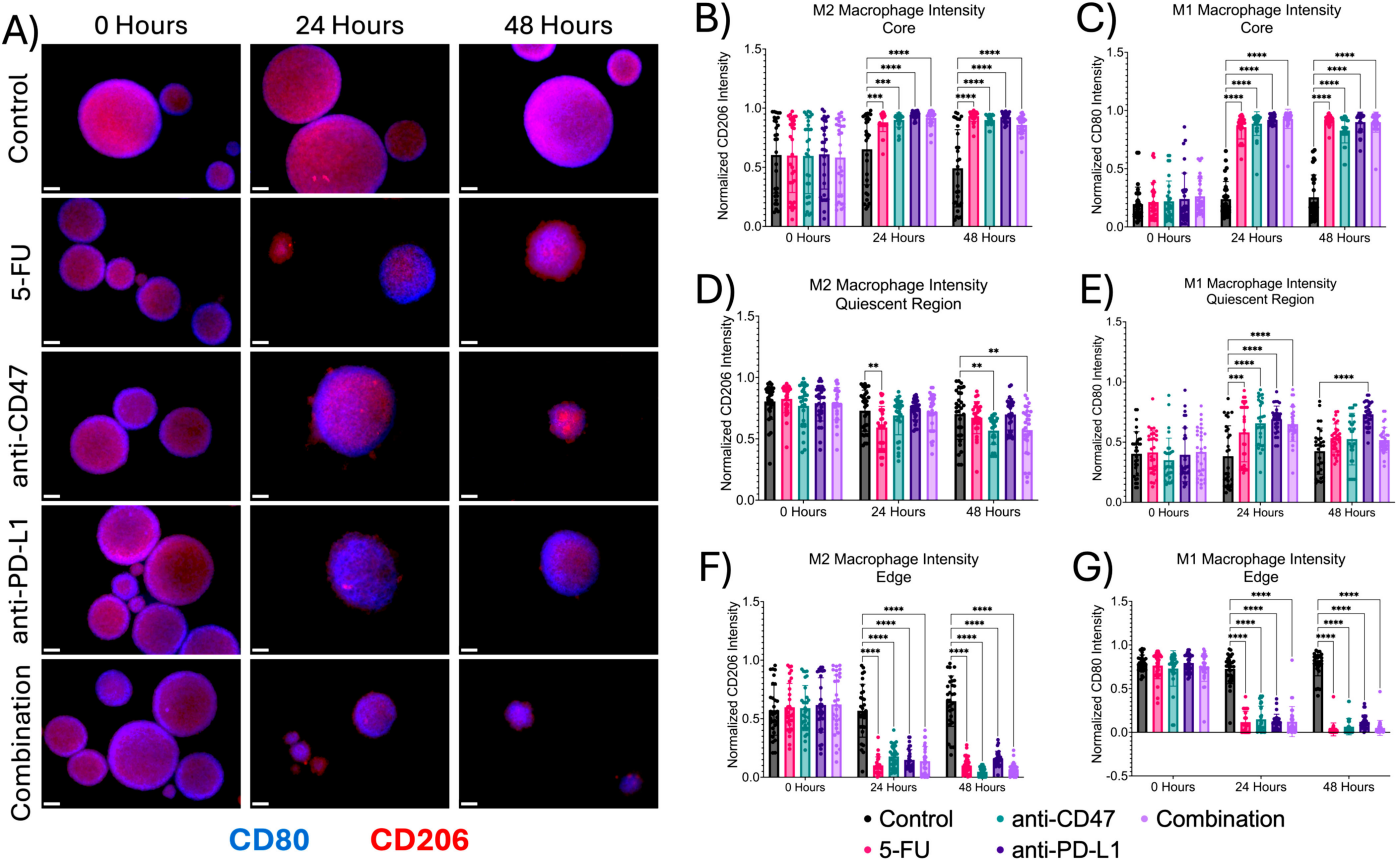
Macrophage expression across spheroid regions show significant changes before and after treatment **(A)** Representative immunofluorescence images of M1 macrophage (CD80) and M2 macrophage populations (CD206) across spheroid regions before and after treatment. **(B–G)** Normalized CD80 and CD206 pixel intensities. **p ≤ 0.01,***p ≤ 0.001, ****p ≤ 0.0001. Plots were made in GraphPad Prism ®. Scale bars are 20 µm.

To study the effects of how treatment affects the cellular expression of macrophages within the multicellular spheroids across microregions, immunofluorescence was performed (**Figure 3**). At the core, regardless of treatment, there were significant increases in all macrophage populations after treatment. For M2 populations, 5-FU-treated spheroids showed a normalized pixel intensity of 0.880 ± 0.078 (p = 0.0005) after 24 hours and 0.933 ± 0.045 (p < 0.0001) after 48 hours. Anti-CD47 treated spheroids showed a normalized pixel intensity of 0.900 ± 0.060 (p = 0.0001) after 24 hours and 0.900 ± 0.041 (p < 0.0001) after 48 hours. Anti-PD-L1 treated spheroids showed a normalized pixel intensity of 0.943 ± 0.028 (p < 0.0001) after 24 hours and 0.924 ± 0.039 (p < 0.0001) after 48 hours. In the combination group, the normalized pixel intensity was 0.918 ± 0.067 (p < 0.0001) after 24 hours and 0.859 ± 0.075 (p < 0.0001) after 48 hours. For M1 populations, 5-FU-treated spheroids showed normalized pixel values of 0.857 ± 0.102–24 hours post-treatment (p < 0.0001) and 0.916 ± 0.041–48 hours post-treatment (p < 0.0001). Anti-CD47 treated spheroids showed normalized CD80 pixel intensity values of 0.887 ± 0.102–24 hours post-treatment (p < 0.0001) and 0.828 ± 0.107–48 hours post-treatment (p < 0.0001). Anti-PD-L1 treated spheroids showed normalized pixel intensity values of 0.920 ± 0.038–24 hours post-treatment (p < 0.0001) and 0.901 ± 0.085–48 hours post-treatment (p < 0.0001). The combination group showed normalized pixel values of 0.929 ± 0.0082–24 hours post-treatment (p < 0.0001) and 0.900 ± 0.086–48 hours post-treatment (p < 0.0001).

In the quiescent region, there was a decrease in M2 macrophages and an increase in M1 macrophages. For M2 macrophage populations, for 5-FU-treated spheroids, normalized CD206 pixel intensity was 0.593 ± 0.171–24 hours post-treatment (p = 0.0042). For anti-CD47-treated spheroids, normalized CD206 pixel intensity was 0.567 ± 0.113 for 48 hours post-treatment (p = 0.0050). In the combination group, normalized CD206 pixel intensity was 0.572 ± 0.192–48 hours post-treatment (p = 0.0077). For M1 macrophage populations, 5-FU-treated spheroids showed normalized pixel values of 0.581 ± 0.245–24 hours post-treatment (p = 0.0003). Anti-CD47-treated spheroids showed normalized values of 0.658 ± 0.185–24 hours post-treatment (p < 0.0001). Anti-PD-L1-treated spheroids showed normalized values of 0.691 ± 0.109–24 hours post-treatment (p < 0.0001) and 0.732 ± 0.100–48 hours post-treatment (p < 0.0001). In the combination group, normalized pixel values were 0.651 ± 0.144–24 hours post-treatment (p < 0.0001).

At the edge, the addition of treatments decreased M1 and M2 macrophage populations. For M2 macrophage populations, 5-FU-treated spheroids showed a normalized pixel intensity value of 0.102 ± 0.072–24 hours post-treatment (p < 0.0001) and 0.100 ± 0.072–48 hours post-treatment (p < 0.0001). Anti-CD47-treated spheroids showed a normalized pixel intensity value of 0.178 ± 0.096–24 hours post-treatment (p < 0.0001) and 0.047 ± 0.037–48 hours post-treatment (p < 0.0001). Anti-PD-L1-treated spheroids showed a normalized pixel intensity value of 0.149 ± 0.087–24 hours post-treatment (p < 0.0001) and 0.160 ± 0.062–48 hours post-treatment (p < 0.0001). The combination group showed a normalized pixel intensity of 0.138 ± 0.125–24 hours post-treatment (p < 0.0001) and 0.068 ± 0.053–48 hours post-treatment (p < 0.0001). For M1 macrophage populations, 5-FU-treated spheroids showed normalized pixel values of 0.117 ± 0.123–24 hours post-treatment (p < 0.0001) and 0.033 ± 0.074–48 hours after treatment (p < 0.0001). Anti-CD47-treated spheroids showed normalized values of 0.149 ± 0.150–24 hours post-treatment (p < 0.0001) and 0.065 ± 0.094–48 hours post-treatment (p < 0.0001). Anti-PD-L1-treated spheroids showed normalized values of 0.125 ± 0.082–24 hours post-treatment (p < 0.0001) and 0.120 ± 0.079–48 hours post-treatment (p < 0.0001). In the combination group, normalized pixel values were 0.122 ± 0.173–24 hours post-treatment (p < 0.0001) and 0.050 ± 0.086–48 hours post-treatment (p < 0.0001). In addition to comparing CD206 and CD80 expression to the control, statistical comparisons were made within each group (**Supplementary Figure 3** and **Supplementary Table 1**). Overall, results indicate that the addition of immune checkpoint inhibitors to an ICD inducer decreased macrophage expression in spheroids across all microregions except the core.

### 3.3 Treatments show significant changes in spheroid structure across spheroid microregions

**Figure 4:**
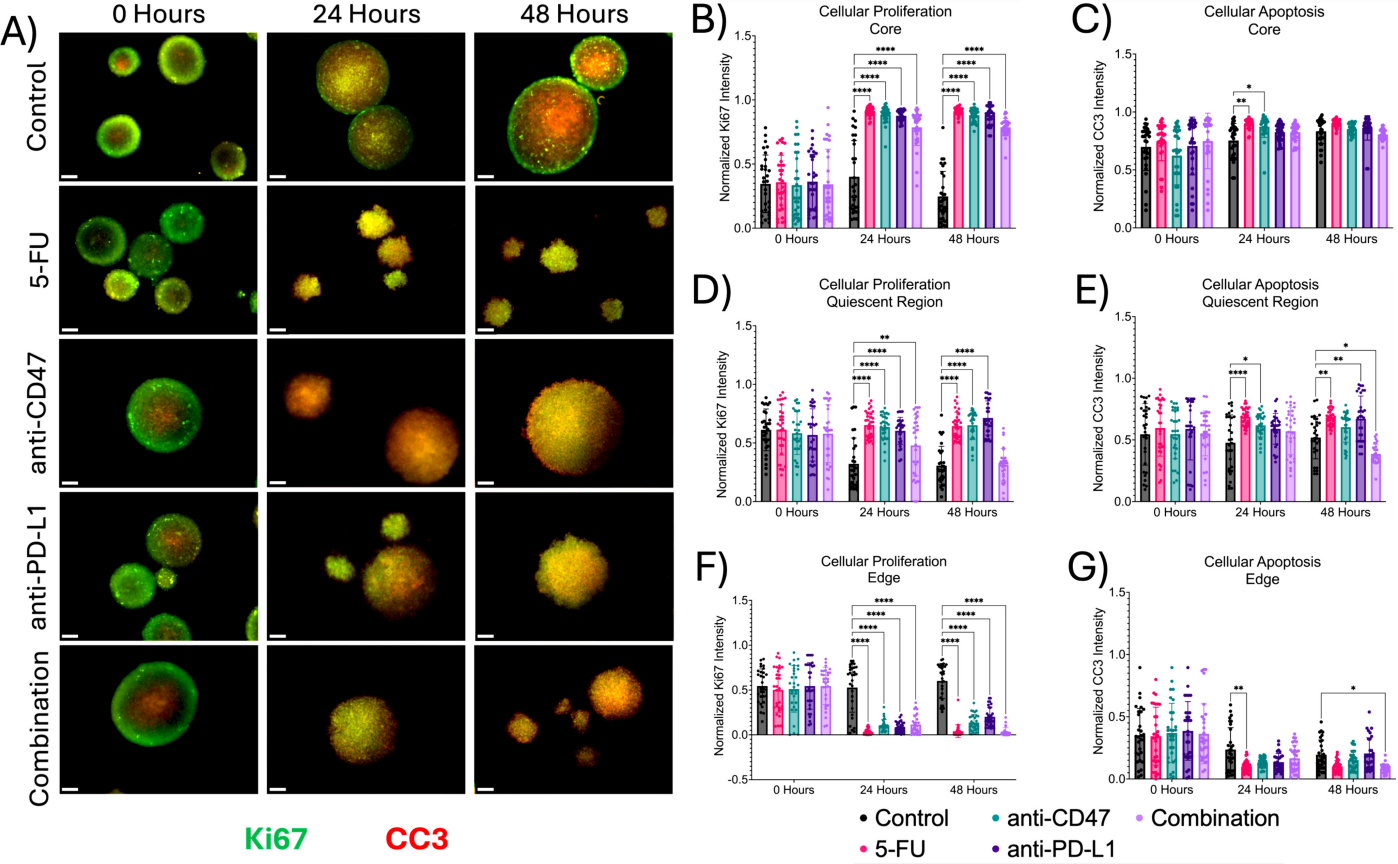
Significant changes were observed across spheroid regions between cellular proliferation and apoptosis before and after treatment. **(A)** Representative immunofluorescence images of cellular proliferation (Ki67) and apoptosis (CC3) across spheroid regions before and after treatment. **(B–G)** Normalized Ki67 and CC3 pixel intensities. *p ≤ 0.05, **p ≤ 0.01, ****p ≤ 0.0001. Plots were made in GraphPad Prism ®. Scale bars are 20 µm.

**Figure 5:**
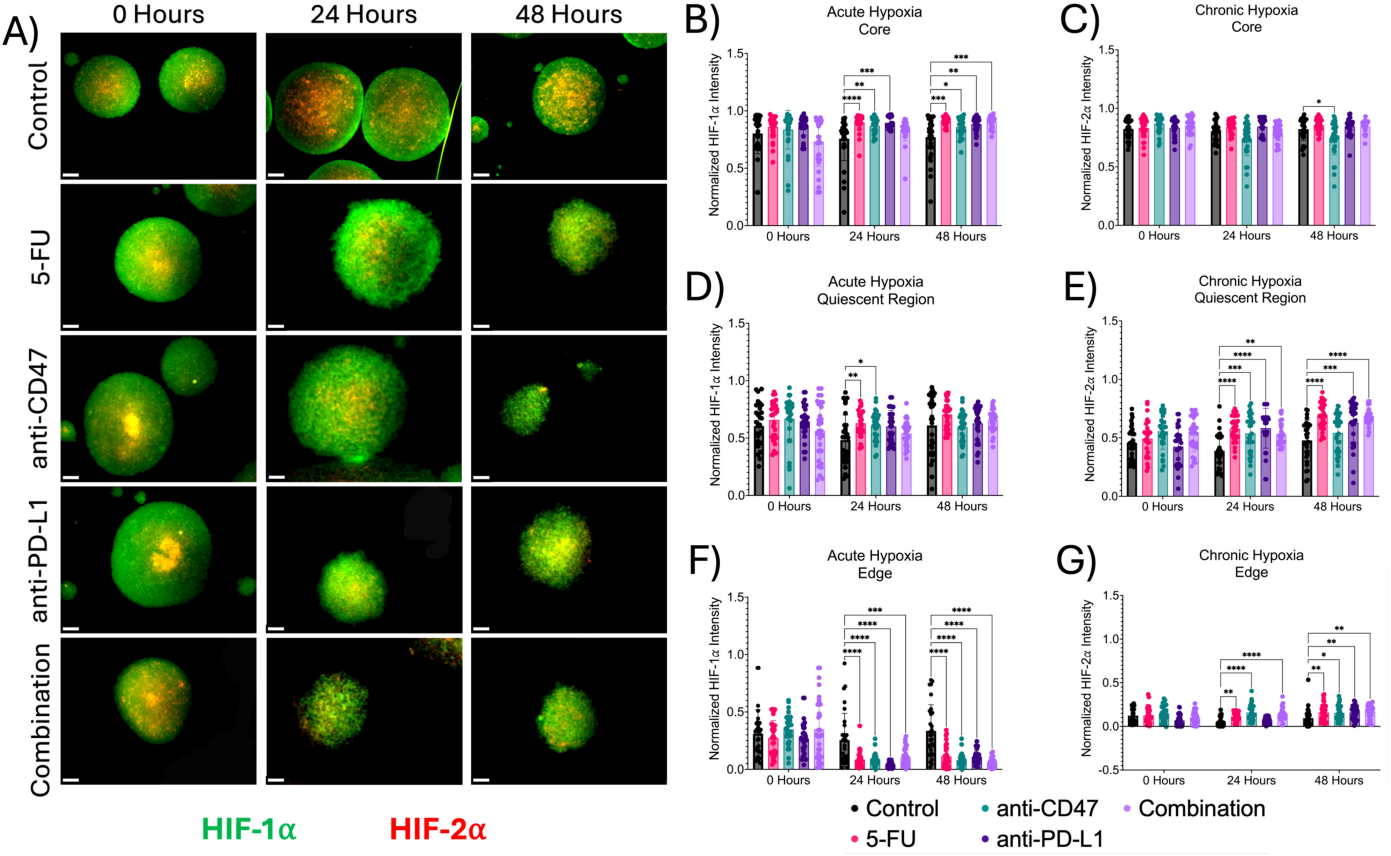
Significant changes were observed across spheroid regions between acute and chronic hypoxia before and after treatment. **(A)** Representative immunofluorescence images of acute (HIF-1α) and chronic (HIF-2α) hypoxia across spheroid regions before and after treatment. **(B-G)** Normalized HIF-1α and HIF-2α pixel intensities. *p ≤ 0.05, **p ≤ 0.01,***p ≤ 0.001, ****p ≤ 0.0001. Plots were made in GraphPad Prism ^®^. Scale bars are 20 µm.

Next, we investigated the effects of how treatment influences cellular proliferation and apoptosis, along with changes in hypoxia markers within the multicellular spheroid model across microregions (**Figures 4**, **5**). At the core, all structural markers showed an increase in normalized intensity over time compared to the control. For proliferation, 5-FU-treated spheroids saw normalized Ki67 pixel intensities of 0.912 ± 0.035 (p < 0.0001) and 0.912 ± 0.029 (p < 0.0001) 24 and 48 hours post-treatment, respectively. Anti-CD47-treated spheroids saw normalized Ki67 pixel intensities of 0.886 ± 0.064 (p < 0.0001) and 0.881 ± 0.050 (p < 0.0001) 24 and 48 hours post-treatment, respectively. Anti-PD-L1-treated spheroids saw normalized Ki67 pixel intensities of 0.877 ± 0.041 (p < 0.0001) and 0.882 ± 0.076 (p < 0.0001) 24 and 48 hours post-treatment, respectively. The combination group saw normalized Ki67 pixel intensities of 0.787 ± 0.141 (p < 0.0001) and 0.787 ± 0.069 (p < 0.0001) 24 and 48 hours post-treatment, respectively. For apoptosis, 5-FU-treated spheroids saw normalized CC3 pixel intensity values of 0.902 ± 0.042–24 hours post-treatment (p = 0.0010). Anti-CD47-treated spheroids saw normalized CC3 pixel intensity values of 0.873 ± 0.092 (p = 0.0148) 24 hours post-treatment. For acute hypoxia, 5-FU-treated spheroids showed normalized HIF-1α pixel intensity values of 0.900 ± 0.072–24 hours post-treatment (p < 0.0001) and 0.911 ± 0.037–48 hours post-treatment (p = 0.0001). Anti-CD47-treated spheroids showed normalized HIF-1α pixel intensity values of 0.869 ± 0.068–24 hours post-treatment (p = 0.0038) and 0.860 ± 0.079–48 hours post-treatment (p = 0.0403). Anti-PD-L1-treated spheroids showed normalized HIF-1α pixel intensity values of 0.896 ± 0.049–24 hours post-treatment (p = 0.0001) and 0.844 ± 0.062–48 hours post-treatment (p = 0.0033). The combination group showed normalized HIF-1α pixel intensity values of 0.906 ± 0.050–48 hours post-treatment (p = 0.0002). For chronic hypoxia, Anti-CD47-treated spheroids showed normalized HIF-2α pixel intensity values of 0.751 ± 0.149–48 hours post-treatment (p = 0.0170). Other significant differences were observed at 24 hours between the 5-FU and anti-CD47 groups (p = 0.0002) and between the anti-CD47 and anti-PD-L1 groups (p = 0.0001).

In the quiescent region, the addition of treatments slightly increased cellular apoptosis at 24 and 48 hours except for the combination group, while hypoxia markers increased over time. 5-FU-treated spheroids saw normalized Ki67 pixel intensities of 0.653 ± 0.111 (p < 0.0001) and 0.644 ± 0.118 (p < 0.0001) 24 and 48 hours after treatment, respectively. Anti-CD47-treated spheroids saw normalized Ki67 pixel intensities of 0.636 ± 0.109 (p < 0.0001) 24 hours after treatment and 0.651 ± 0.122 (p < 0.0001) 48 hours after treatment. Anti-PD-L1-treated spheroids saw normalized Ki67 pixel intensities of 0.604 ± 0.115–24 hours post-treatment (p < 0.0001) and 0.713 ± 0.145–48 hours post-treatment (p < 0.0001). The combination group saw normalized Ki67 pixel intensities of 0.479 ± 0.261 (p = 0.0062). For apoptosis, 5-FU-treated spheroids saw normalized CC3 pixel intensity values of 0.687 ± 0.072–24 hours post-treatment (p < 0.0001) and 0.683 ± 0.070–48 hours post-treatment (p = 0.0030). Anti-CD47-treated spheroids saw normalized CC3 pixel intensity values of 0.619 ± 0.095–24 hours post-treatment (p = 0.0155). Anti-PD-L1-treated spheroids saw normalized CC3 pixel intensity values of 0.672 ± 0.184 (p = 0.0070) 48 hours post-treatment. The combination group saw normalized CC3 pixel intensity values of 0.387 ± 0.078–48 hours post-treatment (p = 0.0288). For acute hypoxia, 5-FU-treated spheroids showed normalized HIF-1α pixel intensity values of 0.687 ± 0.072–24 hours post-treatment (p = 0.0075). Anti-CD47-treated spheroids showed normalized HIF-1α pixel intensity values of 0.619 ± 0.095–24 hours post-treatment (p = 0.0362). For chronic hypoxia, 5-FU-treated spheroids showed normalized HIF-2α pixel intensity values of 0.579 ± 0.109–24 hours post-treatment (p < 0.0001) and 0.689 ± 0.113–48 hours post-treatment (p < 0.0001). Anti-CD47-treated spheroids showed normalized HIF-2α pixel intensity values of 0.539 ± 0.165–24 hours post-treatment (p = 0.0010). Anti-PD-L1-treated spheroids showed normalized HIF-2α pixel intensity values of 0.583 ± 0.171–24 hours post-treatment (p < 0.0001) and 0.631 ± 0.185–48 hours post-treatment (p = 0.0007). The combination group showed normalized HIF-2α pixel intensity values of 0.531 ± 0.097–24 hours post-treatment (p = 0.0021) and 0.688 ± 0.074–48 hours post-treatment (p < 0.0001).

At the edge, proliferation and apoptosis decreased over time, while acute and chronic hypoxia increased over time, regardless of treatment. For proliferation, 5-FU-treated spheroids saw normalized Ki67 pixel intensities of 0.033 ± 0.033 (p < 0.0001) 24 hours post-treatment and 0.041 ± 0.072 (p < 0.0001) 48 hours post-treatment. Anti-CD47-treated spheroids saw normalized Ki67 pixel intensities of 0.108 ± 0.063–24 hours post-treatment (p < 0.0001) and 0.137 ± 0.091–48 hours post-treatment (p < 0.0001). The combination group saw normalized Ki67 pixel intensities of 0.116 ± 0.107–24 hours post-treatment (p < 0.0001) and 0.033 ± 0.046–48 hours post-treatment (p < 0.0001). For apoptosis, 5-FU-treated spheroids saw normalized CC3 pixel intensity values of 0.101 ± 0.046–24 hours post-treatment (p = 0.0067). The combination group saw normalized CC3 pixel intensity values of 0.167 ± 0.105–24 hours post-treatment (p = 0.0465). For acute hypoxia, 5-FU-treated spheroids showed normalized HIF-1α pixel intensity values of 0.085 ± 0.073–24 hours post-treatment (p < 0.0001) and 0.113 ± 0.098–48 hours post-treatment (p < 0.0001). Anti-CD47-treated spheroids showed normalized HIF-1α pixel intensity values of 0.082 ± 0.055–24 hours post-treatment (p < 0.0001) and 0.081 ± 0.057–48 hours post-treatment (p < 0.0001). Anti-PD-L1-treated spheroids showed normalized HIF-1α pixel intensity values of 0.040 ± 0.026–24 hours post-treatment (p < 0.0001) and 0.110 ± 0.058–48 hours post-treatment (p < 0.0001). The combination group showed normalized HIF-1α pixel intensity values of 0.104 ± 0.079–24 hours post-treatment (p = 0.0006) and 0.059 ± 0.044–48 hours post-treatment (p < 0.0001). For chronic hypoxia, 5-FU-treated spheroids showed normalized HIF-2α pixel intensity values of 0.113 ± 0.046–24 hours post-treatment (p = 0.0092) and 0.160 ± 0.098–48 hours post-treatment (p = 0.0099). Anti-CD47-treated spheroids showed normalized HIF-2α pixel intensity values of 0.159 ± 0.089–24 hours post-treatment (p < 0.0001) and 0.154 ± 0.084–48 hours post-treatment (p = 0.0256). Anti-PD-L1-treated spheroids showed normalized HIF-2α pixel intensity values of 0.161 ± 0.077–48 hours post-treatment (p = 0.0089). The combination group showed normalized HIF-2α pixel intensity values of 0.141 ± 0.070–24 hours post-treatment (p < 0.0001) and 0.167 ± 0.077–48 hours post-treatment (p = 0.0034). In addition to comparing structural markers from each treatment group to the control group, statistical comparisons were made within each group (**Supplementary Figures 4**, **5** and **Supplementary Tables 2**, **3**).

### 3.4 Treatments show slight changes in immune checkpoint expression across spheroid microregions

**Figure 6:**
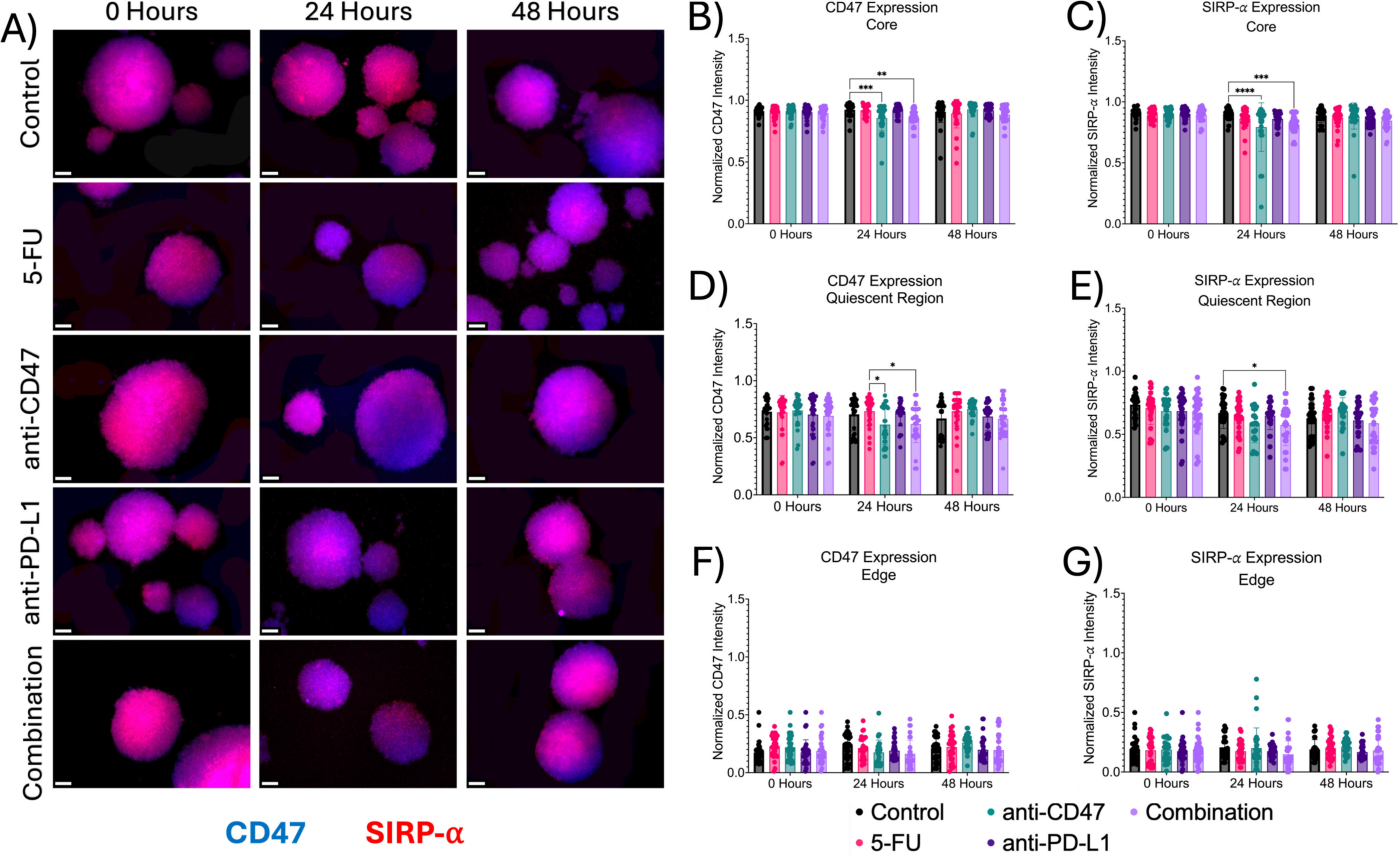
Significant changes were observed across spheroid regions in CD47 and SIRP-α expression before and after treatment. **(A)** Representative immunofluorescence images of CD47 and SIRP-α across spheroid regions before and after treatment. **(B-G)**: Normalized CD47 and SIRP-α pixel intensities *p ≤ 0.05, **p ≤ 0.01,***p ≤ 0.001,****p ≤ 0.0001. Plots were made in GraphPad Prism ^®^. Scale bars are 20 µm.

**Figure 7:**
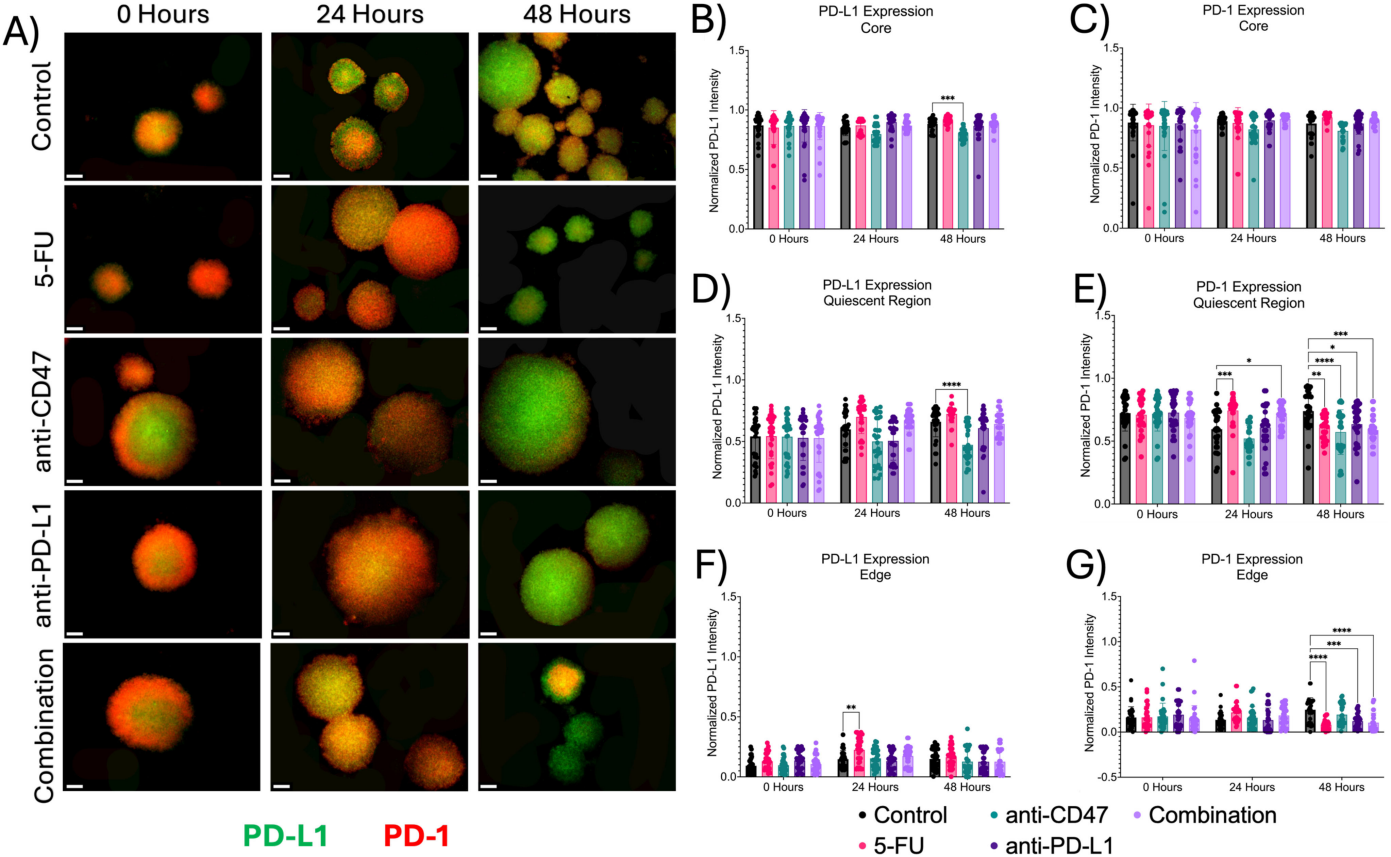
Significant changes were observed across spheroid regions in PD-L1 and PD-1 expression before and after treatment. **(A)** Representative immunofluorescence images of PD-L1 and PD-1 across spheroid regions before and after treatment. **(B-G)** Normalized PD-L1 and PD-1 pixel intensities. *p ≤ 0.05, **p ≤ 0.01,***p ≤ 0.001,****p ≤ 0.0001. Plots were made in GraphPad Prism ^®^. Scale bars are 20 µm.

Next, we investigated the effects of how treatment influences immune checkpoint expression across microregions (**Figures 6**, **7**). At the core, all immune checkpoint markers show slight changes in expression, regardless of treatment. For CD47, anti-CD47-treated spheroids showed normalized CD47 pixel intensity values of 0.855 ± 0.103–24 hours post-treatment (p = 0.0005). The combination group showed normalized CD47 pixel intensity values of 0.859 ± 0.064–24 hours post-treatment (p = 0.0016). For SIRP-α, anti-CD47-treated spheroids showed normalized SIRP-α pixel intensity values of 0.793 ± 0.199–24 hours post-treatment (p < 0.0001). The combination group showed normalized SIRP-α pixel intensity values of 0.815 ± 0.074–24 hours post-treatment (p = 0.0004). For PD-L1 expression, anti-CD47-treated spheroids showed normalized PD-L1 pixel intensity values of 0.793 ± 0.047–48 hours post-treatment (p = 0.0003). For PD-1 expression, one significant difference was observed at 24 hours between the 5-FU and anti-CD47 groups (p = 0.0044).

In the quiescent region, for CD47 expression, no statistical differences were observed. For SIRP-α, normalized pixel intensity values showed a slight decrease in expression across treatment groups, with the combination group showing the largest decrease. Anti-PD-L1-treated spheroids showed normalized SIRP-α pixel intensity values of 0.647 ± 0.110–24 hours post-treatment (p = 0.0456). Also, in the quiescent region, all treatments except for the anti-CD47 group showed increases in PD-L1 expression, while PD-1 expression showed an initial increase in expression in all treatment groups except for the anti-CD47 group. For PD-L1 expression, anti-CD47-treated spheroids showed normalized PD-L1 pixel intensity values of 0.473 ± 0.139–48 hours post-treatment (p < 0.0001). For PD-1 expression, 5-FU-treated spheroids showed normalized PD-1 pixel intensity values of 0.743 ± 0.122–24 hours post-treatment (p = 0.0003) and 0.608 ± 0.098–48 hours post-treatment (p = 0.0023). Anti-CD47-treated spheroids showed normalized PD-1 pixel intensity values of 0.572 ± 0.178–48 hours post-treatment (p < 0.0001). Anti-PD-L1-treated spheroids showed normalized PD-1 pixel intensity values of 0.634 ± 0.151–48 hours post-treatment (p = 0.0267). The combination group showed normalized PD-1 pixel intensity values of 0.697 ± 0.086–24 hours post-treatment (p = 0.0314) and 0.588 ± 0.103–48 hours post-treatment (p = 0.0002).

At the edge, treatments showed slight changes in immune checkpoint expression, regardless of treatment. For CD47 and SIRP-α expression, no significant differences were observed. For PD-L1 expression, 5-FU-treated spheroids showed normalized PD-L1 pixel intensity values of 0.225 ± 0.109–24 hours post-treatment (p = 0.0022). For PD-1 expression, 5-FU-treated spheroids showed normalized PD-1 pixel intensity values of 0.082 ± 0.054–48 hours post-treatment (p < 0.0001). Anti-PD-L1-treated spheroids showed normalized PD-1 pixel intensity values of 0.121 ± 0.086–48 hours post-treatment (p = 0.0006). The combination group showed normalized PD-1 pixel intensity values of 0.104 ± 0.107–48 hours post-treatment (p < 0.0001). In addition to comparing immune checkpoint expression markers for each treatment group to the control group, statistical comparisons were made within each group (**Supplementary Figures 6**, **7** and **Supplementary Tables 4**, **5**).

### 3.5 Treatments show significant increases in optical redox ratio across microregions over time

**Figure 8:**
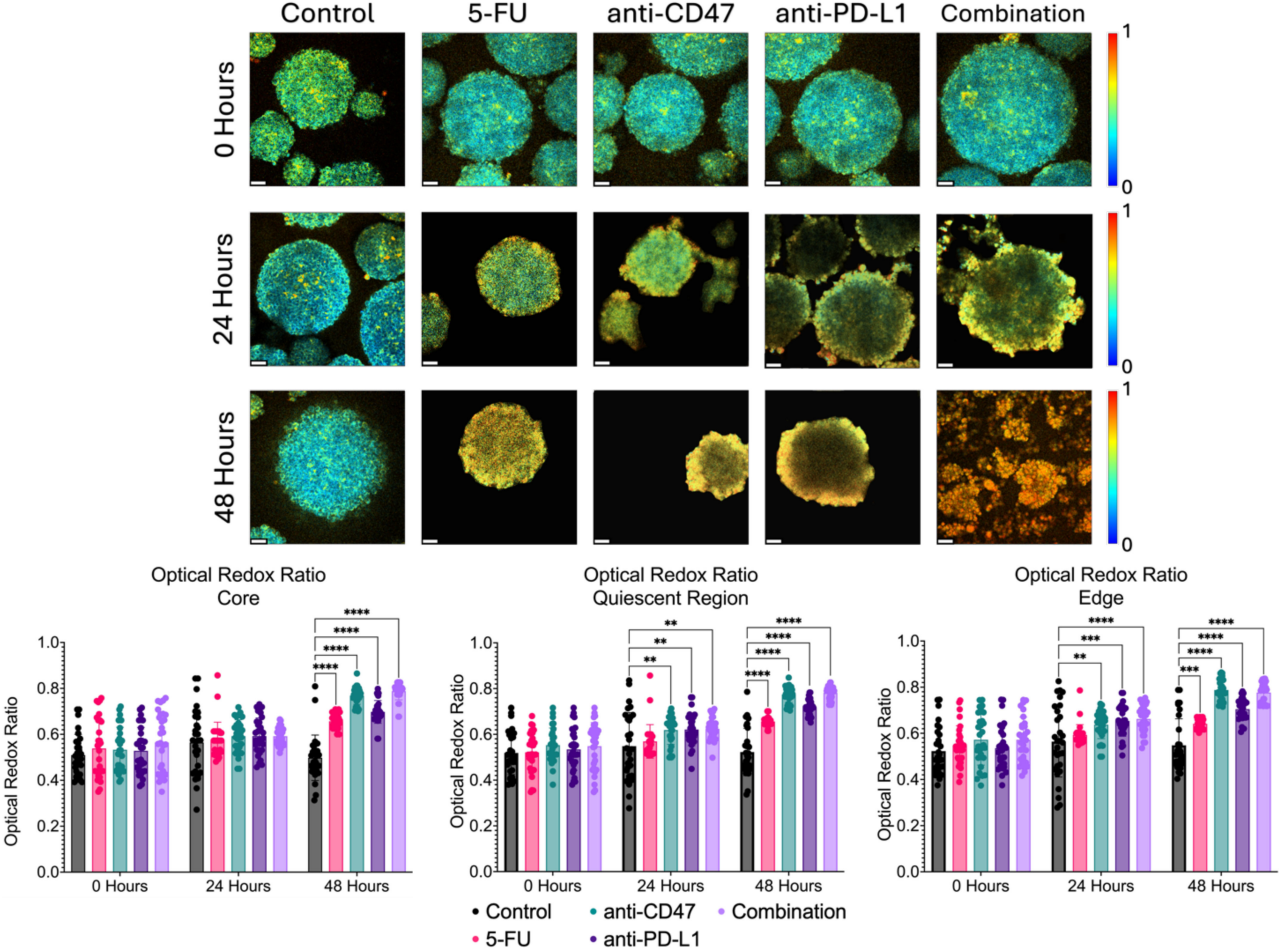
Significant changes were observed across spheroid regions in the metabolic optical redox ratio before and after treatment. Top: Representative optical redox maps before and after treatment. Bottom: Optical redox ratios across spheroid regions. **p ≤ 0.01,***p ≤ 0.001,****p ≤ 0.0001. Plots were made in GraphPad Prism ^®^. Scale bars are 20 µm.

In addition to investigating structural changes after treatment, we wanted to investigate how the treatment modalities alter the metabolic state of the multicellular spheroids. By measuring the autofluorescence of NADH and FAD, the optical redox ratio can be used to estimate the oxidation-reduction state (**Figure 8**). More specifically, at the core, the optical redox ratio did not show any significant changes until after 48 hours of treatment across all treatment groups. 5-FU-treated spheroids showed optical redox values of 0.657 ± 0.032–48 hours post-treatment (p < 0.0001). Anti-CD47-treated spheroids showed optical redox values of 0.769 ± 0.038–48 hours post-treatment (p < 0.0001). Anti-PD-L1-treated spheroids showed optical redox values of 0.698 ± 0.050–48 hours post-treatment (p < 0.0001). The combination group showed optical redox values of 0.788 ± 0.038–48 hours post-treatment (p < 0.0001).

In the quiescent region, the optical redox ratio in all the treatment groups increased over time compared to the control. 5-FU-treated spheroids showed optical redox values of 0.656 ± 0.023–48 hours post-treatment (p < 0.0001). Anti-CD47-treated spheroids showed optical redox values of 0.619 ± 0.062–24 hours post-treatment (p = 0.0056) and 0.775 ± 0.036–48 hours post-treatment (p < 0.0001). Anti-PD-L1-treated spheroids showed optical redox values of 0.621 ± 0.070–24 hours post-treatment (p = 0.0037) and 0.717 ± 0.035–48 hours post-treatment (p < 0.0001). The combination group showed optical redox values of 0.624 ± 0.048–24 hours post-treatment (p = 0.0023) and 0.781 ± 0.028–48 hours post-treatment (p < 0.0001).

At the edge, the optical redox ratio in all the treatment groups increased over time compared to the control. 5-FU-treated spheroids showed optical redox values of 0.641 ± 0.022–48 hours post-treatment (p = 0.0002). anti-CD47-treated spheroids showed optical redox values of 0.638 ± 0.061–24 hours post-treatment (p = 0.0069) and 0.788 ± 0.044–48 hours post-treatment (p < 0.0001). Anti-PD-L1-treated spheroids showed optical redox values of 0.654 ± 0.067–24 hours post-treatment (p = 0.0004) and 0.705 ± 0.049–48 hours post-treatment (p < 0.0001). The combination group showed optical redox values of 0.663 ± 0.060–24 hours post-treatment (p < 0.0001) and 0.777 ± 0.044–48 hours post-treatment (p < 0.0001). In addition to comparing the optical redox ratio to the control group, statistical comparisons were made within each group to determine if there were significant differences across time (**Supplementary Figure 8** and **Supplementary Table 6**).

### 3.6 Treatments show significant decreases in mean NADH lifetime, but only slight changes in A1/A2 over time

**Figure 9:**
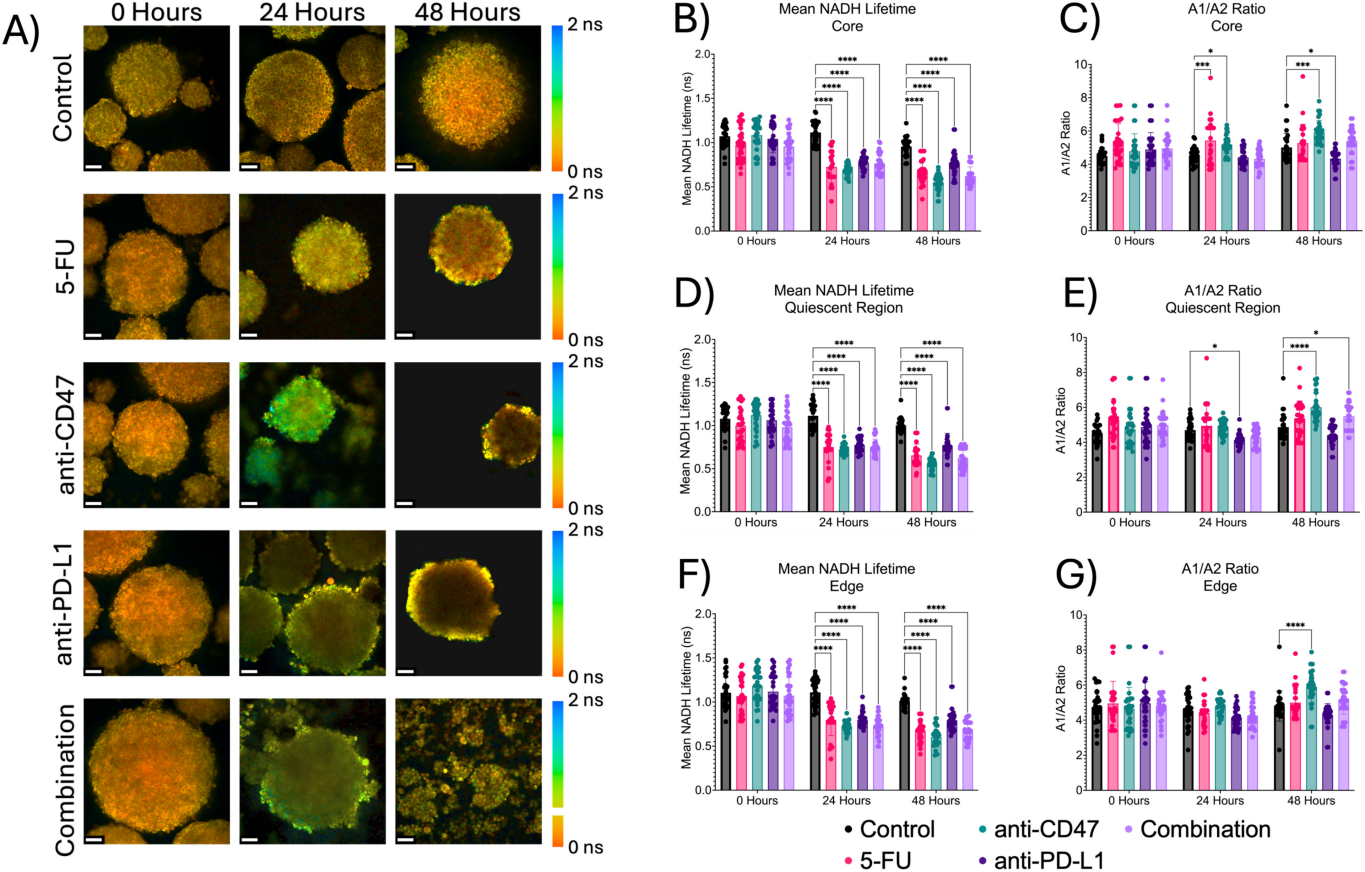
Significant changes were observed across spheroid regions between mean NADH lifetime and A1/A2 ratio before and after treatment. Top: Representative mean NADH lifetime images across spheroid regions before and after treatment. Middle: Mean NADH lifetime values across regions. Bottom: A1/A2 ratios across spheroid regions. *p ≤ 0.05, ***p ≤ 0.001,****p ≤ 0.0001. Plots were made in GraphPad Prism ^®^. Scale bars are 20 µm.

In addition to the optical redox ratio, two fluorescence lifetime imaging (FLIM) metrics (mean NADH lifetime) and the A1/A2 ratio were used to quantify the contributions of protein-bound and unbound NADH to the overall metabolic state of the multicellular spheroids (**Figure 9**). At the core, the mean NADH lifetime significantly decreases over time, while A1/A2 shows mixed but significant changes over time after treatment. For mean NADH lifetime, 5-FU-treated spheroids showed mean NADH lifetime values of 0.726 ± 0.181 ns 24 hours post-treatment (p < 0.0001) and 0.665 ± 0.133 ns 48 hours post-treatment (p < 0.0001). Anti-CD47-treated spheroids showed mean NADH lifetime values of 0.691 ± 0.062 ns 24 hours post-treatment (p < 0.0001) and 0.552 ± 0.091 ns 48 hours post-treatment (p < 0.0001). Anti-PD-L1-treated spheroids showed mean NADH lifetime values of 0.771 ± 0.072 ns 24 hours post-treatment (p < 0.0001) and 0.773 ± 0.135 ns 48 hours post-treatment (p < 0.0001). The combination group showed mean NADH lifetime values of 0.766 ± 0.110 ns 24 hours post-treatment (p < 0.0001) and 0.623 ± 0.104 ns 48 hours post-treatment (p < 0.0001). For A1/A2, 5-FU-treated spheroids showed A1/A2 ratios of 5.423 ± 1.336–24 hours post-treatment (p = 0.0005). Anti-CD47-treated spheroids showed A1/A2 ratios of 5.195 ± 0.567–24 hours post-treatment (p = 0.0210) and 5.922 ± 0.714–48 hours post-treatment (p = 0.0003). Anti-PD-L1-treated spheroids showed A1/A2 ratios of 4.334 ± 0.516–48 hours post-treatment (p = 0.0155).

### 3.7 Correlations between structural and metabolic markers

A Pearson r correlation matrix was created to investigate relationships between structural and metabolic markers within the spheroid model across the micro-regions (**Supplementary Tables 8**-**10**). At the core, several significant correlations were observed between CD80 and CD206 (r = 0.61), Ki67 (r = 0.76), optical redox ratio (r = 0.47), and mean NADH lifetime (r = -0.66); CD206 and Ki67 (r = 0.48) and mean NADH lifetime (r = -0.43); Ki67 and mean NADH lifetime (r = -0.65); CD47 and SIRP-α (r = 0.53); optical redox ratio and mean NADH lifetime (r = -0.48); and mean NADH lifetime and A1/A2 (r = -0.52).

Significant correlations were also observed at the quiescent region between CD80 and CD206 (r = -0.43), SIRP-α (r = -0.45), optical redox ratio (r = 0.55), and mean NADH lifetime (r = -0.58); CD206 and optical redox ratio (r = -0.63) and mean NADH lifetime (r = 0.58); Ki67 and CC3 (r = 0.75); CC3 and PD-L1 (r = -0.41); HIF-1α and HIF-2α (r = 0.77); HIF-2α and PD-1 (r = -0.40), optical redox ratio (r = 0.50), and mean NADH lifetime (r = -0.63); CD47 and SIRP-α (r = 0.78); PD-1 and PD-L1 (r = 0.54), optical redox ratio (r = -0.46), and mean NADH lifetime (r = 0.48); optical redox ratio and mean NADH lifetime (r = -0.84); and mean NADH lifetime and A1/A2 (r = -0.66).

At the edge, significant correlations were observed at the edge between CD80 and CD206 (r = 0.84), Ki67 (r = 0.72), HIF-1α (r = 0.53), optical redox ratio (r = -0.54), and mean NADH lifetime (r = 0.70); CD206 and Ki67 (r = 0.67), optical redox ratio (r = -0.45), and mean NADH lifetime (r = 0.64); Ki67 and CC3 (r = 0.52), HIF-1α (r = 0.53), and mean NADH lifetime (r = 0.61); CC3 and mean NADH lifetime (r = 0.43); HIF-1α and mean NADH lifetime (r = 0.42); CD47 and SIRP-α (r = 0.40); PD-1 and PD-L1 (r = 0.40); and optical redox ratio and mean NADH lifetime (r = -0.42).

### 3.8 Immunofluorescence of cellular expression via 2D co-culture reveals increases in M1 macrophage polarization after ICD induction in combination with immune checkpoint inhibitors

**Figure 10:**
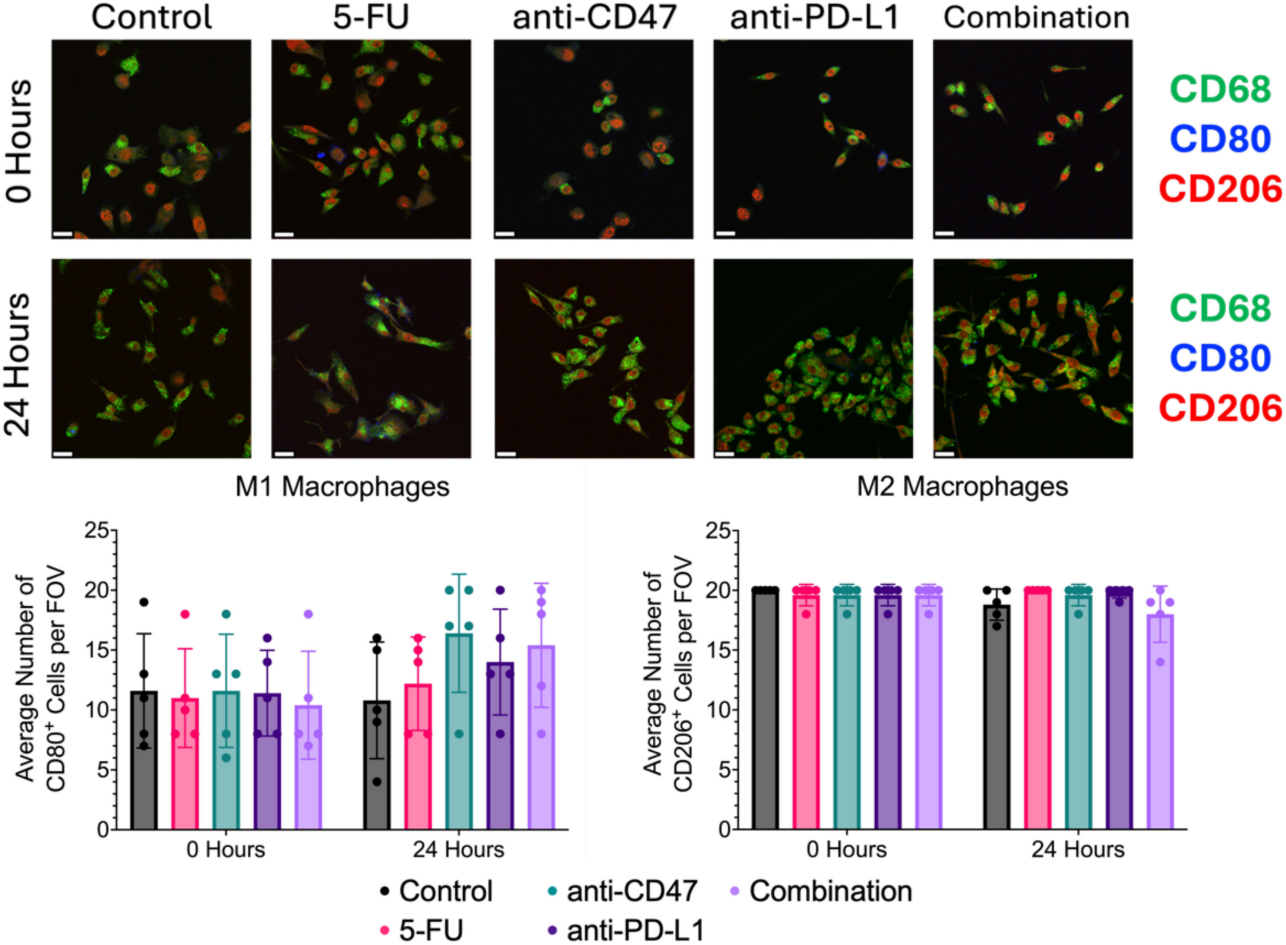
Immunofluorescence shows changes in M1 and M2 macrophage markers before and after treatment. Top: Representative immunofluorescence images of macrophage markers (CD80: M1 and CD206: M2) before and after treatment. Bottom: Plots showing average number of macrophages with CD80 or CD206 expression per field of view. Plots were made in GraphPad Prism ^®^. Scale bars are 20 µm. n = 5.

Using conventional 2D CT26/RAW 264.7 co-cultures, immunofluorescence staining of M1 (CD80), M2 (CD206), and pan-macrophage (CD68) surface markers was used to quantify changes in macrophage phenotypes and ICD induction via chemotherapy and exposure to immune checkpoint inhibitors (**Figure 10**). Before treatment, there was an average of approximately 11 macrophages expressing CD80 with approximately 11 macrophages expressing CD206. After 24 hours, the anti-CD47 group showed the highest increase in the average number of macrophages expressing CD80 (~16 macrophages) compared to the other treatment groups. Also, the combination group showed the greatest decrease in the average number of macrophages expressing CD206 (~18 macrophages) compared to the other treatment groups after treatment. No statistical differences were observed between the treatment groups and the control group. Overall, results indicate that after 24 hours, the anti-CD47-treated cultures displayed the highest percentage of macrophages displaying M1 surface markers; however, the cultures in the combination group were the only treatment group to decrease the percentage of macrophages displaying M2 surface markers.

### 3.9 Immunofluorescence of cellular expression via 2D co-culture reveals changes in immune checkpoint expression after ICD induction in combination with immune checkpoint inhibitors

**Figure 11:**
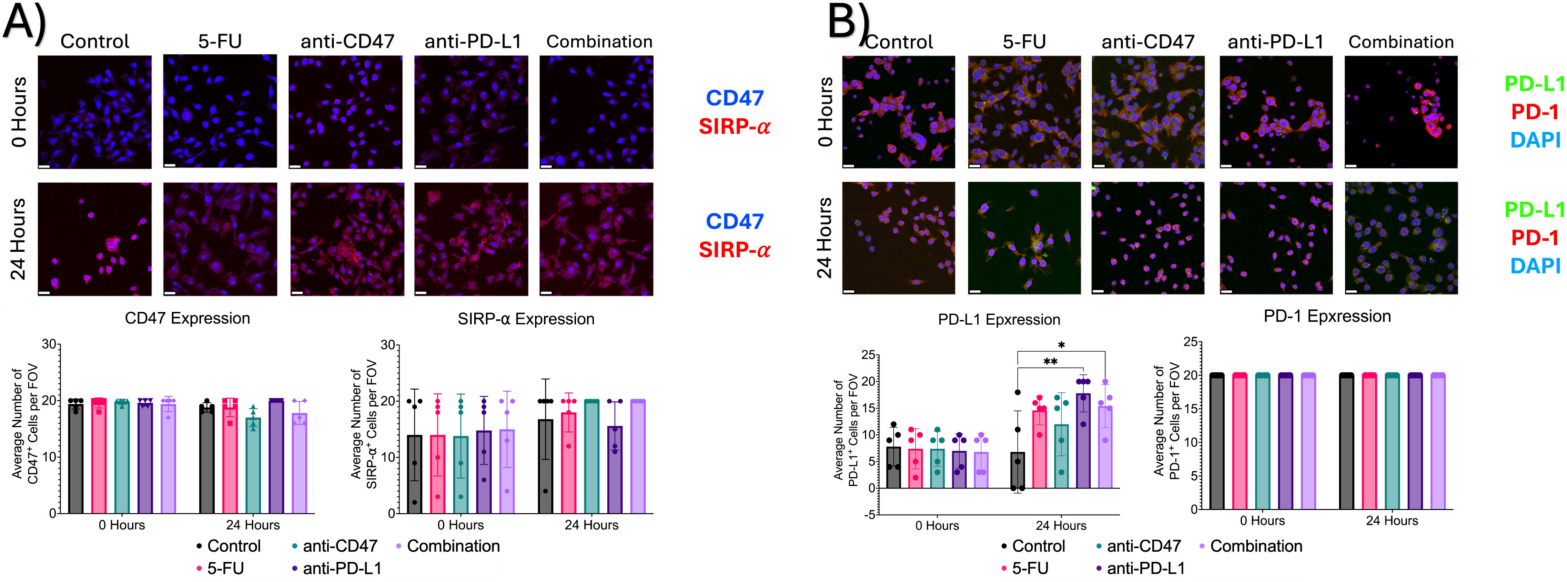
Immunofluorescence shows changes in immune checkpoint expression before and after treatment. **(A)** Top: Representative immunofluorescence images of CD47 and SIRP-α before and after treatment. Bottom: Plots showing average number of macrophages with CD47 or SIRP-α expression per field of view. **(B)** Top: Representative immunofluorescence images of PD-1 and PD-L1 before and after treatment. Bottom: Plots showing average % of macrophages with PD-L1 or PD-1 expression per field of view. *p ≤ 0.05, **p ≤ 0.01. Plots were made in GraphPad Prism ^®^. Scale bars are 20 µm. n = 5.

Additional immunofluorescence was used to quantify macrophage and cancer cell immune checkpoint expression and ICD induction via chemotherapy and exposure to immune checkpoint inhibitors (**Figure 11**). Before treatment, all groups showed an average number of ~19 cells expressing CD47, ~14 cells expressing SIRP-α, ~7 cells expressing PD-L1, and approximately 20 cells expressing PD-1. After 24 hours, the anti-CD47 and combination groups showed the greatest decrease in CD47 expression and the greatest increase in SIRP-α expression. Interestingly, the anti-PD-L1 and combination groups showed the greatest increase in PD-L1 expression.

### 3.10 Treated 2D co-cultures reveal changes in ICD hallmarks after treatment

**Figure 12:**
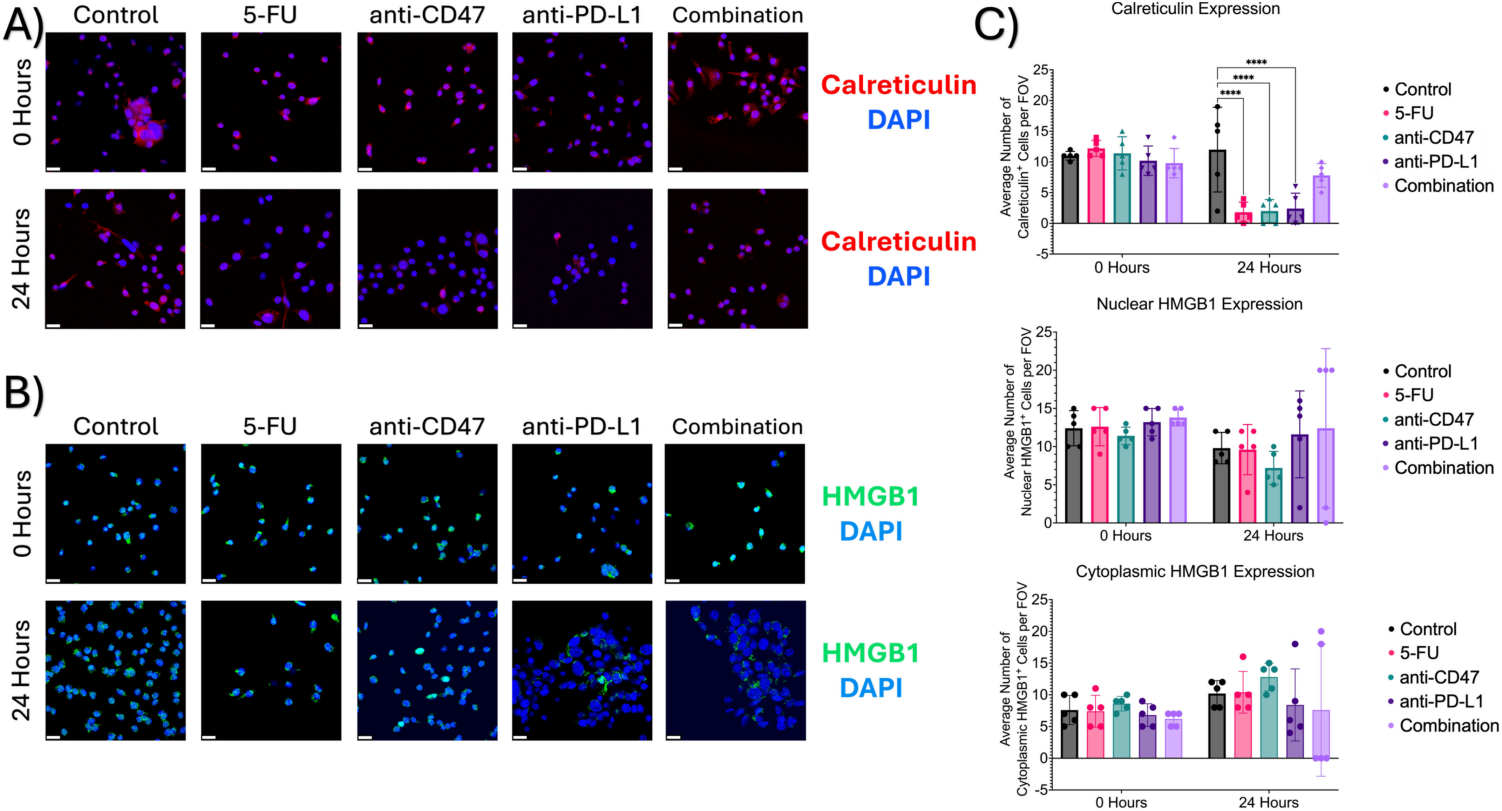
Immunofluorescence shows changes in ICD markers (calreticulin and HMGB1) expression before and after treatment. **(A)** Representative immunofluorescence images of cytoplasmic calreticulin expression before and after treatment. **(B)** Representative immunofluorescence images of nuclear and cytoplasmic HMGB1 before and after treatment. **(C)** Plots showing average number of cells with calreticulin expression (top), nuclear HMGB1 expression (middle), and cytoplasmic HMGB1 expression (bottom). ****p ≤ 0.0001. Plots were made in GraphPad Prism ®. Scale bars are 20 µm. n =5.

To investigate a hallmark of ICD induction, immunofluorescence was used to 1) quantify the release of calreticulin into the cytoplasm and 2) quantify the translocation of HMGB1 from the nuclei to the cytoplasm (**Figure 12**). Before treatment, it was found that an average of approximately 11 cells expressed calreticulin, 12 cells showed nuclear HMGB1 expression, and 7 cells showed cytoplasmic HMGB1 expression. After 24 hours of treatment, all treatment groups except for the combination group showed a large decrease in cellular calreticulin expression (average of ~2 cells and 8 cells, respectively). Significant differences were observed between the control group and the following treatment groups: 5-FU (p = 0.0010), anti-CD47 (p = 0.0013), and anti-PD-L1 (p = 0.0022). It was also found that nuclear HMGB1 expression decreased; however, the combination group showed the smallest decrease in cells expressing nuclear HMGB1 (~12 cells) compared to the other treatment groups. Interestingly, 5-FU and anti-CD47-treated cultures showed the highest average number of cells expressing cytoplasmic HMGB1 (~10 cells and 12 cells, respectively).

### 3.11 Treated 2D co-cultures reveal small changes in macrophage-mediated phagocytosis of cancer cells

**Figure 13:**
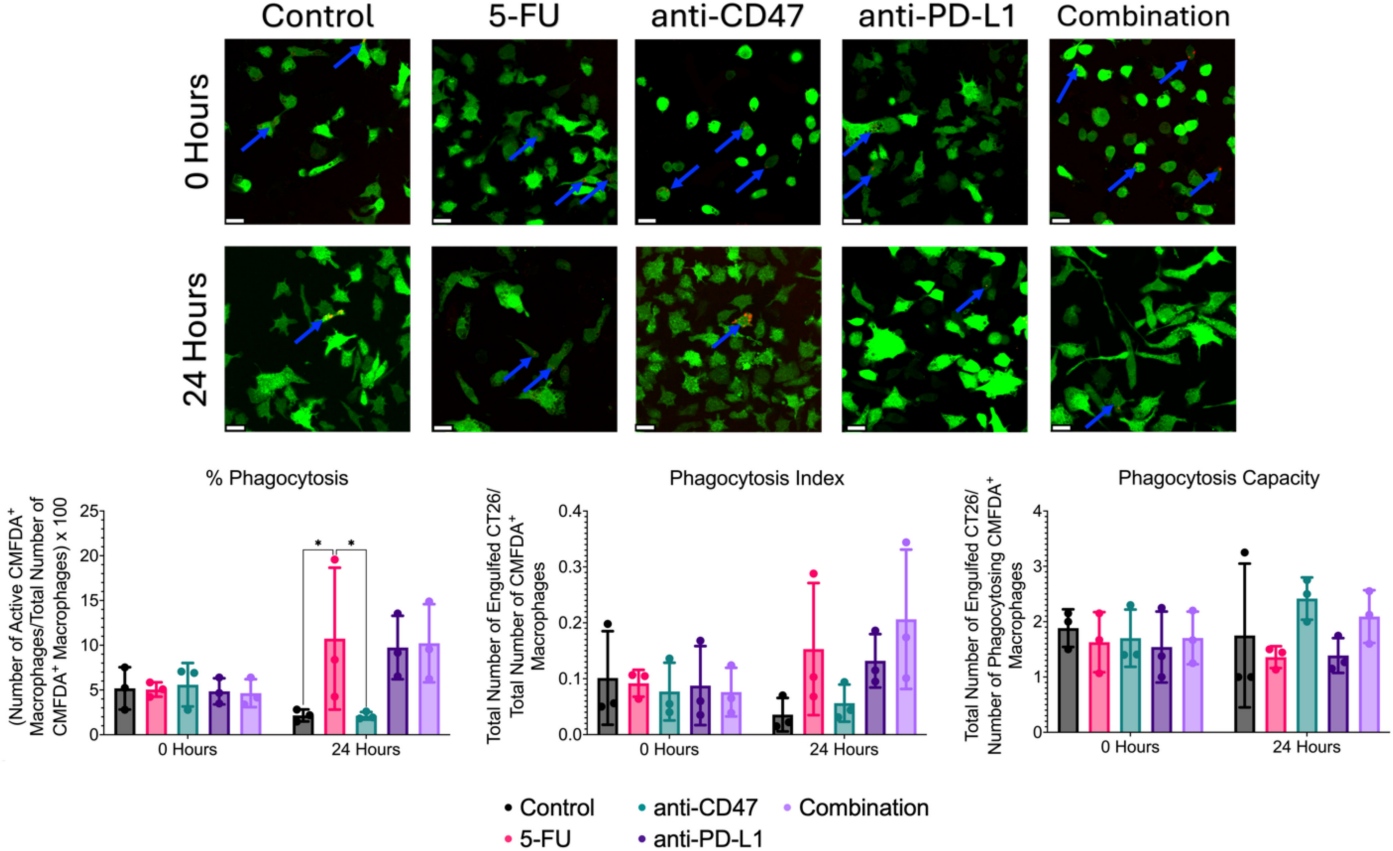
Immunofluorescence-based phagocytosis assay shows small changes in macrophage-mediated phagocytosis of CT26 cells before and after treatment. Top: Representative immunofluorescence images of CMFDA^+^ RAW 264.7 macrophages (green) and phagocytosed pHrodo-SE stained CT26 cells (red) before and after treatment. Blue arrows indicate positive phagocytosis of CT26 cells by RAW 264.7 macrophages. Bottom: Plots showing % phagocytosis, phagocytosis index, and phagocytosis capacity. *p ≤ 0.05. Plots were made in GraphPad Prism ^®^. Scale bars are 20 µm.

Lastly, we investigated whether the chosen treatments would show changes in macrophage-mediated phagocytosis of CT26 cells within our 2D co-cultures (**Figure 13**). Co-cultures in the control group showed a decrease in % phagocytosis from 5.181 ± 2.363% to 2.154 ± 0.671%. After 24 hours of treatment, increases in % phagocytosis were observed in the 5-FU (10.733 ± 7.926%), anti-PD-L1 (9.745 ± 3.545%), and combination (10.220 ± 4.368%) groups, with the 5-FU group showing the highest increase (5.683%). Interestingly, after 24 hours, the anti-CD47 group showed a decrease in % phagocytosis (~3%). Two significant differences were observed between the untreated co-cultures and 5-FU treated co-cultures (p = 0.0366) and 5-FU treated co-cultures and anti-CD47 treated co-cultures (p = 0.0355).

Although no significant differences were observed for the phagocytosis index, untreated co-cultures showed a decrease in phagocytosis index after 24 hours (0.101 ± 0.084 to 0.036 ± 0.030). After 24 hours of treatment, increases in phagocytosis index increased in the 5-FU (0.153 ± 0.118), anti-PD-L1 (0.132 ± 0.048), and combination groups (0.206 ± 0.125), with the combination group showing the highest increase (66%). Anti-CD47-treated cultures showed a 27% decrease in phagocytosis index after 24 hours (0.077 ± 0.052 to 0.056 ± 0.033).

Again, no significant differences were observed for phagocytosis capacity; untreated co-cultures showed a slight decrease in phagocytosis capacity after 24 hours (1.883 ± 0.340 to 1.750 ± 1.299). After 24 hours of treatment, increases in phagocytosis capacity were observed in the anti-CD47 (2.417 ± 0.382) and combination (2.092 ± 0.477) groups with the anti-CD47 group showing the greatest increase (~42%). Interestingly, the 5-FU (1.359 ± 0.198) and anti-PD-L1 (1.389 ± 0.315) groups showed a decrease in phagocytosis capacity. Overall, results indicate that although chemotherapy increased the percentage of macrophages performing phagocytosis, the combination group showed the highest number of engulfed cancer cells in the field of view of macrophages after 24 hours of treatment.

### 3.12 Correlations between cellular expression, ICD hallmarks, and phagocytic function in 2D co-cultures

A Pearson r correlation matrix was created to investigate relationships between cellular markers, ICD hallmarks, and phagocytosis metrics within the 2D co-culture model (**Supplementary Table 11**). Significant correlations were observed between CD80 and the following markers: calreticulin (r = -0.692, p = 0.0028), CD47 (r = -0.725, p = 0.018), SIRP-α (r = 0.784, p = 0.007), and PD-L1 (r = 0.730, p = 0.0017). All listed correlations are considered strong. Two additional strong and significant correlations were observed between CD47 and nuclear HMGB1 (r = 0.655, p = 0.040) and cytoplasmic HMGB1 (r = -0.655, p = 0.040). Another strong and significant correlation was observed between CD47 and SIRP-α (r = -0.908, p < 0.0001). Significant correlations were also seen between PD-L1 and calreticulin (r = -0.830, p = 0.003). When comparing phagocytosis metrics, significant correlations were observed between PD-L1 and % phagocytosis (r = 0.761, p = 0.011) and phagocytosis index (r = 0.719, p = 0.019). Another significant correlation was observed between CD47 and phagocytosis capacity (r = -0.809, p = 0.005). Interestingly, a correlation was also observed between % phagocytosis and phagocytosis index (r = 0.918, p < 0.0001).

### 3.13 Correlations between 3D spheroid edge and 2D co-culture model

Pearson r correlations were used to determine relationships between structural and metabolic metrics at the spheroid edge with cellular expression, ICD hallmarks, and phagocytosis metrics from the 2D co-culture model that could be used to predict cellular behavior (**Table 2**). When comparing the 3D optical imaging metrics to the 2D ICD and phagocytosis metrics, the following significant correlations were found: optical redox ratio vs. calreticulin (r = -0.713, p = 0.021), mean NADH lifetime vs. calreticulin (r = 0.858, p = 0.001), and A1/A2 ratio vs. % phagocytosis (r = -0.713, p = 0.021). When comparing 3D structural metrics to 2D ICD metrics, the following significant correlations were found: Ki67 vs. calreticulin (r = 0.915, p < 0.0001), CC3 vs. nuclear HMGB1 (r = 0.678, p = 0.031), CC3 vs. cytoplasmic HMGB1 (r = -0.678, p = 0.031), CC3 vs. calreticulin (r = 0.853, p = 0.002), and HIF-1α vs. calreticulin (r = 0.879, p = 0.001).

**Table 2:** Summary of Pearson Correlations r- values and p-values of 3D Structural and Metabolic Metrics at Spheroid Edge and 2D Co-Culture Model.

	Nuclear HMGB1	Cyto. HMGB1	Calreticulin	% Phago.	P. Index	P. Capacity
**ORR**	r = -0.380 p = 0. 279	r = 0.380 p = 0.279	**r = -0.713** **p = 0.021**	r = 0.475 p = 0.165	r = 0.510 p = 0.132	r = 0.289 p = 0.417
**Mean NADH Lifetime**	r = 0.483 p = 0.157	r = -0.483 p = 0.157	**r = 0.858** **p = 0.001**	r = -0.513 p = 0.129	r = -0.590 p = 0.072	r = -0.301 p = 0.399
**A1/A2 Ratio**	r = 0.214 p = 0.553	r = -0.214 p = 0.553	r = 0.537 p = 0.109	**r = -0.713** **p = 0.021**	r = -0.620 p = 0.056	r = 0.157 p = 0.664
**Ki67**	r = 0.534 p = 0.112	r = -0.534 p = 0.112	**r = 0.915** **p < 0.0001**	r = -0.625 p = 0.053	r = -0.606 p = 0.063	r = -0.108 p = 0.766
**CC3**	**r = 0.678** **p = 0.031**	**r = -0.678** **p = 0.031**	**r = 0.853** **p = 0.002**	r = -0.463 p = 0.178	r = -0.430 p = 0.2147	r = -0.131 p = 0.718
**HIF-1α**	r = 0.538 p = 0.109	r = -0.538 p = 0.109	**r = 0.879** **p = 0.001**	r = -0.531 p = 0.115	r = -0.523 p = 0.121	r = -0.086 p = 0.813
**HIF-2α**	r = -0.223 p = 0.535	r = 0.223 p = 0.535	r = -0.177 p = 0.625	r = 0.084 p = 0.817	r = 0.249 p = 0.488	r = 0.584 p = 0.077

Degrees of Correlation: High (r: ± 0.50 – ± 1.0), Moderate (r: ± 0.30 – ± 0.49), Low (r: < ± 0.29); Bolded values: Statistically significant

## 4 Discussion

Immunotherapy is a technique to enhance the immune system and its components (T-cells, macrophages, etc.) to target proliferating tumor cells while limiting negative systemic effects associated with chemotherapeutic approaches (4). Several clinically approved approaches (such as cancer vaccines, ACT therapies, and monoclonal antibodies) have gained clinical traction to treat CRC (5). More specifically, immune checkpoint inhibitors that target pathways such as CTLA-4 and PD-L1 have been shown to maintain immune system homeostasis through regulating immune responses (6, 9, 10). However, these treatments cannot be used to treat all cancer types, such as colorectal, pancreatic, prostate, and some breast cancers (14, 15). Recent studies have emphasized the importance of investigating heterogeneous cellular populations within a solid tumor and how they respond to immune checkpoint inhibitors (16). Utilization of ICD has been an emerging topic in the enhancement of tumor immunogenicity (19, 20). ICD can be induced through various stimuli such as chemotherapeutic regimens, radiotherapy, and other physiochemical therapies, which activate the immune system (21). Researchers have found specific hallmarks that can be used to determine whether ICD has occurred: ATP release and nuclear-to-cytoplasmic translocation of HMGB1 and nuclear-to-cytoplasmic translocation of calreticulin. Although chemotherapeutic agents such as FOLFOX and 5-FU have been shown to induce ICD, they increase the expression of immune checkpoints (such as PD-L1 and CD47), leading to chemotherapy resistance (23). Studies have shown that the *in vivo* blockade of an immune checkpoint (CD47) in conjunction with a chemotherapeutic agent increased survival and reduced tumor burden. Studying the interactions between macrophages and cancer cells is difficult due to the complexity of using an animal model and the lack of variable control (14, 32). Therefore, studying the cellular effects of a combinatory therapy regimen between macrophages and cancer cells to determine whether ICD has occurred should be explored. In this study, we used a multicellular spheroid model in conjunction with single spheroid imaging to understand the microregional structural and metabolic changes of a simulated solid tumor model, in addition to using conventional 2D co-culture monolayers to quantify characteristic features of ICD (HMGB1 and calreticulin) and changes in macrophage functional behavior, while validating and correlating immune responses after exposure to the combinatory regimen of immune checkpoint inhibitors and an ICD inducer.

First, changes in spheroid growth were investigated. After 24 hours, spheroids treated with 5-FU only or with combinatory treatments showed the greatest decrease in diameter, while spheroids treated with the immune checkpoint inhibitors showed only slight decreases in diameter. During this time, these two treatment regimens also showed the smallest distribution of spheroid diameter, meaning that these treatments may be targeting all spheroids during culture. After 48 hours, all treatments showed decreases in spheroid diameter, with the combination group showing the greatest decrease. Interestingly, after 48 hours, the combination group showed similar spheroid diameter distributions seen 24 hours post-treatment. 5-FU, anti-CD47, and anti-PD-L1-treated spheroids showed wide distributions of spheroid diameters. Overall, these results indicate that not only is the combination of an ICD inducer with two immune checkpoint inhibitors slow spheroid growth, but also shows a narrower distribution of spheroid diameters, meaning that these treatments could be targeting all spheroids in culture.

Next, structural changes in cellular expression across spheroid regions were explored. At the core, the addition of treatments showed that all structural markers showed an increase in normalized intensity over time. Also in the core, significant increases in M1 and M2 macrophage populations with no changes in immune checkpoint expression were observed. In the quiescent region, hypoxia increased over time, with increases in cellular apoptosis in all groups but the combination group. Also, a decrease in M2s and CD47 and SIRP-α expression with an increase in M1s was observed. It was also observed that all treatments except for the anti-CD47 group showed increases in PD-L1 expression, while PD-1 expression showed an initial increase in expression in all treatment groups except for the anti-CD47 group. At the edge, proliferation and apoptosis decreased over time, while acute and chronic hypoxia increased over time, regardless of treatment. At the edge, all macrophage populations showed a decrease over time with little to no changes in immune checkpoint expression. Based on these results, the combinatory treatment regimen may be showing that downstream cellular signaling responses in response to changes in spheroid structure are stronger than those of the other treatments with changes in cellular expression for both macrophage populations but no significant changes in immune checkpoint expression even with the presence of an immune checkpoint inhibitor as a monotherapy or in combination with chemotherapy.

Metabolic metrics were then explored for all treatment groups across spheroid regions. Regardless of treatment and length of treatment, the optical redox ratio increased across all spheroid microregions for all treatment groups across all time points. Overall, these results indicate that all treated spheroids could be showing a shift toward oxidative phosphorylation rather than glycolysis for metabolic demands. However, because the optical redox ratio shows less sensitivity to microenvironmental changes, additional fluorescence lifetime imaging (FLIM) metrics were used to investigate changes in NADH. Again, regardless of treatment and length of treatment, the mean NADH lifetime decreased across all spheroid microregions for all treatment groups across all time points. Mixed results were observed for A1/A2 ratios. At the core, 5-FU-treated spheroids saw no changes in the A1/A2 ratio, while the A1/A2 ratios within the anti-CD47 group increased over time. Anti-PD-L1-treated spheroids showed an initial decrease in the A1/A2 ratio after 24 hours before showing no additional changes after 48 hours. Spheroids in the combination group showed an initial decrease in the A1/A2 ratio after 24 hours before increasing after 48 hours. Similar trends were also observed in the quiescent region and at the edge. Based on the FLIM metrics, the decrease in mean NADH lifetime could indicate more free NADH rather than protein-bound NADH in the cytoplasm. Shorter mean NADH lifetimes generally indicate a reduced cellular redox state, meaning that the reduced form (NADH) is more readily oxidized, leading to a faster decay in fluorescence (36, 37). For A1/A2 ratios, an increase such as those observed in the anti-CD47 group at the spheroid edge could indicate a more glycolytic metabolism, while a decrease such as those observed in the anti-PD-L1 group could indicate a shift toward oxidative phosphorylation rather than glycolysis for metabolic demands. Increases in A1/A2 ratios indicate that there is a greater proportion of free NADH to protein-bound NADH, which could be indicative of increased glycolysis or impaired mitochondrial respiration, which could lead to a buildup of NADH. Future studies should investigate molecular changes within the electron transport chain to confirm any impairments in mitochondrial respiration within the spheroid model after treatment.

Using Pearson correlation matrices, numerous relationships were observed at different spheroid regions. At the core, it was found that as macrophage populations increase, cellular proliferation and the optical redox ratio also increase, while the mean NADH lifetime decreases. In the spheroid core, this region typically contains necrotic cells with low nutrients and oxygen concentrations and higher CO_2_ and lactate levels. Based on our results, the significant increase in acute hypoxia, M1 and M2 macrophages, along with cellular proliferation that’s observed in the combination group along with the increase in the optical redox ratio and decrease in mean NADH lifetime, could indicate that after treatment, a shift toward a microenvironment where cells have too little oxygen or are unable to use oxygen efficiently is observed compared to the microenvironment before treatment. In the quiescent region, several relationships were found. As M1 macrophages increase, M2 macrophages, along with SIRP-a expression and mean NADH lifetime, decrease while the optical redox ratio increases. It was also found that as cellular proliferation and apoptosis increase, PD-L1 expression decreases. The quiescent zone of a spheroid is characterized as a zone with viable and non-proliferative cells with low oxygen and nutrient levels. Based on our results, as chronic hypoxia increases within the combination group, cellular proliferation and apoptosis decrease over time, with the increase in optical redox ratio and decrease in CD47, SIRP-α, and PD-1. These results could indicate that this modality does show an effect on cellular proliferation and apoptosis compared to the other treatment groups where proliferation and apoptosis increase. At the edge, as macrophage populations increase, cellular proliferation and apoptosis increase, along with acute hypoxia and mean NADH lifetime. The spheroid edge contains highly proliferative cells with high oxygen and nutrient gradients. In the combination group, we see that M1 and M2 macrophages decrease, along with decreases in acute hypoxia, proliferation, and apoptosis, and a decrease in mean NADH lifetime. The increase in chronic hypoxia shows that oxygen demand is greater than supply, which can cause an increase in a more glycolytic cellular phenotype; however, since the optical redox ratio indicates a more oxidative metabolic demand, this could indicate that the spheroid edge of the combination group may show a more plastic metabolic profile, which could affect the distribution of cellular populations and cellular expression. More studies need to be performed to explore levels of ATP and lactate production to confirm whether this plastic metabolic profile exists.

In the spheroid model, it was observed that most of the significant changes occurred within the first 24 hours after treatment. To not only quantify changes in ICD hallmarks that could not be observed in the spheroid model but also validate changes in cellular responses at the spheroid edge, a 2D co-culture model of RAW 264.7/CT26 cells cultured under normoxic conditions was used. When comparing changes in M1 and M2 macrophages within the 2D model, it was found that cultures treated with anti-CD47 displayed the highest average number of cells expressing CD80 (M1 marker) compared to the other treatment groups; however, cultures in the combination group were the only cultures to show a decrease in M2 macrophages after 24 hours of treatment. These changes in macrophage populations may have some clinical implications that can influence cancer progression and therapeutic outcomes. Although we see a decrease in the M2s in the combination treatment, macrophage plasticity is still a concern in the clinical application of macrophage-targeted therapies. When looking at other cellular expression levels, it was observed that regardless of treatment, an increase in PD-L1 expression was observed; however, the combination group was able to show a decrease in CD47 expression with an increase in SIRP-α expression. Next, ICD hallmarks were explored. It was found that the addition of immune checkpoint inhibitors to a chemotherapeutic ICD inducer increased the nuclear translocation of HMGB1 and calreticulin release into the cytoplasm after 24 hours of treatment. It was also found that cultures in the combination group showed an increase in % phagocytosis of CT26 cells by over 5% after treatment, with an almost 3-fold change in phagocytosis index and a 1.2-fold change in phagocytosis capacity. The induction of ICD through chemotherapeutic regimens can elicit immunogenic responses through the induction of damage-associated molecular patterns (DAMPs) that can be recognized by immune cells such as macrophages (15). For example, calreticulin interacts with a variety of immune cell receptors such as CD91 that can stimulate phagocytosis of immune cells against tumor cells, while HMGB1 release can play an important role in the activation of antigen-presenting cells (38). Several studies have shown that chemotherapy can activate toll-like receptor 4 (TLR4) receptors on macrophages, further activating immune responses, while other studies have shown that chemotherapy drugs can remodel tumor cell immunogenicity through the regulation of major histocompatibility class I (MHC I) expression, enhancing tumor antigen presentation (39–41). These results indicate that the combinatory regimen does increase the phagocytic function and phagocytic capacity of macrophages. Future studies should be conducted to investigate changes in MHC I expression and other immune response receptors to determine the usefulness of the proposed treatments.

To correlate/validate changes in cellular responses observed after treatment, additional Pearson correlation matrices were performed. When comparing 3D optical metrics to the ICD hallmarks and changes in phagocytic function, several significant correlations were observed. As mean NADH lifetime, acute hypoxia, and cellular apoptosis and proliferation increases, calreticulin and nuclear HMGB1 expression increases, while cytoplasmic HMGB1 expression decreases. It was also found when comparing the optical redox ratio and A1/A2 that calreticulin decreases. In the combination group, it was observed that at the spheroid edge, proliferation, apoptosis, and acute hypoxia decreased after 24 hours of treatment. In the 2D co-culture model, calreticulin and cytoplasmic HMGB1 decreased, while nuclear HMGB1 expression and % phagocytosis increased. Based on the relationship, the decrease in mean NADH lifetime, acute hypoxia, and cellular proliferation and apoptosis mean that there could also be a decrease in calreticulin expression based on the 2D model. More studies should be conducted in measuring ICD hallmarks in the 3D culture model to determine if the relationship holds.

Overall, this study was able to quantify microregional metabolic and structural changes in a simulated RAW 264.7/CT26 spheroid model using single spheroid imaging, while using conventional 2D co-culture monolayers to quantify changes in ICD hallmarks and macrophage functional behavior. Based on these results, we also found a potential relationship between the changes in apoptosis at the spheroid edge versus changes in the translocation of HMGB1 through 2D monolayers after exposure to the combinatory regimen of immune checkpoint inhibitors and an ICD inducer. Even though these results indicate that this combinatory regimen seems to induce ICD, future studies should include investigating changes in ICD hallmarks within spheroid microregions rather than 2D monolayers to determine exact cellular behavior as well as investigating changes in ATP or metabolic intermediate levels to help determine detailed specifics on changes in metabolism after exposure to the treatments listed in this study.

## Data Availability

The raw data supporting the conclusions of this article will be made available by the authors, without undue reservation.
