# Supplementary material for: Quantifying treatment response to a macrophage-targeted therapy in combination with immune checkpoint inhibitors after exposure to conventional chemotherapy
Source: Front Immunol. 2025 Apr 28;16:1565953. doi: 10.3389/fimmu.2025.1565953 (PMC12066502; doi:10.3389/fimmu.2025.1565953)
Supplement: Supplementary file 1 [file DataSheet1.docx]

**Supplemental Information: Quantifying treatment response to a macrophage-targeted therapy in combination with an immune checkpoint inhibitor after exposure to conventional chemotherapy**

Shelby N. Bess^1^, Gaven K. Smart^1^, Timothy J. Muldoon^1*^

^1^Department of Biomedical Engineering, University of Arkansas, Fayetteville, AR

*Corresponding Author: tmuldoon@uark.edu


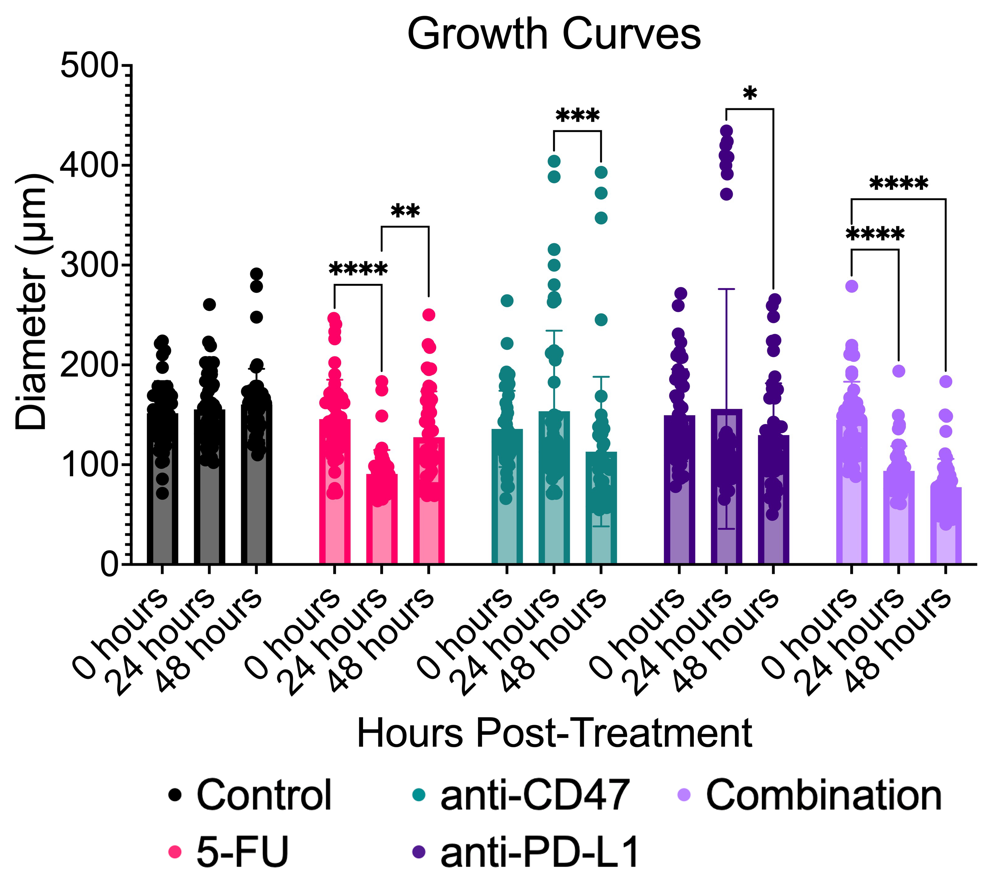


**Supplemental Figure 1. Significant changes in spheroid diameter (µm) were observed over time within each treatment group.**  . * p ≤ 0.05, ** p ≤ 0.01, *** p ≤ 0.001, **** p ≤ 0.0001. Plots were made in GraphPad Prism ®. Scale bars are 20 µm.


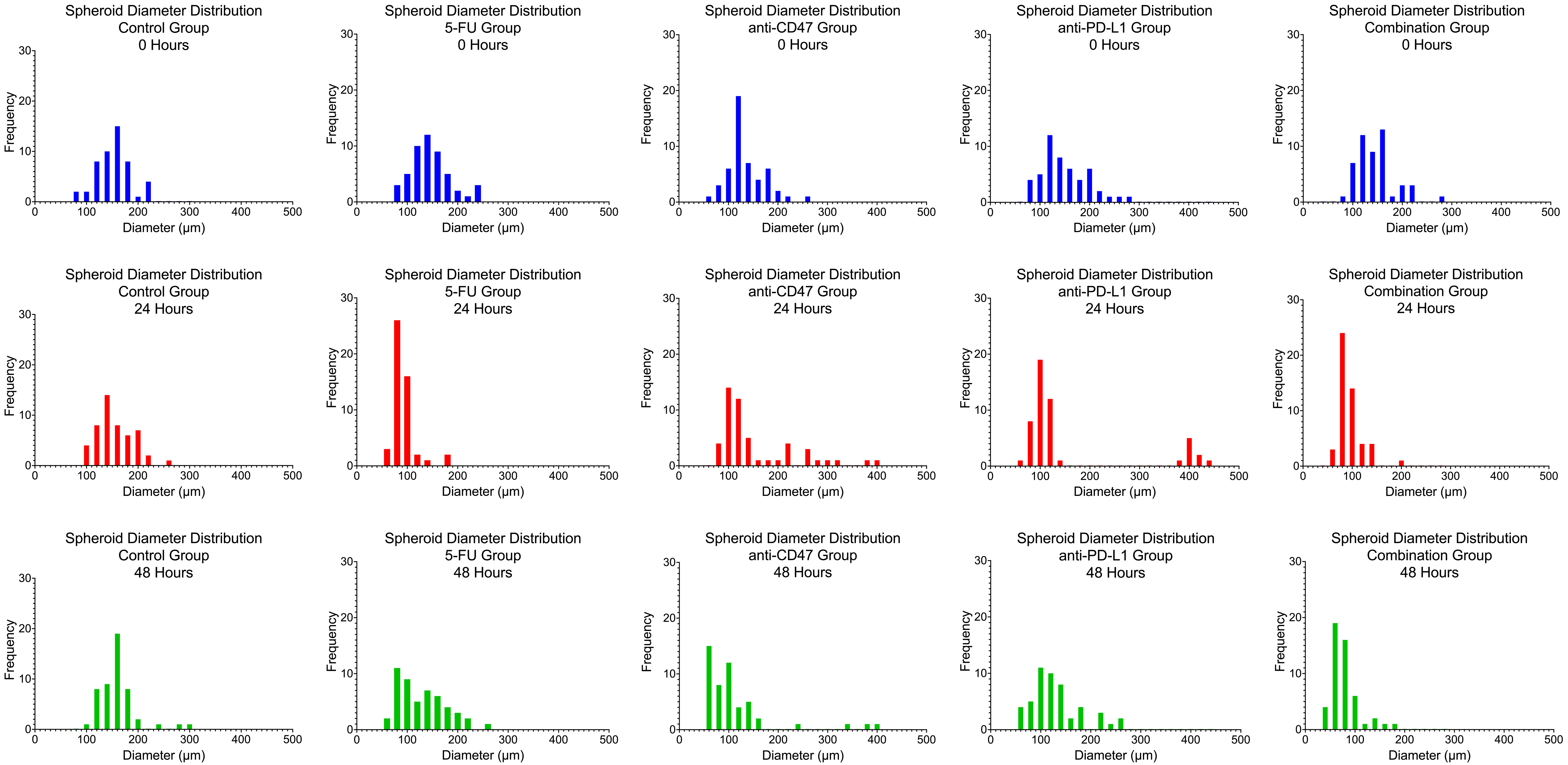


**Supplemental Figure 2. Frequency distributions of spheroid diameters before and after treatment.** Top: Frequency distributions before treatment. Middle: Frequency distributions 24 hours after treatment. Bottom: Frequency distributions 48 hours treatment. Plots were made in GraphPad Prism ®.


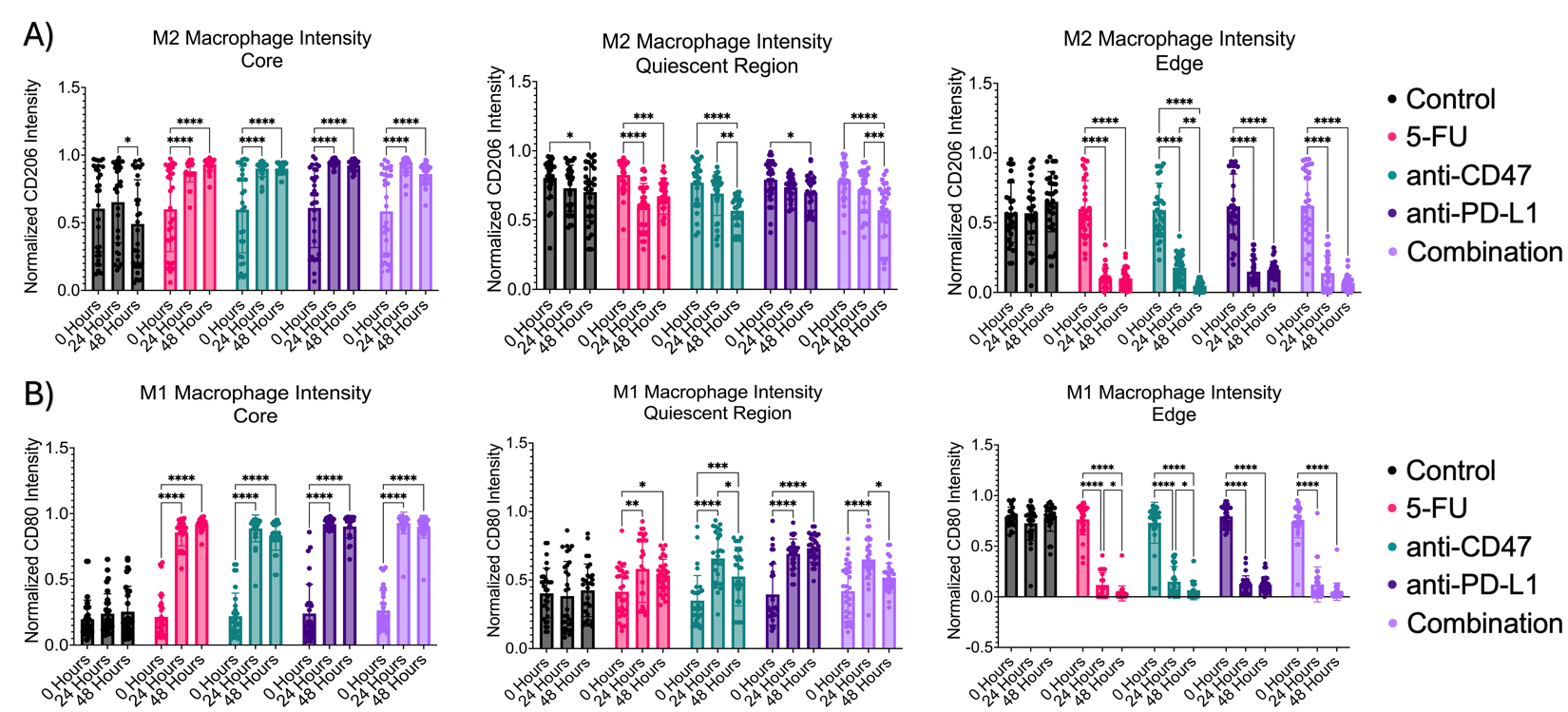


**Supplemental Figure 3. Macrophage expression across spheroid regions show significant changes within each treatment group.** Top: Normalized CD206 pixel intensities. Bottom: Normalized CD80 pixel intensities. * p ≤ 0.05, ** p ≤ 0.01, *** p ≤ 0.001, **** p ≤ 0.0001. Plots were made in GraphPad Prism ®.

**Supplemental Table 1. Summary of Normalized CD206 and CD80 Intensity Values**

| Timepoint | Group | Region | CD206 ± SD | p-value  (vs Control) | CD80 ± SD | p-value  (vs Control) |
| --- | --- | --- | --- | --- | --- | --- |
| 0 Hours | *Control* | Core | 0.604 ± 0.322 | - | 0.196 ± 0.146 | - |
|  |  | Quiescent | 0.805 ± 0.147 | - | 0.403 ± 0.183 | - |
|  |  | Edge | 0.573 ± 0.214 | - | 0.791 ± 0.095 | - |
|  | *5-FU* | Core | 0.600 ± 0.313 | > 0.9999 | 0.214 ± 0.166 | 0.9864 |
|  |  | Quiescent | 0.825 ± 0.114 | 0.9843 | 0.415 ± 0.179 | 0.9993 |
|  |  | Edge | 0.598 ± 0.204 | 0.9756 | 0.765 ± 0.152 | 0.9365 |
|  | *anti-CD47* | Core | 0.597 ± 0.319 | > 0.9999 | 0.220 ± 0.175 | 0..9589 |
|  |  | Quiescent | 0.769 ± 0.169 | 0.8870 | 0.350 ± 0.183 | 0.7907 |
|  |  | Edge | 0.590 ± 0.193 | 0.9933 | 0.731 ± 0.203 | 0.3917 |
|  | *anti-PD-L1* | Core | 0.610 ± 0.294 | > 0.9999 | 0.240 ± 0.222 | 0.7177 |
|  |  | Quiescent | 0.791 ± 0.146 | 0.9964 | 0.395 ± 0.224 | 0.9998 |
|  |  | Edge | 0.617 ± 0.232 | 0.8239 | 0.793 ± 0.099 | >0.9999 |
|  | *Combination* | Core | 0.584 ± 0.308 | > 0.9999 | 0.263 ± 0.150 | 0.2978 |
|  |  | Quiescent | 0.786 ± 0.131 | 0.9880 | 0.419 ± 0.198 | 0.9972 |
|  |  | Edge | 0.622 ± 0.250 | 0.7562 | 0.742 ± 0.159 | 0.5923 |
| 24 Hours | *Control* | Core | 0.652 ± 0.301 | - | 0.238 ± 0.151 | - |
|  |  | Quiescent | 0.729 ± 0.167 | - | 0.383 ± 0.253 | - |
|  |  | Edge | 0.568 ± 0.226 | - | 0.726 ± 0.170 | - |
|  | *5-FU* | Core | 0.880 ± 0.078 | ***0.0005*** | 0.857 ± 0.102 | ***<0.0001*** |
|  |  | Quiescent | 0.593 ± 0.171 | ***0.0042*** | 0.581 ± 0.245 | ***0.0003*** |
|  |  | Edge | 0.102 ± 0.072 | ***<0.0001*** | 0.117 ± 0.123 | ***<0.0001*** |
|  | *anti-CD47* | Core | 0.900 ± 0.060 | ***0.0001*** | 0.887 ± 0.102 | ***<0.0001*** |
|  |  | Quiescent | 0.691 ± 0.157 | 0.8556 | 0.658 ± 0.185 | ***<0.0001*** |
|  |  | Edge | 0.178 ± 0.096 | ***<0.0001*** | 0.149 ± 0.150 | ***<0.0001*** |
|  | *anti-PD-L1* | Core | 0.943 ± 0.028 | ***<0.0001*** | 0.920 ± 0.038 | ***<0.0001*** |
|  |  | Quiescent | 0.737 ± 0.082 | 0.9997 | 0.691 ± 0.109 | ***<0.0001*** |
|  |  | Edge | 0.149 ± 0.087 | ***<0.0001*** | 0.125 ± 0.082 | ***<0.0001*** |
|  | *Combination* | Core | 0.918 ± 0.067 | ***<0.0001*** | 0.929 ± 0.082 | ***<0.0001*** |
|  |  | Quiescent | 0.719 ± 0.147 | 0.9989 | 0.651 ± 0.144 | ***<0.0001*** |
|  |  | Edge | 0.138 ± 0.125 | ***<0.0001*** | 0.122 ± 0.173 | ***<0.0001*** |
| 48 Hours | *Control* | Core | 0.491 ± 0.327 | - | 0.254 ± 0.193 | - |
|  |  | Quiescent | 0.702 ± 0.206 | - | 0.425 ± 0.192 | - |
|  |  | Edge | 0.650 ± 0.215 | - | 0.785 ± 0.137 | - |
|  | *5-FU* | Core | 0.933 ± 0.045 | ***<0.0001*** | 0.916 ± 0.041 | ***<0.0001*** |
|  |  | Quiescent | 0.672 ± 0.130 | 0.9387 | 0.534 ± 0.117 | 0.1399 |
|  |  | Edge | 0.100 ± 0.072 | ***<0.0001*** | 0.033 ± 0.074 | ***<0.0001*** |
|  | *anti-CD47* | Core | 0.900 ± 0.041 | ***<0.0001*** | 0.828 ± 0.107 | ***<0.0001*** |
|  |  | Quiescent | 0.567 ± 0.113 | ***0.0050*** | 0.526 ± 0.215 | 0.2068 |
|  |  | Edge | 0.047 ± 0.037 | ***<0.0001*** | 0.065 ± 0.094 | ***<0.0001*** |
|  | *anti-PD-L1* | Core | 0.924 ± 0.039 | ***<0.0001*** | 0.901 ± 0.085 | ***<0.0001*** |
|  |  | Quiescent | 0.699 ± 0.128 | >0.9999 | 0.732 ± 0.100 | ***<0.0001*** |
|  |  | Edge | 0.160 ± 0.062 | ***<0.0001*** | 0.120 ± 0.079 | ***<0.0001*** |
|  | *Combination* | Core | 0.859 ± 0.075 | ***<0.0001*** | 0.900 ± 0.086 | ***<0.0001*** |
|  |  | Quiescent | 0.572 ± 0.192 | ***0.0077*** | 0.518 ± 0.106 | 0.2772 |
|  |  | Edge | 0.068 ± 0.053 | ***<0.0001*** | 0.050 ± 0.086 | ***<0.0001*** |


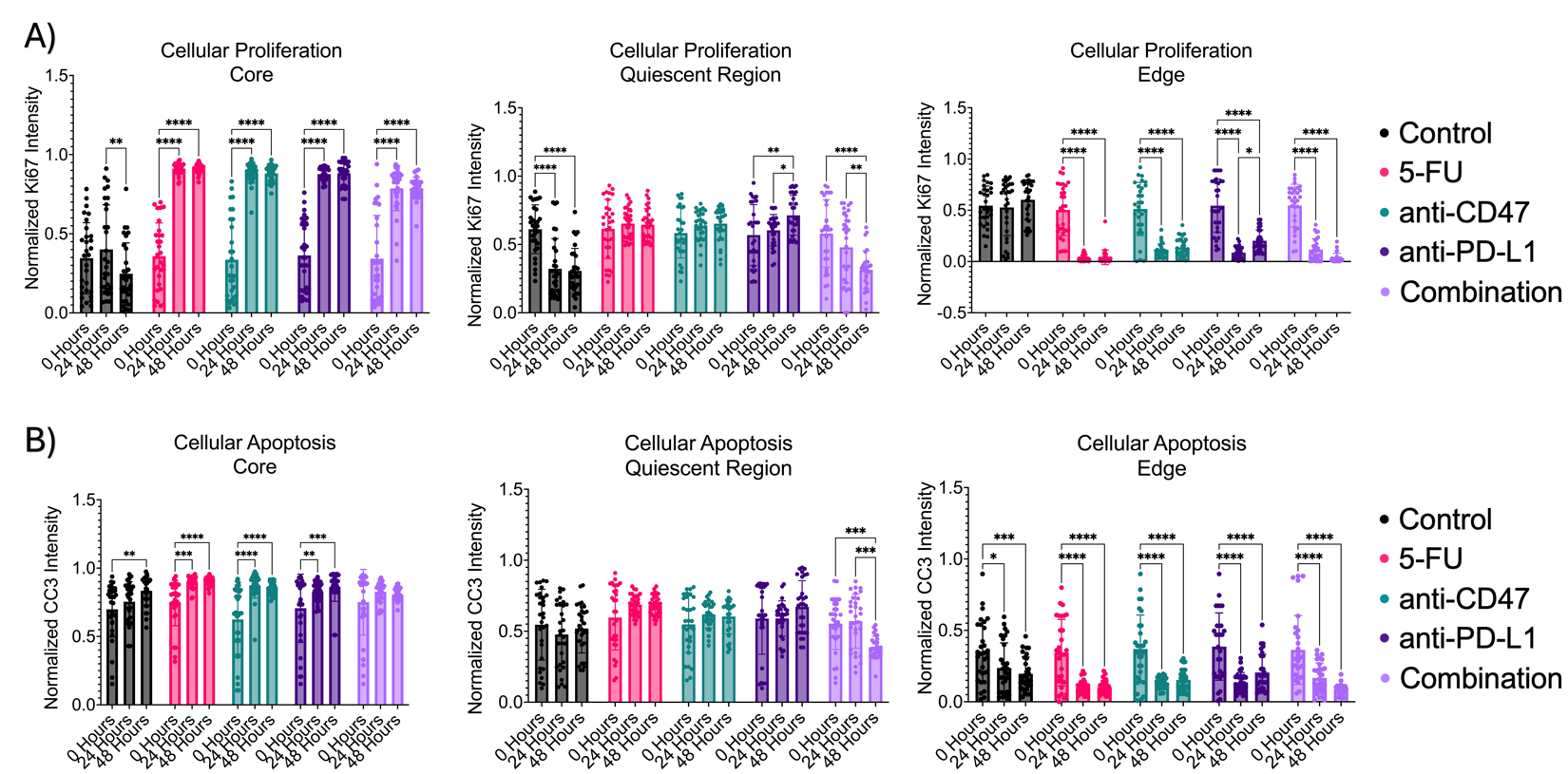


**Supplemental Figure 4. Significant changes were observed across spheroid regions between cellular proliferation and apoptosis within each treatment group.** Top: Normalized Ki67 pixel intensities. Bottom: Normalized CC3 pixel intensities. * p ≤ 0.05, ** p ≤ 0.01, *** p ≤ 0.001, **** p ≤ 0.0001. Plots were made in GraphPad Prism ®.

**Supplemental Table 2. Summary of Normalized Ki67 and CC3 Intensity Values**

| Timepoint | Group | Region | Ki67 ± SD | p-value  (vs Control) | CC3 ± SD | p-value  (vs Control) |
| --- | --- | --- | --- | --- | --- | --- |
| 0 Hours | *Control* | Core | 0.346 ± 0.224 | - | 0.699 ± 0.203 | - |
|  |  | Quiescent | 0.612 ± 0.178 | - | 0.546 ± 0.250 | - |
|  |  | Edge | 0.545 ± 0.191 | - | 0.351 ± 0.216 | - |
|  | *5-FU* | Core | 0.358 ± 0.210 | 0.9986 | 0.744 ± 0.166 | 0.7496 |
|  |  | Quiescent | 0.613 ± 0.216 | >0.9999 | 0.597 ± 0.228 | 0.7942 |
|  |  | Edge | 0.612 ± 0.178 | 0.8757 | 0.344 ± 0.233 | 0.9998 |
|  | *anti-CD47* | Core | 0.502 ± 0.251 | 0.9995 | 0.624 ± 0.253 | 0.2799 |
|  |  | Quiescent | 0.583 ± 0.183 | 0.9717 | 0.548 ± 0.200 | >0.9999 |
|  |  | Edge | 0.512 ± 0.260 | 0.9488 | 0.370 ± 0.238 | 0.9904 |
|  | *anti-PD-L1* | Core | 0.363 ± 0.213 | 0.9950 | 0.707 ± 0.249 | 0.9996 |
|  |  | Quiescent | 0.568 ± 0.223 | 0.8810 | 0.589 ± 0.251 | 0.8727 |
|  |  | Edge | 0.545 ± 0.260 | >0.9999 | 0.386 ± 0.236 | 0.9014 |
|  | *Combination* | Core | 0.342 ± 0.273 | >0.9999 | 0.612 ± 0.178 | 0.6475 |
|  |  | Quiescent | 0.580 ± 0.247 | 0.9571 | 0.554 ± 0.182 | 0.9998 |
|  |  | Edge | 0.545 ± 0.208 | >0.9999 | 0.363 ± 0.241 | 0.9980 |
| 24 Hours | *Control* | Core | 0.401 ± 0.283 | - | 0.751 ± 0.240 | - |
|  |  | Quiescent | 0.322 ± 0.221 | - | 0.477 ± 0.225 | - |
|  |  | Edge | 0.527 ± 0.275 | - | 0.237 ± 0.176 | - |
|  | *5-FU* | Core | 0.912 ± 0.035 | ***<0.0001*** | 0.902 ± 0.042 | ***0.0010*** |
|  |  | Quiescent | 0.653 ± 0.111 | ***<0.0001*** | 0.687 ± 0.072 | ***<0.0001*** |
|  |  | Edge | 0.033 ± 0.033 | ***<0.0001*** | 0.101 ± 0.046 | ***0.0067*** |
|  | *anti-CD47* | Core | 0.886 ± 0.064 | ***<0.0001*** | 0.873 ± 0.092 | ***0.0148*** |
|  |  | Quiescent | 0.636 ± 0.109 | ***<0.0001*** | 0.619 ± 0.095 | ***0.0155*** |
|  |  | Edge | 0.108 ± 0.063 | ***<0.0001*** | 0.133 ± 0.039 | 0.0732 |
|  | *anti-PD-L1* | Core | 0.877 ± 0.041 | ***<0.0001*** | 0.826 ± 0.071 | 0.3173 |
|  |  | Quiescent | 0.604 ± 0.115 | ***<0.0001*** | 0.590 ± 0.124 | 0.0917 |
|  |  | Edge | 0.086 ± 0.058 | ***<0.0001*** | 0.136 ± 0.069 | 0.0874 |
|  | *Combination* | Core | 0.787 ± 0.141 | ***<0.0001*** | 0.826 ± 0.073 | 0.3126 |
|  |  | Quiescent | 0.479 ± 0.261 | ***0.0062*** | 0.571 ± 0.191 | 0.2304 |
|  |  | Edge | 0.116 ± 0.107 | ***<0.0001*** | 0.167 ± 0.105 | 0.4112 |
| 48 Hours | *Control* | Core | 0.248 ± 0.195 | - | 0.834 ± 0.106 | - |
|  |  | Quiescent | 0.306 ± 0.165 | - | 0.519 ± 0.172 | - |
|  |  | Edge | 0.602 ± 0.195 | - | 0.196 ± 0.117 | - |
|  | *5-FU* | Core | 0.912 ± 0.029 | ***<0.0001*** | 0.905 ± 0.031 | 0.3311 |
|  |  | Quiescent | 0.644 ± 0.118 | ***<0.0001*** | 0.683 ± 0.070 | ***0.0030*** |
|  |  | Edge | 0.041 ± 0.072 | ***<0.0001*** | 0.101 ± 0.046 | 0.1296 |
|  | *anti-CD47* | Core | 0.881 ± 0.050 | ***<0.0001*** | 0.853 ± 0.046 | 0.9869 |
|  |  | Quiescent | 0.651 ± 0.122 | ***<0.0001*** | 0.604 ± 0.122 | 0.3353 |
|  |  | Edge | 0.137 ± 0.091 | ***<0.0001*** | 0.153 ± 0.075 | 0.8310 |
|  | *anti-PD-L1* | Core | 0.882 ± 0.076 | ***<0.0001*** | 0.863 ± 0.105 | 0.9451 |
|  |  | Quiescent | 0.713 ± 0.145 | ***<0.0001*** | 0.672 ± 0.184 | ***0.0070*** |
|  |  | Edge | 0.203 ± 0.106 | ***<0.0001*** | 0.205 ± 0.126 | 0.9993 |
|  | *Combination* | Core | 0.787 ± 0.069 | ***<0.0001*** | 0.807 ± 0.044 | 0.9508 |
|  |  | Quiescent | 0.314 ± 0.140 | 0.9998 | 0.387 ± 0.078 | ***0.0288*** |
|  |  | Edge | 0.033 ± 0.046 | ***<0.0001*** | 0.085 ± 0.037 | ***0.0465*** |


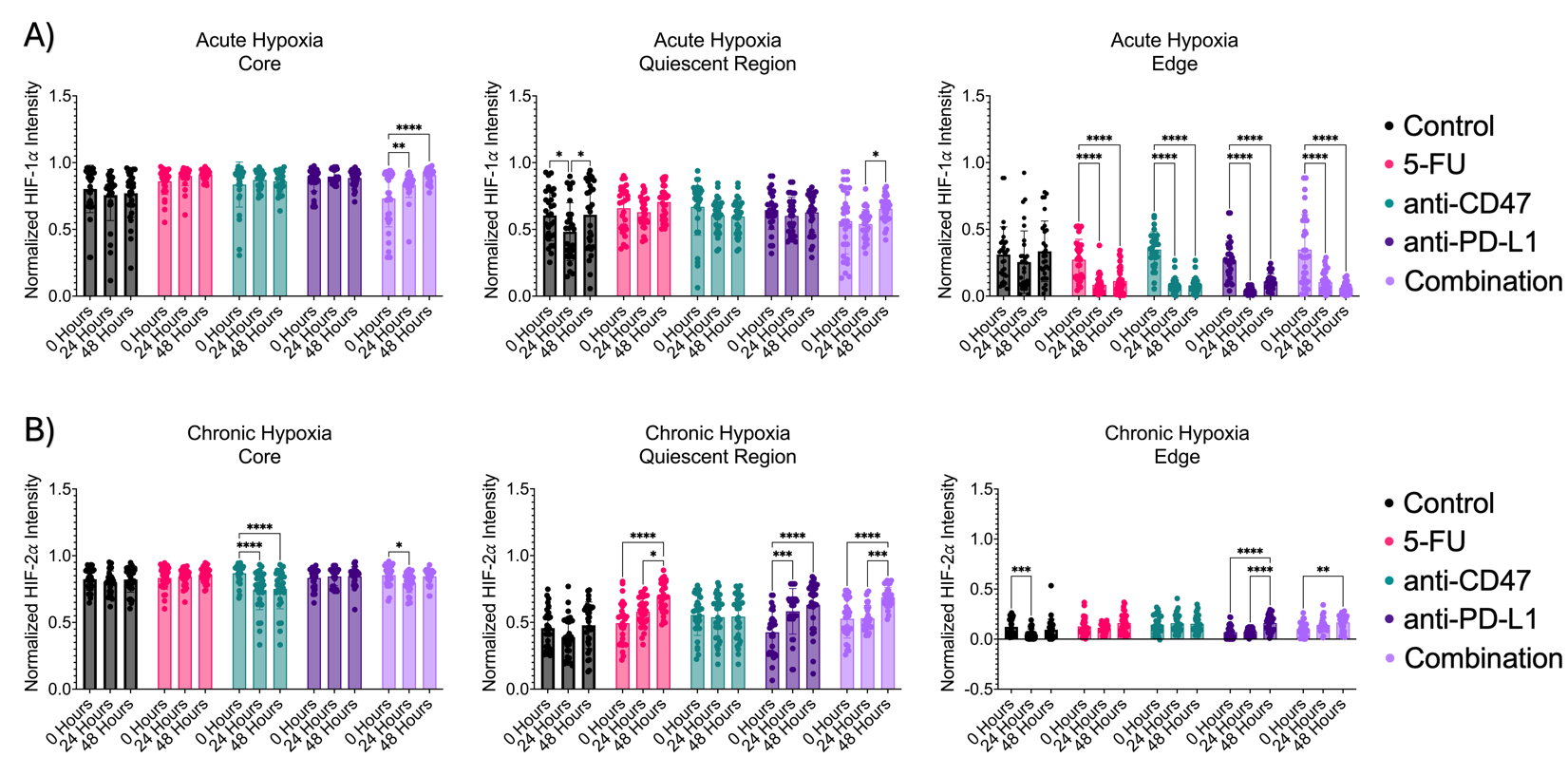


**Supplemental Figure 5. Significant changes were observed across spheroid regions between acute and chronic hypoxia within each treatment group.** Top: Normalized HIF-1𝛼 pixel intensities. Bottom: Normalized HIF-2𝛼 pixel intensities. * p ≤ 0.05, ** p ≤ 0.01, *** p ≤ 0.001, **** p ≤ 0.0001. Plots were made in GraphPad Prism ®.

**Supplemental Table 3. Summary of Normalized HIF-1𝛼 and HIF-2𝛼 Intensity Values**

| Timepoint | Group | Region | HIF-1𝛼 ± SD | p-value  (vs Control) | HIF-2𝛼 ± SD | p-value  (vs Control) |
| --- | --- | --- | --- | --- | --- | --- |
| 0 Hours | *Control* | Core | 0.803 ± 0.177 | - | 0.824 ± 0.082 | - |
|  |  | Quiescent | 0.605 ± 0.189 | - | 0.457 ± 0.146 | - |
|  |  | Edge | 0.311 ± 0.207 | - | 0.123 ± 0.084 | - |
|  | *5-FU* | Core | 0.859 ± 0.098 | 0.3783 | 0.833 ± 0.085 | 0.9932 |
|  |  | Quiescent | 0.658 ± 0.165 | 0.7316 | 0.494 ± 0.154 | 0.8623 |
|  |  | Edge | 0.273 ± 0.153 | 0.8407 | 0.127 ± 0.089 | 0.9997 |
|  | *anti-CD47* | Core | 0.836 ± 0.168 | 0.8344 | 0.869 ± 0.070 | 0.2784 |
|  |  | Quiescent | 0.669 ± 0.212 | 0.5703 | 0.556 ± 0.153 | 0.0735 |
|  |  | Edge | 0.349 ± 0.133 | 0.8515 | 0.145 ± 0.092 | 0.8102 |
|  | *anti-PD-L1* | Core | 0.870 ± 0.084 | 0.2074 | 0.834 ± 0.076 | 0.9912 |
|  |  | Quiescent | 0.645 ± 0.144 | 0.8905 | 0.426 ± 0.162 | 0.9250 |
|  |  | Edge | 0.266 ± 0.145 | 0.7507 | 0.069 ± 0.065 | 0.0531 |
|  | *Combination* | Core | 0.732 ± 0.213 | 0.1645 | 0.854 ± 0.077 | 0.6646 |
|  |  | Quiescent | 0.562 ± 0.245 | 0.8587 | 0.527 ± 0.145 | 0.3425 |
|  |  | Edge | 0.348 ± 0.262 | 0.8560 | 0.102 ± 0.065 | 0.8195 |
| 24 Hours | *Control* | Core | 0.757 ± 0.191 | - | 0.799 ± 0.087 | - |
|  |  | Quiescent | 0.483 ± 0.217 | - | 0.389 ± 0.148 | - |
|  |  | Edge | 0.255 ± 0.233 | - | 0.047 ± 0.054 | - |
|  | *5-FU* | Core | 0.900 ± 0.072 | ***<0.0001*** | 0.841 ± 0.064 | 0.3492 |
|  |  | Quiescent | 0.629 ± 0.112 | ***0.0075*** | 0.579 ± 0.109 | ***< 0.0001*** |
|  |  | Edge | 0.085 ± 0.073 | ***< 0.0001*** | 0.113 ± 0.046 | ***0.0092*** |
|  | *anti-CD47* | Core | 0.869 ± 0.068 | ***0.0038*** | 0.743 ± 0.147 | 0.1029 |
|  |  | Quiescent | 0.607 ± 0.121 | ***0.0362*** | 0.539 ± 0.165 | ***0.0010*** |
|  |  | Edge | 0.082 ± 0.055 | ***< 0.0001*** | 0.159 ± 0.089 | ***< 0.0001*** |
|  | *anti-PD-L1* | Core | 0.896 ± 0.049 | ***0.0001*** | 0.844 ± 0.069 | 0.2748 |
|  |  | Quiescent | 0.601 ± 0.140 | 0.0530 | 0.583 ± 0.171 | ***< 0.0001*** |
|  |  | Edge | 0.040 ± 0.026 | ***< 0.0001*** | 0.075 ± 0.036 | 0.6354 |
|  | *Combination* | Core | 0.833 ± 0.094 | 0.1161 | 0.795 ± 0.072 | 0.9998 |
|  |  | Quiescent | 0.540 ± 0.110 | 0.6752 | 0.531 ± 0.097 | ***0.0021*** |
|  |  | Edge | 0.104 ± 0.079 | ***0.0006*** | 0.141 ± 0.070 | ***< 0.0001*** |
| 48 Hours | *Control* | Core | 0.771 ± 0.178 | - | 0.822 ± 0.095 | - |
|  |  | Quiescent | 0.610 ± 0.256 | - | 0.478 ± 0.174 | - |
|  |  | Edge | 0.335 ± 0.227 | - | 0.094 ± 0.177 | - |
|  | *5-FU* | Core | 0.911 ± 0.037 | ***0.0001*** | 0.858 ± 0.055 | 0.5206 |
|  |  | Quiescent | 0.705 ± 0.122 | 0.1856 | 0.689 ± 0.113 | ***< 0.0001*** |
|  |  | Edge | 0.113 ± 0.098 | ***< 0.0001*** | 0.160 ± 0.098 | **0.0099** |
|  | *anti-CD47* | Core | 0.860 ± 0.079 | ***0.0403*** | 0.751 ± 0.149 | ***0.0170*** |
|  |  | Quiescent | 0.597 ± 0.129 | 0.9982 | 0.543 ± 0.168 | 0.4344 |
|  |  | Edge | 0.081 ± 0.057 | ***< 0.0001*** | 0.154 ± 0.084 | ***0.0256*** |
|  | *anti-PD-L1* | Core | 0.884 ± 0.062 | ***0.0033*** | 0.845 ± 0.073 | 0.8438 |
|  |  | Quiescent | 0.628 ± 0.151 | 0.9942 | 0.631 ± 0.185 | ***0.0007*** |
|  |  | Edge | 0.110 ± 0.058 | ***< 0.0001*** | 0.161 ± 0.077 | ***0.0089*** |
|  | *Combination* | Core | 0.906 ± 0.050 | ***0.0002*** | 0.845 ± 0.058 | 0.8424 |
|  |  | Quiescent | 0.654 ± 0.096 | 0.8482 | 0.688 ± 0.074 | ***< 0.0001*** |
|  |  | Edge | 0.059 ± 0.044 | ***< 0.0001*** | 0.167 ± 0.077 | ***0.0034*** |


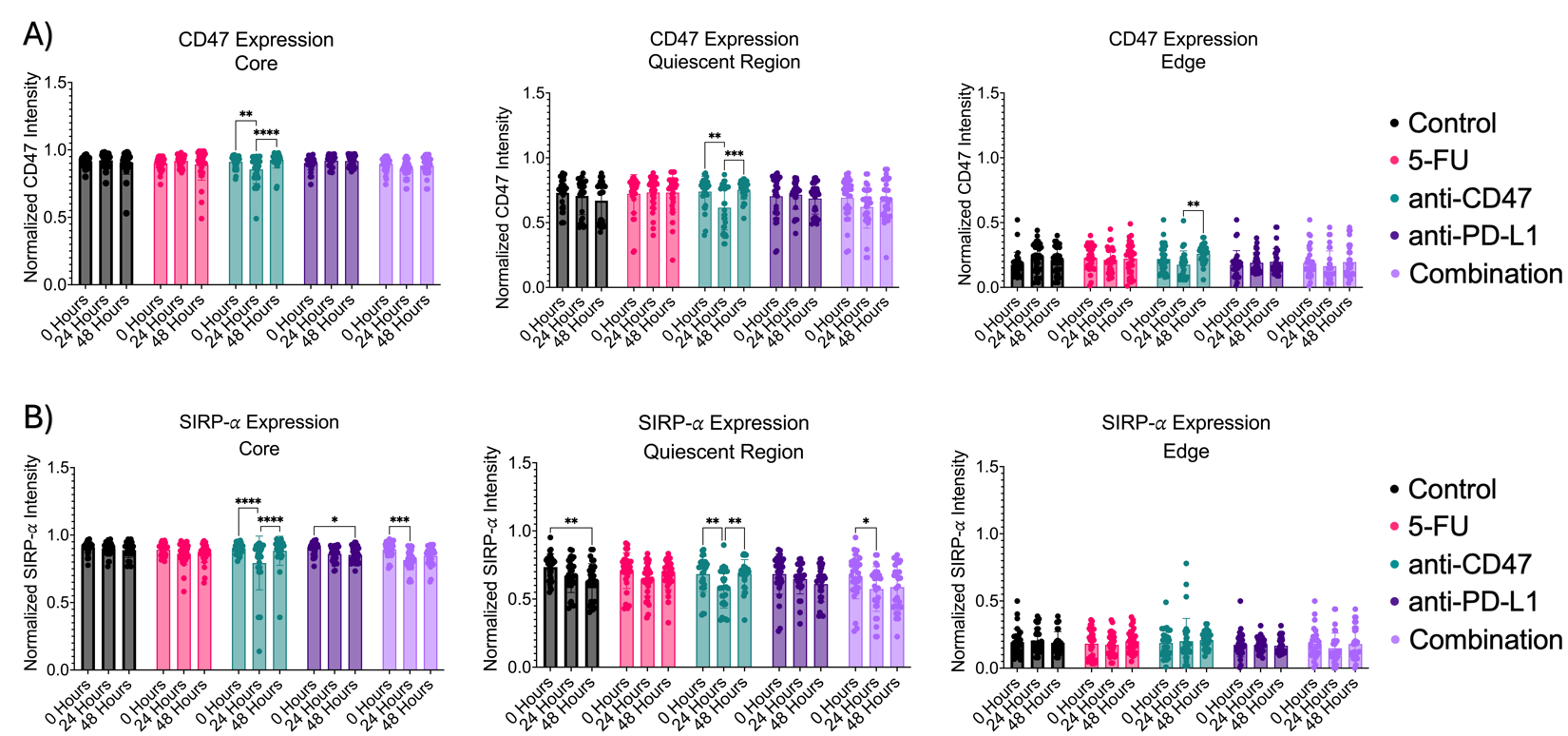


**Supplemental Figure 6. Significant changes were observed across spheroid regions between CD47 and SIRP-𝛼 expression within each treatment group.** Top: Normalized CD47 pixel intensities. Bottom: Normalized SIRP-𝛼 pixel intensities. * p ≤ 0.05, ** p ≤ 0.01, *** p ≤ 0.001, **** p ≤ 0.0001. Plots were made in GraphPad Prism ®.

**Supplemental Table 4. Summary of Normalized CD47 and SIRP-𝛼 Intensity Values**

| Timepoint | Group | Region | CD47 ± SD | p-value  (vs Control) | SIRP-𝛼 ± SD | p-value  (vs Control) |
| --- | --- | --- | --- | --- | --- | --- |
| 0 Hours | *Control* | Core | 0.913 ± 0.036 | - | 0.910 ± 0.043 | - |
|  |  | Quiescent | 0.729 ± 0.117 | - | 0.735 ± 0.096 | - |
|  |  | Edge | 0.175 ± 0.096 | - | 0.193 ± 0.100 | - |
|  | *5-FU* | Core | 0.899 ± 0.051 | 0.9283 | 0.890 ± 0.045 | 0.8709 |
|  |  | Quiescent | 0.723 ± 0.147 | 0.9999 | 0.709 ± 0.131 | 0.9424 |
|  |  | Edge | 0.228 ± 0.096 | 0.2770 | 0.182 ± 0.105 | 0.9936 |
|  | *anti-CD47* | Core | 0.911 ± 0.046 | > 0.9999 | 0.900 ± 0.041 | 0.9879 |
|  |  | Quiescent | 0.739 ± 0.116 | 0.9987 | 0.683 ± 0.131 | 0.5630 |
|  |  | Edge | 0.220 ± 0.108 | 0.4516 | 0.188 ± 0.101 | 0.9996 |
|  | *anti-PD-L1* | Core | 0.903 ± 0.051 | 0.9813 | 0.903 ± 0.046 | 0.9960 |
|  |  | Quiescent | 0.703 ± 0.168 | 0.9535 | 0.684 ± 0.148 | 0.5710 |
|  |  | Edge | 0.175 ± 0.109 | > 0.9999 | 0.174 ± 0.091 | 0.9519 |
|  | *Combination* | Core | 0.896 ± 0.053 | 0.8478 | 0.893 ± 0.056 | 0.9152 |
|  |  | Quiescent | 0.692 ± 0.162 | 0.8483 | 0.664 ± 0.162 | 0.2398 |
|  |  | Edge | 0.187 ± 0.116 | 0.9919 | 0.182 ± 0.108 | 0.9942 |
| 24 Hours | *Control* | Core | 0.923 ± 0.049 | - | 0.900 ± 0.049 | - |
|  |  | Quiescent | 0.705 ± 0.144 | - | 0.669 ± 0.122 | - |
|  |  | Edge | 0.230 ± 0.108 | - | 0.206 ± 0.088 | - |
|  | *5-FU* | Core | 0.918 ± 0.042 | 0.9987 | 0.861 ± 0.080 | 0.3284 |
|  |  | Quiescent | 0.732 ± 0.124 | 0.9469 | 0.641 ± 0.121 | 0.9277 |
|  |  | Edge | 0.213 ± 0.097 | 0.9679 | 0.174 ± 0.084 | 0.7064 |
|  | *anti-CD47* | Core | 0.855 ± 0.103 | ***0.0005*** | 0.793 ± 0.199 | ***<0.0001*** |
|  |  | Quiescent | 0.615 ± 0.172 | 0.0991 | 0.576 ± 0.142 | 0.0522 |
|  |  | Edge | 0.175 ± 0.106 | 0.2532 | 0.201 ± 0.169 | 0.9994 |
|  | *anti-PD-L1* | Core | 0.920 ± 0.041 | 0.9997 | 0.860 ± 0.056 | 0.2796 |
|  |  | Quiescent | 0.715 ± 0.111 | 0.9989 | 0.647 ± 0.110 | 0.9692 |
|  |  | Edge | 0.190 ± 0.078 | 0.5733 | 0.173 ± 0.055 | 0.6780 |
|  | *Combination* | Core | 0.859 ± 0.064 | ***0.0016*** | 0.815 ± 0.074 | ***0.0004*** |
|  |  | Quiescent | 0.621 ± 0.164 | 0.1429 | 0.574 ± 0.164 | ***0.0456*** |
|  |  | Edge | 0.164 ± 0.117 | 0.1017 | 0.148 ± 0.113 | 0.1489 |
| 48 Hours | *Control* | Core | 0.906 ± 0.087 | **-** | 0.889 ± 0.057 | - |
|  |  | Quiescent | 0.669 ± 0.158 | - | 0.626 ± 0.134 | - |
|  |  | Edge | 0.211 ± 0.103 | - | 0.189 ± 0.082 | - |
|  | *5-FU* | Core | 0.891 ± 0.116 | 0.9009 | 0.870 ± 0.072 | 0.8992 |
|  |  | Quiescent | 0.731 ± 0.150 | 0.4238 | 0.678 ± 0.108 | 0.5603 |
|  |  | Edge | 0.222 ± 0.121 | 0.9944 | 0.201 ± 0.086 | 0.9903 |
|  | *anti-CD47* | Core | 0.926 ± 0.061 | 0.7518 | 0.884 ± 0.107 | 0.9992 |
|  |  | Quiescent | 0.752 ± 0.071 | 0.1500 | 0.685 ± 0.104 | 0.4272 |
|  |  | Edge | 0.259 ± 0.072 | 0.3854 | 0.209 ± 0.066 | 0.9291 |
|  | *anti-PD-L1* | Core | 0.917 ± 0.042 | 0.9676 | 0.853 ± 0.056 | 0.4113 |
|  |  | Quiescent | 0.685 ± 0.120 | 0.9915 | 0.610 ± 0.131 | 0.9910 |
|  |  | Edge | 0.198 ± 0.101 | 0.9882 | 0.167 ± 0.058 | 0.9118 |
|  | *Combination* | Core | 0.884 ± 0.069 | 0.6584 | 0.846 ± 0.071 | 0.2233 |
|  |  | Quiescent | 0.666 ± 0.153 | > 0.9999 | 0.588 ± 0.168 | 0.8014 |
|  |  | Edge | 0.196 ± 0.124 | 0.9797 | 0.180 ± 0.118 | 0.9968 |


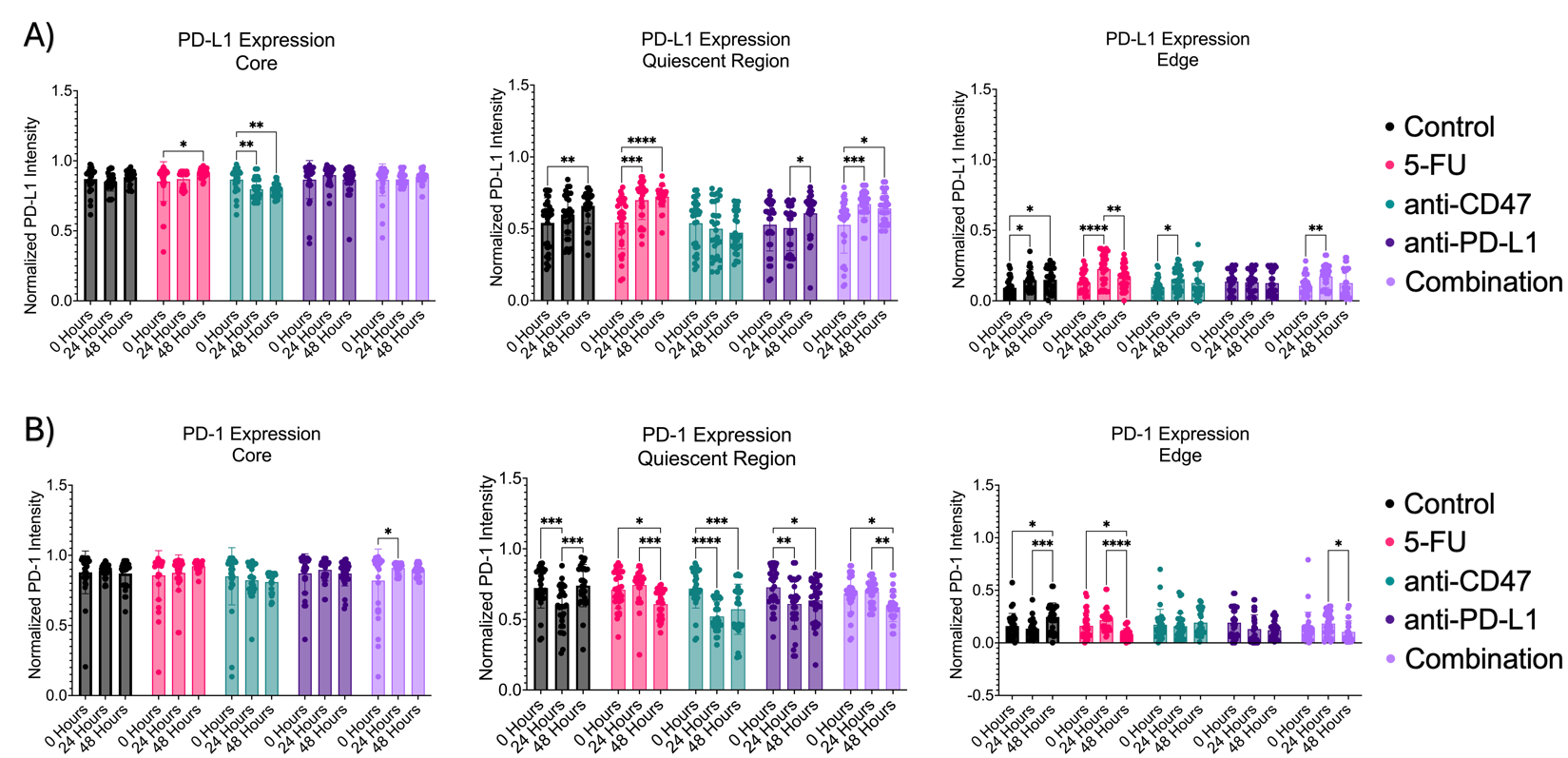


**Supplemental Figure 7. Significant changes were observed across spheroid regions between PD-1 and PD-L1 expression within each treatment group.** Top: Normalized PD-L1 pixel intensities. Bottom: Normalized PD-1 pixel intensities. * p ≤ 0.05, ** p ≤ 0.01, *** p ≤ 0.001, **** p ≤ 0.0001. Plots were made in GraphPad Prism ®.

**Supplemental Table 5. Summary of Normalized PD-L1 and PD-1 Intensity Values**

| Timepoint | Group | Region | PD-L1 ± SD | p-value  (vs Control) | PD-1 ± SD | p-value  (vs Control) |
| --- | --- | --- | --- | --- | --- | --- |
| 0 Hours | *Control* | Core | 0.870 ± 0.086 | - | 0.879 ± 0.152 | - |
|  |  | Quiescent | 0.541 ± 0.175 | - | 0.722 ± 0.142 | - |
|  |  | Edge | 0.092 ± 0.064 | - | 0.161 ± 0.119 | - |
|  | *5-FU* | Core | 0.852 ± 0.142 | 0.9129 | 0.857 ± 0.176 | 0.9629 |
|  |  | Quiescent | 0.543 ± 0.184 | >0.9999 | 0.710 ± 0.133 | 0.9970 |
|  |  | Edge | 0.130 ± 0.069 | 0.3793 | 0.162 ± 0.124 | >0.9999 |
|  | *anti-CD47* | Core | 0.866 ± 0.084 | 0.9998 | 0.851 ± 0.204 | 0.9066 |
|  |  | Quiescent | 0.539 ± 0.173 | >0.9999 | 0.718 ± 0.138 | >0.9999 |
|  |  | Edge | 0.097 ± 0.062 | 0.9995 | 0.173 ± 0.146 | 0.9944 |
|  | *anti-PD-L1* | Core | 0.866 ± 0.136 | 0.9997 | 0.871 ± 0.139 | 0.9994 |
|  |  | Quiescent | 0.528 ± 0.183 | 0.9976 | 0.726 ± 0.144 | >0.9999 |
|  |  | Edge | 0.137 ± 0.076 | 0.1258 | 0.192 ± 0.140 | 0.8381 |
|  | *Combination* | Core | 0.864 ± 0.114 | 0.9990 | 0.820 ± 0.225 | 0.3626 |
|  |  | Quiescent | 0.527 ± 0.197 | 0.9966 | 0.684 ± 0.134 | 0.8194 |
|  |  | Edge | 0.107 ± 0.068 | 0.9572 | 0.140 ± 0.152 | 0.9642 |
| 24 Hours | *Control* | Core | 0.849 ± 0.065 | - | 0.891 ± 0.050 | - |
|  |  | Quiescent | 0.598 ± 0.146 | - | 0.594 ± 0.152 | - |
|  |  | Edge | 0.147 ± 0.072 | - | 0.135 ± 0.082 | - |
|  | *5-FU* | Core | 0.870 ± 0.059 | 0.8797 | 0.875 ± 0.128 | 0.9880 |
|  |  | Quiescent | 0.699 ± 0.135 | 0.0786 | 0.743 ± 0.122 | ***0.0003*** |
|  |  | Edge | 0.225 ± 0.109 | ***0.0022*** | 0.217 ± 0.108 | 0.0595 |
|  | *anti-CD47* | Core | 0.798 ± 0.076 | 0.1133 | 0.822 ± 0.117 | 0.2006 |
|  |  | Quiescent | 0.500 ± 0.190 | 0.0924 | 0.521 ± 0.104 | 0.2290 |
|  |  | Edge | 0.154 ± 0.079 | 0.9975 | 0.159 ± 0.125 | 0.9352 |
|  | *anti-PD-L1* | Core | 0.898 ± 0.064 | 0.1581 | 0.897 ± 0.079 | 0.9997 |
|  |  | Quiescent | 0.505 ± 0.157 | 0.1249 | 0.609 ± 0.179 | 0.9935 |
|  |  | Edge | 0.131 ± 0.075 | 0.9435 | 0.132 ± 0.134 | >0.9999 |
|  | *Combination* | Core | 0.868 ± 0.046 | 0.9181 | 0.909 ± 0.035 | 0.9794 |
|  |  | Quiescent | 0.671 ± 0.093 | 0.3477 | 0.697 ± 0.086 | ***0.0314*** |
|  |  | Edge | 0.173 ± 0.079 | 0.7324 | 0.184 ± 0.110 | 0.5013 |
| 48 Hours | *Control* | Core | 0.883 ± 0.049 | - | 0.870 ± 0.094 | - |
|  |  | Quiescent | 0.660 ± 0.122 | - | 0.739 ± 0.150 | - |
|  |  | Edge | 0.149 ± 0.083 | - | 0.247 ± 0.134 | - |
|  | *5-FU* | Core | 0.913 ± 0.031 | 0.6560 | 0.922 ± 0.032 | 0.4864 |
|  |  | Quiescent | 0.723 ± 0.072 | 0.4841 | 0.608 ± 0.098 | ***0.0023*** |
|  |  | Edge | 0.163 ± 0.084 | 0.9629 | 0.082 ± 0.054 | ***<0.0001*** |
|  | *anti-CD47* | Core | 0.793 ± 0.047 | ***0.0003*** | 0.810 ± 0.074 | 0.3220 |
|  |  | Quiescent | 0.473 ± 0.139 | ***<0.0001*** | 0.572 ± 0.178 | ***<0.0001*** |
|  |  | Edge | 0.127 ± 0.102 | 0.8215 | 0.193 ± 0.119 | 0.4023 |
|  | *anti-PD-L1* | Core | 0.866 ± 0.099 | 0.9326 | 0.870 ± 0.089 | >0.9999 |
|  |  | Quiescent | 0.608 ± 0.152 | 0.6804 | 0.634 ± 0.151 | ***0.0267*** |
|  |  | Edge | 0.126 ± 0.085 | 0.7969 | 0.121 ± 0.086 | ***0.0006*** |
|  | *Combination* | Core | 0.886 ± 0.053 | >0.9999 | 0.891 ± 0.039 | 0.9709 |
|  |  | Quiescent | 0.642 ± 0.101 | 0.9918 | 0.588 ± 0.103 | ***0.0002*** |
|  |  | Edge | 0.126 ± 0.098 | 0.8020 | 0.104 ± 0.107 | ***<0.0001*** |


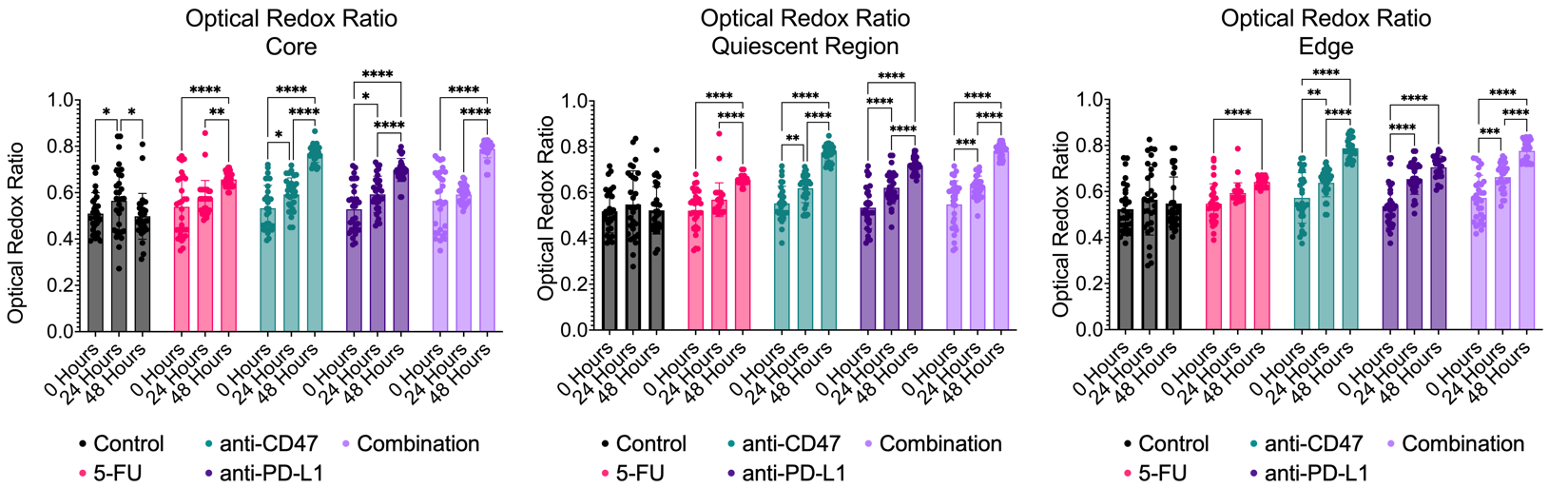


**Supplemental Figure 8. Significant changes were observed across spheroid regions in the metabolic optical redox ratio within each treatment group.** * p ≤ 0.05, ** p ≤ 0.01, *** p ≤ 0.001, **** p ≤ 0.0001. Plots were made in GraphPad Prism ®.

**Supplemental Table 6. Summary of Optical Redox Ratios**

| Timepoint | Group | Region | Optical Redox Ratio ± SD | p-values  (vs Control) |
| --- | --- | --- | --- | --- |
| 0 Hours | *Control* | Core | 0.510 ± 0.090 | - |
|  |  | Quiescent | 0.519 ± 0.094 | - |
|  |  | Edge | 0.523 ± 0.104 | - |
|  | *5-FU* | Core | 0.539 ± 0.129 | 0.7036 |
|  |  | Quiescent | 0.521 ± 0.088 | > 0.9999 |
|  |  | Edge | 0.547 ± 0.086 | 0.8086 |
|  | *anti-CD47* | Core | 0.533 ± 0.098 | 0.8483 |
|  |  | Quiescent | 0.553 ± 0.075 | 0.4486 |
|  |  | Edge | 0.572 ± 0.110 | 0.1587 |
|  | *anti-PD-L1* | Core | 0.528 ± 0.102 | 0.9246 |
|  |  | Quiescent | 0.534 ± 0.091 | 0.9403 |
|  |  | Edge | 0.536 ± 0.095 | 0.9762 |
|  | *Combination* | Core | 0.565 ± 0.129 | 0.1123 |
|  |  | Quiescent | 0.548 ± 0.103 | 0.5863 |
|  |  | Edge | 0.572 ± 0.099 | 0.1619 |
| 24 Hours | *Control* | Core | 0.565 ± 0.143 | - |
|  |  | Quiescent | 0.548 ± 0.147 | - |
|  |  | Edge | 0.564 ± 0.153 | - |
|  | *5-FU* | Core | 0.575 ± 0.078 | 0.9905 |
|  |  | Quiescent | 0.569 ± 0.074 | 0.8505 |
|  |  | Edge | 0.593 ± 0.045 | 0.6664 |
|  | *anti-CD47* | Core | 0.592 ± 0.071 | 0.7430 |
|  |  | Quiescent | 0.619 ± 0.062 | ***0.0056*** |
|  |  | Edge | 0.638 ± 0.061 | ***0.0069*** |
|  | *anti-PD-L1* | Core | 0.593 ± 0.079 | 0.7353 |
|  |  | Quiescent | 0.621 ± 0.070 | ***0.0037*** |
|  |  | Edge | 0.654 ± 0.067 | ***0.0004*** |
|  | *Combination* | Core | 0.592 ± 0.038 | 0.7532 |
|  |  | Quiescent | 0.624 ± 0.048 | ***0.0023*** |
|  |  | Edge | 0.663 ± 0.060 | ***< 0.0001*** |
| 48 Hours | *Control* | Core | 0.498 ± 0.099 | - |
|  |  | Quiescent | 0.523 ± 0.103 | - |
|  |  | Edge | 0.548 ± 0.115 | - |
|  | *5-FU* | Core | 0.657 ± 0.032 | ***< 0.0001*** |
|  |  | Quiescent | 0.656 ± 0.023 | ***< 0.0001*** |
|  |  | Edge | 0.641 ± 0.022 | ***0.0002*** |
|  | *anti-CD47* | Core | 0.769 ± 0.038 | ***< 0.0001*** |
|  |  | Quiescent | 0.775 ± 0.036 | ***< 0.0001*** |
|  |  | Edge | 0.788 ± 0.044 | ***< 0.0001*** |
|  | *anti-PD-L1* | Core | 0.698 ± 0.050 | ***< 0.0001*** |
|  |  | Quiescent | 0.717 ± 0.035 | ***< 0.0001*** |
|  |  | Edge | 0.705 ± 0.049 | ***< 0.0001*** |
|  | *Combination* | Core | 0.788 ± 0.038 | ***< 0.0001*** |
|  |  | Quiescent | 0.781 ± 0.028 | ***< 0.0001*** |
|  |  | Edge | 0.777 ± 0.044 | ***< 0.0001*** |


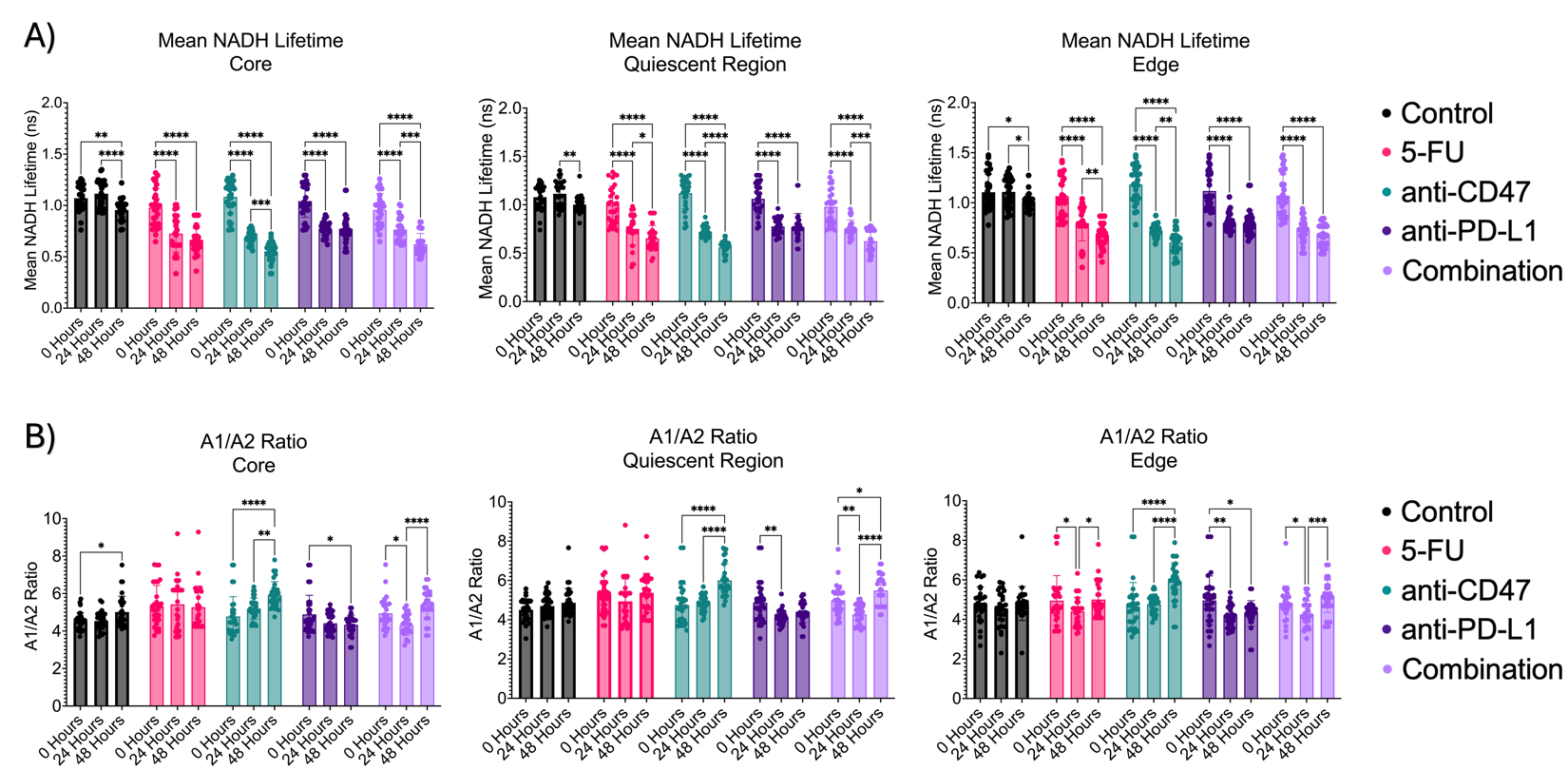


**Supplemental Figure 9. Significant changes were observed across spheroid regions between mean NADH lifetime and A1/A2 ratio within each treatment group.** Top: Mean NADH lifetime values across regions. Bottom: A1/A2 ratios across spheroid regions. * p ≤ 0.05, ** p ≤ 0.01, *** p ≤ 0.001, **** p ≤ 0.0001. Plots were made in GraphPad Prism ®.

**Supplemental Table 7. Summary of Mean NADH Lifetime Values and A1/A2 Ratios**

| Timepoint | Group | Region | Mean NADH Lifetime ± SD | p-values  (vs Control) | A1/A2 Ratio ± SD | p-values  (vs Control) |
| --- | --- | --- | --- | --- | --- | --- |
| 0 Hours | *Control* | Core | 1.071 ± 0.125 | - | 4.501 ± 0.476 | - |
|  |  | Quiescent | 1.079 ± 0.132 | - | 4.507 ± 0.557 | - |
|  |  | Edge | 1.105 ± 0.132 | - | 4.754 ± 0.890 | - |
|  | *5-FU* | Core | 0.978 ± 0.188 | 0.0532 | 5.323 ± 1.110 | 0.1500 |
|  |  | Quiescent | 0.999 ± 0.193 | 0.1452 | 5.287 ± 1.051 | 0.1600 |
|  |  | Edge | 1.067 ± 0.194 | 0.8425 | 4.949 ± 1.266 | 0.9139 |
|  | *anti-CD47* | Core | 1.084 ± 0.160 | 0.9949 | 4.778 ± 1.054 | 0.7039 |
|  |  | Quiescent | 1.120 ± 0.161 | 0.74444 | 4.745 ± 1.063 | 0.7767 |
|  |  | Edge | 1.184 ± 0.179 | 0.2310 | 4.651 ± 1.214 | 0.9917 |
|  | *anti-PD-L1* | Core | 1.043 ± 0.159 | 0.9231 | 4.889 ± 1.019 | 0.3786 |
|  |  | Quiescent | 1.061 ± 0.158 | 0.9867 | 4.867 ± 1.029 | 0.4045 |
|  |  | Edge | 1.118 ± 0.191 | 0.9974 | 4.959 ± 1.244 | 0.8979 |
|  | *Combination* | Core | 0.956 ± 0.161 | 0.0800 | 4.949 ± 0.815 | 0.2342 |
|  |  | Quiescent | 0.980 ± 0.179 | 0.3520 | 4.987 ± 0.785 | 0.1372 |
|  |  | Edge | 1.071 ± 0.210 | 0.8971 | 4.836 ± 0.854 | 0.9965 |
| 24 Hours | *Control* | Core | 1.115 ± 0.130 | - | 4.537 ± 0.469 | - |
|  |  | Quiescent | 1.114 ± 0.137 | - | 4.697 ± 0.552 | - |
|  |  | Edge | 1.109 ± 0.148 | - | 4.541 ± 0.847 | - |
|  | *5-FU* | Core | 0.726 ± 0.181 | ***< 0.0001*** | 5.423 ± 1.336 | ***0.0005*** |
|  |  | Quiescent | 0.750 ± 0.168 | ***< 0.0001*** | 4.938 ± 1.137 | 0.7652 |
|  |  | Edge | 0.797 ± 0.176 | ***< 0.0001*** | 4.406 ± 0.700 | 0.9770 |
|  | *anti-CD47* | Core | 0.691 ± 0.062 | ***< 0.0001*** | 5.195 ± 0.567 | ***0.0210*** |
|  |  | Quiescent | 0.722 ± 0.056 | ***< 0.0001*** | 4.821 ± 0.410 | 0.9740 |
|  |  | Edge | 0.722 ± 0.059 | ***< 0.0001*** | 4.766 ± 0.474 | 0.8622 |
|  | *anti-PD-L1* | Core | 0.771 ± 0.072 | ***< 0.0001*** | 4.411 ± 0.503 | 0.9780 |
|  |  | Quiescent | 0.777 ± 0.082 | ***< 0.0001*** | 4.132 ± 0.360 | ***0.0492*** |
|  |  | Edge | 0.799 ± 0.085 | ***< 0.0001*** | 4.134 ± 0.541 | 0.3890 |
|  | *Combination* | Core | 0.766 ± 0.110 | ***< 0.0001*** | 4.319 ± 0.558 | 0.8528 |
|  |  | Quiescent | 0.753 ± 0.089 | ***< 0.0001*** | 4.277 ± 0.508 | 0.2473 |
|  |  | Edge | 0.720 ± 0.109 | ***< 0.0001*** | 4.258 ± 0.672 | 0.7310 |
| 48 Hours | *Control* | Core | 0.956 ± 0.104 | - | 5.013 ± 0.843 | - |
|  |  | Quiescent | 1.002 ± 0.086 | - | 4.866 ± 0.732 | - |
|  |  | Edge | 1.011 ± 0.076 | - | 4.804 ± 0.857 | - |
|  | *5-FU* | Core | 0.665 ± 0.133 | ***< 0.0001*** | 5.267 ± 1.134 | 0.7668 |
|  |  | Quiescent | 0.653 ± 0.134 | ***< 0.0001*** | 5.372 ± 0.998 | 0.1017 |
|  |  | Edge | 0.671 ± 0.119 | ***< 0.0001*** | 5.002 ± 0.864 | 0.9102 |
|  | *anti-CD47* | Core | 0.552 ± 0.091 | ***< 0.0001*** | 5.922 ± 0.714 | ***0.0003*** |
|  |  | Quiescent | 0.560 ± 0.065 | ***< 0.0001*** | 6.004 ± 0.837 | ***<0.0001*** |
|  |  | Edge | 0.605 ± 0.115 | ***< 0.0001*** | 5.907 ± 0.960 | ***<0.0001*** |
|  | *anti-PD-L1* | Core | 0.773 ± 0.135 | ***< 0.0001*** | 4.334 ± 0.516 | ***0.0155*** |
|  |  | Quiescent | 0.769 ± 0.141 | ***< 0.0001*** | 4.408 ± 0.553 | 0.1714 |
|  |  | Edge | 0.794 ± 0.128 | ***< 0.0001*** | 4.299 ± 0.649 | 0.1806 |
|  | *Combination* | Core | 0.623 ± 0.104 | ***< 0.0001*** | 5.403 ± 0.783 | 0.3745 |
|  |  | Quiescent | 0.624 ± 0.117 | ***< 0.0001*** | 5.492 ± 0.774 | ***0.0208*** |
|  |  | Edge | 0.682 ± 0.107 | ***< 0.0001*** | 5.164 ± 0.818 | 0.5178 |

**Supplemental Table 8. Summary of Pearson Correlations R-values and p-values at Spheroid Core**

|  | CD80 | CD206 | Ki67 | CC3 | HIF-1𝛼 | HIF-2𝛼 | CD47 | SIRP-𝛼 | PD-1 | PD-L1 | Optical Redox Ratio | Mean NADH Lifetime | A1/A2  Ratio |
| --- | --- | --- | --- | --- | --- | --- | --- | --- | --- | --- | --- | --- | --- |
| CD80 |  | **r = 0.61 p<0.0001** | **r = 0.76**  **p<0.0001** | **r = 0.36**  **p<0.0001** | **r = 0.25**  **p<0.0001** | r = -0.07  p=0.1323 | r = -0.09  p=0.0635 | **r = -0.29**  **p<0.0001** | r = 0.05  p=0.2706 | r = 0.02  p=0.6117 | **r = 0.47**  **p<0.0001** | **r = -0.66**  **p<0.0001** | r = 0.05  p=0.2618 |
| CD206 | **r = 0.61**  **p<0.0001** |  | **r = 0.48**  **p<0.0001** | **r = 0.19**  **p<0.0001** | **r = 0.27**  **p<0.0001** | r = 0.03  p=0.5114 | r = -0.07  p=0.1140 | **r = -0.18**  **p=0.0002** | r = 0.05  p=0.2657 | r = -0.04  p=0.4529 | **r = 0.26**  **p<0.0001** | **r = -0.43**  **p<0.0001** | r = 0.08  p=0.1063 |
| Ki67 | **r = 0.76**  **p<0.0001** | **r = 0.48**  **p<0.0001** |  | **r = 0.38**  **p<0.0001** | **r = 0.28**  **p<0.0001** | r = -0.06  p=0.1993 | r = -0.08  p=0.0854 | **r = -0.24**  **p<0.0001** | r = 0.03  p=0.5290 | r = -0.03  p=0.5689 | **r = 0.39**  **p<0.0001** | **r = -0.65**  **p<0.0001** | r = 0.07  p=0.1482 |
| CC3 | **r = 0.36**  **p<0.0001** | **r = 0.19**  **p<0.0001** | **r = 0.38**  **p<0.0001** |  | **r = 0.20**  **p<0.0001** | r = 0.08  p=0.0844 | r = -0.01  p=0.8433 | **r = -0.10**  **p=0.0335** | **r = 0.09**  **p=0.0464** | r = 0.05  p=0.3312 | **r = 0.27**  **p<0.0001** | **r = -0.38**  **p<0.0001** | **r = 0.12**  **p=0.0081** |
| HIF-1𝛼 | **r = 0.25**  **p<0.0001** | **r = 0.27**  **p<0.0001** | **r = 0.38**  **p<0.0001** | **r = 0.20**  **p<0.0001** |  | **r = 0.34**  **p<0.0001** | r = -0.02  p=0.6217 | r = -0.06  p=0.1954 | r = 0.03  p=0.5582 | r = -0.03  p=0.5924 | **r = 0.14**  **p=0.0027** | **r = -0.23**  **p<0.0001** | **r = 0.10**  **p=0.0414** |
| HIF-2𝛼 | r = -0.07  p=0.1323 | r = 0.03  p=0.5114 | r = -0.06  p=0.1993 | r = 0.08  p=0.0844 | **r = 0.34**  **p<0.0001** |  | r = -0.01  p=0.7689 | r = 0.05  p=0.3346 | r = 0.02  p=0.6525 | r = 0.05  p=0.2827 | r = -0.06  p=0.2340 | r = 0.05  p=0.2823 | r = 0.04  p=0.3424 |
| CD47 | r = -0.09  p=0.0635 | r = -0.07  p=0.1140 | r = -0.08  p=0.0854 | r = -0.01  p=0.8433 | r = -0.02  p=0.6217 | r = -0.01  p=0.7689 |  | **r = 0.53**  **p<0.0001** | r = -0.02  p=0.6870 | r = 0.01  p=0.8227 | r = -0.02  p=0.6045 | r = 0.01  p=0.9100 | r = 0.07  p=0.1387 |
| SIRP-𝛼 | **r = -0.29**  **p<0.0001** | **r = -0.18**  **p=0.0002** | **r = -0.24**  **p<0.0001** | **r = -0.10**  **p=0.0335** | r = -0.06  p=0.1954 | r = 0.05  p=0.3346 | **r = 0.53**  **p<0.0001** |  | r = 0.02  p=0.7319 | r = 0.07  p=0.1452 | **r = -0.11**  **p=0.0229** | **r = 0.21**  **p<0.0001** | r = 0.02  p=0.6776 |
| PD-1 | r = 0.05  p=0.2706 | r = 0.05  p=0.2657 | r = 0.03  p=0.5290 | **r = 0.09**  **p=0.0464** | r = 0.03  p=0.5582 | r = 0.02  p=0.6525 | r = -0.02  p=0.6870 | r = 0.02  p=0.7319 |  | **r = 0.35**  **p<0.0001** | r = 0.08  p=0.1054 | r = 0.06  p=0.1968 | **r = -0.12**  **p=0.0132** |
| PD-L1 | r = 0.02  p=0.6117 | r = -0.04  p=0.4529 | r = -0.03  p=0.5689 | r = 0.05  p=0.3312 | r = -0.03  p=0.5924 | r = 0.05  p=0.2827 | r = 0.01  p=0.8227 | r = 0.07  p=0.1452 | **r = 0.35**  **p<0.0001** |  | r = 0.01  p=0.8068 | r = 0.03  p=0.5540 | r = -0.07  p=0.1370 |
| Optical Redox Ratio | **r = 0.47**  **p<0.0001** | **r = 0.26**  **p<0.0001** | **r = 0.39**  **p<0.0001** | **r = 0.27**  **p<0.0001** | **r = 0.14**  **p=0.0027** | r = -0.06  p=0.2340 | r = -0.02  p=0.6045 | **r = -0.11**  **p=0.0229** | r = 0.08  p=0.1054 | r = 0.01  p=0.8068 |  | r = -0.48  **p<0.0001** | **r = 0.10**  **p=0.0375** |
| Mean NADH Lifetime | **r = -0.66**  **p<0.0001** | **r = -0.43**  **p<0.0001** | r = -0.65  **p<0.0001** | **r = -0.38**  **p<0.0001** | **r = -0.23**  **p<0.0001** | r = 0.05  p=0.2823 | r = 0.01  p=0.9100 | **r = 0.21**  **p<0.0001** | r = 0.06  p=0.1968 | r = 0.03  p=0.5540 | **r = -0.48**  **p<0.0001** |  | **r = -0.52**  **p<0.0001** |
| A1/A2  Ratio | r = 0.05  p=0.2618 | r = 0.08  p=0.1063 | r = 0.07  p=0.1482 | r = 0.12  **p=0.0081** | r = 0.10  **p=0.0414** | r = 0.04  p=0.3424 | r = 0.07  p=0.1387 | r = 0.02  p=0.6776 | **r = -0.12**  **p=0.0132** | r = -0.07  p=0.1370 | **r = 0.10**  **p=0.0375** | r = -0.52  **p<0.0001** |  |

Degrees of Correlation: High (r: ± 0.50 – ± 1.0), Moderate (r: ± 0.30 – ± 0.49), Low (r: < ± 0.29); **Bolded values: Statistically significant**

**Supplemental Table 9. Summary of Pearson Correlations R-values and p-values at Spheroid Quiescent Region**

|  | CD80 | CD206 | Ki67 | CC3 | HIF-1𝛼 | HIF-2𝛼 | CD47 | SIRP-𝛼 | PD-1 | PD-L1 | Optical Redox Ratio | Mean NADH Lifetime | A1/A2  Ratio |
| --- | --- | --- | --- | --- | --- | --- | --- | --- | --- | --- | --- | --- | --- |
| CD80 |  | **r = -0.43**  **p=0.0054** | **r = 0.25**  **p=0.0026** | **r = 0.23**  **p=0.0030** | r = 0.07  p=0.6011 | **r = 0.38**  **p<0.0001** | **r = -0.38**  **p=0.0071** | **r = -0.45**  **p=0.0032** | **r = -0.36**  **p=0.0151** | r = 0.06  p=0.1873 | **r = 0.55**  **p<0.0001** | **r = -0.58**  **p<0.0001** | **r = -0.06**  **p=0.0153** |
| CD206 | **r = -0.43**  **p=0.0054** |  | r = -0.17  p=0.9740 | r = -0.14  p=0.7201 | r = -0.14  p=0.5745 | r = -0.37  p=0.1789 | r = -0.04  p=0.6639 | r = 0.13  p=0.0562 | **r = 0.21**  **p=0.0499** | r = -0.12  p=0.7057 | **r = 0.63**  **p<0.0001** | **r = 0.58**  **p<0.0001** | r = -0.27  p=0.3165 |
| Ki67 | **r = 0.25**  **p=0.0026** | r = -0.17  p=0.9740 |  | **r = 0.75**  **p<0.0001** | **r = 0.15**  **p=0.0059** | **r = 0.19**  **p=0.0082** | r = -0.08  p=0.5492 | r = -0.06  p=0.1227 | r = -0.26  p=0.9256 | r = -0.32  p=0.0897 | r = 0.10  p=0.3841 | **r = -0.27**  **p=0.0029** | r = 0.01  p=0.8517 |
| CC3 | **r = 0.23**  **p=0.0030** | r = -0.14  p=0.7201 | **r = 0.75**  **p<0.0001** |  | **r = 0.31**  **p<0.0001** | **r = 0.31**  **p<0.0001** | r = -0.02  p=0.0917 | r = -0.04  p=0.1986 | r =- 0.35  p=0.1467 | r = -0.41  **p=0.0043** | r = 0.01  p=0.3579 | **r = -0.25**  **p=0.0069** | r = 0.05  p=0.5100 |
| HIF-1𝛼 | r = 0.07  p=0.6011 | r = -0.14  p=0.5745 | **r = 0.15**  **p=0.0059** | **r = 0.31**  **p<0.0001** |  | r = 0.77  **p<0.0001** | r = -0.29  p=0.2978 | r = -0.22  p=0.9330 | r = -0.17  p=0.4130 | r = -0.10  p=0.6542 | r = 0.11  p=0.6494 | r = -0.24  p=0.2197 | **r = 0.17**  **p=0.0161** |
| HIF-2𝛼 | **r = 0.38**  **p<0.0001** | r = -0.37  p=0.1789 | **r = 0.19**  **p=0.0082** | **r = 0.31**  **p<0.0001** | **r = 0.77**  **p<0.0001** |  | r = -0.32  p=0.1128 | r = -0.29  p=0.9331 | **r = -0.40**  **p=0.0405** | r = -0.02  p=0.1077 | **r = 0.50**  **p<0.0001** | **r = -0.63**  **p<0.0001** | **r = 0.33**  **p=0.0004** |
| CD47 | **r = -0.38**  **p=0.0071** | r = -0.04  p=0.6639 | r = -0.08  p=0.5492 | r = -0.02  p=0.0917 | r = -0.29  p=0.2978 | r = -0.32  p=0.1128 |  | **r = 0.78**  **p<0.0001** | r = -0.13  p=0.7096 | r = -0.30  p=0.1505 | r = -0.24  p=0.1578 | r = 0.16  p=0.2846 | **r = 0.14**  **p=0.0145** |
| SIRP-𝛼 | **r = -0.45**  **p=0.0032** | r = 0.13  p=0.0562 | r = -0.06  p=0.1227 | r = -0.04  p=0.1986 | r = -0.22  p=0.9330 | r = -0.29  p=0.9331 | **r = 0.78**  **p<0.0001** |  | r = -0.08  p=0.7987 | r = -0.35  p=0.0532 | **r = -0.39**  **p=0.0008** | **r = 0.27**  **p=0.0078** | **r = 0.13**  **p=0.0031** |
| PD-1 | **r = -0.36**  **p=0.0151** | **r = 0.21**  **p=0.0499** | r = -0.26  p=0.9256 | r = -0.35  p=0.1467 | r = -0.17  p=0.4130 | **r = -0.40**  **p=0.0405** | r = -0.13  r=0.7096 | r = -0.08  p=0.7987 |  | **r = 0.54**  **p<0.0001** | **r = -0.46**  **p<0.0001** | **r = 0.48**  **p<0.0001** | **r = -0.36**  **p=0.0381** |
| PD-L1 | r = 0.05  p=0.1873 | r = -0.12  p=0.7057 | r = -0.32  p=0.0897 | **r = -0.41**  **p=0.0043** | r = -0.10  p=0.6542 | r = -0.02  p=0.1077 | r = -0.30  p=0.1505 | r = -0.35  p=0.0532 | **r = 0.54**  **p<0.0001** |  | r = 0.08  p=0.2048 | r = -0.07  p=0.0636 | r = -0.16  p=0.2508 |
| Optical Redox Ratio | **r = 0.55**  **p<0.0001** | **r = -0.63**  **p<0.0001** | r = 0.10  p=0.3841 | r = 0.01  p=0.3579 | r = 0.11  p=0.6494 | **r = 0.50**  **p<0.0001** | r = -0.24  p=0.1578 | **r = -0.39**  **p=0.0008** | **r = -0.46**  **p<0.0001** | r = 0.08  p=0.2048 |  | **r = -0.84**  **p<0.0001** | **r = 0.37**  **p=0.0025** |
| Mean NADH Lifetime | **r = -0.58**  **p<0.0001** | **r = 0.58**  **p<0.0001** | **r = -0.27**  **p=0.0029** | **r = -0.25**  **p=0.0069** | r = -0.24  p=0.2197 | **r = -0.63**  **p<0.0001** | r = 0.16  p=0.2846 | **r = 0.27**  **p=0.0078** | **r = 0.48**  **p<0.0001** | r = -0.07  r=0.0636 | **r = -0.84**  **p<0.0001** |  | **r = -0.66**  **p<0.0001** |
| A1/A2  Ratio | **r = -0.06**  **p=0.0153** | r = -0.27  p=0.3165 | r = 0.01  p=0.8517 | r = 0.05  p=0.5100 | **r = 0.17**  **p=0.0161** | **r = 0.33**  **p=0.0004** | **r = 0.14**  **p=0.0145** | **r = 0.13**  **p=0.0031** | **r = -0.36**  **p=0.0381** | r = -0.16  p=0.2508 | **r = 0.37**  **p=0.0025** | **r = -0.66**  **p<0.0001** |  |

Degrees of Correlation: High (r: ± 0.50 – ± 1.0), Moderate (r: ± 0.30 – ± 0.49), Low (r: < ± 0.29); **Bolded values: Statistically significant**

**Supplemental Table 10. Summary of Pearson Correlations p-values at Spheroid Edge**

|  | CD80 | CD206 | Ki67 | CC3 | HIF-1𝛼 | HIF-2𝛼 | CD47 | SIRP-𝛼 | PD-1 | PD-L1 | Optical Redox Ratio | Mean NADH Lifetime | A1/A2  Ratio |
| --- | --- | --- | --- | --- | --- | --- | --- | --- | --- | --- | --- | --- | --- |
| CD80 |  | r = 0.84  **p<0.0001** | **r = 0.72**  **p<0.0001** | **r = 0.44**  **p<0.0001** | **r = 0.53**  **p<0.0001** | **r = -0.24**  **p<0.0001** | r = -0.02  p=0.5922 | r = 0.03  p=0.5584 | **r = 0.13**  **p=0.0063** | **r = -0.16**  **p=0.0010** | **r = -0.54**  **p<0.0001** | r = 0.70  **p<0.0001** | r = -0.002  p=0.8532 |
| CD206 | **r = 0.84**  **p<0.0001** |  | **r = 0.67**  **p<0.0001** | **r = 0.36**  **p<0.0001** | **r = 0.46**  **p<0.0001** | **r = -0.26**  **p<0.0001** | r = 0.002  p=0.9189 | r = 0.02  p=0.6545 | r = 0.07  p=0.1251 | **r = -0.17**  **p=0.0004** | **r = -0.45**  **p<0.0001** | **r = 0.64**  **p<0.0001** | r = -0.02  p=0.5303 |
| Ki67 | **r = 0.72**  **p<0.0001** | r = 0.67  **p<0.0001** |  | **r = 0.08**  **p<0.0001** | **r = 0.53**  **p<0.0001** | **r = -0.16**  **p=0.0007** | r = -0.01  p=0.7496 | r = 0.06  p=0.2727 | r = 0.09  p=0.0511 | **r = -0.19**  **p<0.0001** | **r = -0.38**  **p<0.0001** | **r = 0.61**  **p<0.0001** | r = 0.03  p=0.6492 |
| CC3 | **r = 0.44**  **p<0.0001** | **r = 0.36**  **p<0.0001** | **r = 0.52**  **p<0.0001** |  | **r = 0.37**  **p<0.0001** | r = -0.05  p=0.2707 | r = 0.02  p=0.8427 | r = 0.05  p=0.3645 | r = 0.03  p=0.4985 | **r = -0.15**  **p=0.0019** | **r = -0.29**  **p<0.0001** | **r = 0.43**  **p<0.0001** | r = -0.04  p=0.3826 |
| HIF-1𝛼 | **r = 0.53**  **p<0.0001** | **r = 0.46**  **p<0.0001** | **r = 0.53**  **p<0.0001** | **r = 0.37**  **p<0.0001** |  | **r = 0.16**  **p=0.0008** | r = -0.02  p=0.8246 | r = 0.02  p=0.7054 | r = 0.07  p=0.1372 | **r = -0.13**  **p=0.0059** | **r = -0.34**  **p<0.0001** | **r = 0.42**  **p<0.0001** | **r = 0.10**  **p=0.0384** |
| HIF-2𝛼 | **r = -0.24**  **p<0.0001** | **r = 0.26**  **p<0.0001** | r = -0.16  **p=0.0007** | r = -0.05  p=0.2707 | **r = 0.16**  **p=0.0008** |  | r = -0.01  p=0.8320 | r = 0.05  p=0.3139 | r = -0.003  p=0.9785 | r = 0.03  p=0.5130 | **r = 0.18**  **p=0.0001** | **r = -0.27**  **p<0.0001** | **r = 0.20**  **p<0.0001** |
| CD47 | r = -0.02  p=0.5922 | r = 0.002  p=0.9189 | r = -0.01  p=0.7496 | r = 0.02  p=0.8427 | r = -0.02  p=0.8246 | r = -0.01  p=0.8320 |  | **r = 0.40**  **p<0.0001** | r = 0.04  p=0.5679 | r = -0.005  p=0.9438 | r = -0.001  p=0.8971 | r = -0.01  p=0.5983 | **r = 0.13**  **p=0.0022** |
| SIRP-𝛼 | r = 0.03  p=0.5584 | r = 0.02  p=0.6545 | r = 0.06  p=0.2727 | r = 0.05  0.3645 | r = 0.02  p=0.7054 | r = 0.05  p=0.3139 | **r = 0.40**  **p<0.0001** |  | r = 0.05  p=0.3356 | r = 0.04  p=0.5159 | r = -0.01  p=0.9268 | r = -0.01  p=0.7847 | **r = 0.15**  **p=0.0007** |
| PD-1 | **r = 0.13**  **p=0.0063** | r = 0.07  p=0.1251 | r = 0.09  p=0.0511 | r = 0.03  p=0.4985 | r = 0.07  p=0.1372 | r = -0.003  p=0.9785 | r = 0.03  p=0.5679 | r = 0.05  p=0.3356 |  | **r = 0.40**  **p<0.0001** | r = -0.07  p=0.1243 | r = 0.08  p=0.0627 | r = -0.01  p=0.5599 |
| PD-L1 | **r = -0.16**  **p=0.0010** | r = -0.17  **p=0.0004** | **r = -0.19**  **p<0.0001** | **r = -0.15**  **p=0.0019** | **r = -0.13**  **p=0.0059** | r = 0.03  p=0.5130 | r = -0.005  p=0.9438 | r = 0.04  p=0.5159 | **r = 0.40**  **p<0.0001** |  | r = 0.01  p=0.8713 | **r = -0.14**  **p=0.0070** | r = -0.03  p=0.4156 |
| Optical Redox Ratio | **r = -0.54**  **p<0.0001** | **r = -0.45**  **p<0.0001** | **r = -0.38**  **p<0.0001** | **r = -0.29**  **p<0.0001** | **r = -0.34**  **p<0.0001** | **r = 0.18**  **p=0.0001** | r =- 0.001  p=0.8971 | r = -0.01  p=0.9268 | r = -0.07  p=0.1243 | r = 0.01  p=0.8713 |  | **r = -0.42**  **p<0.0001** | r = 0.02  p=0.4691 |
| Mean NADH Lifetime | **r = 0.70**  **p<0.0001** | **r = 0.64**  **p<0.0001** | **r = 0.61**  **p<0.0001** | **r = 0.43**  **p<0.0001** | r = 0.42  **p<0.0001** | r = -0.27  **p<0.0001** | r = -0.01  p=0.5983 | r = -0.01  p=0.7847 | r = 0.08  p=0.0627 | **r = -0.14**  **p=0.0070** | **r = -0.42**  **p<0.0001** |  | **r = -0.32**  **p<0.0001** |
| A1/A2  Ratio | r = -0.002  p=0.8532 | r = -0.02  p=0.5303 | r = 0.03  p=0.6492 | r = -0.04  p=0.3826 | **r = 0.10**  **p=0.0384** | **r = 0.20**  **p<0.0001** | **r = 0.13**  **p=0.0022** | r = 0.15  **p=0.0007** | r = -0.01  p=0.5599 | r = -0.03  p=0.4156 | r = 0.02  p=0.4691 | **r = -0.32**  **p<0.0001** |  |

Degrees of Correlation: High (r: ± 0.50 – ± 1.0), Moderate (r: ± 0.30 – ± 0.49), Low (r: < ± 0.29); **Bolded values: Statistically significant**

**Supplemental Table 11. Summary of Pearson Correlations R-values and p-values of 2D Co-Culture Model**

|  | CD80 | CD206 | Nuclear HMGB1 | Cyto. HMGB1 | Calreticulin | CD47 | SIRP-𝛼 | PD-L1 | % Phago. | P. Index | P. Capacity |
| --- | --- | --- | --- | --- | --- | --- | --- | --- | --- | --- | --- |
| CD80 |  | r = -0.307  p = 0.388 | r = -0.529  p = 0.116 | r = 0.529  p = 0.116 | **r = -0.692**  **p = 0.027** | **r = -0.725**  **p = 0.018** | **r = 0.784**  **p = 0.0073** | **r = 0.730**  **p = 0.017** | r = 0.248  p = 0.490 | r = 0.405  p = 0.236 | r = 0.604  p = 0.065 |
| CD206 | r = -0.307  p = 0.388 |  | r = -0.055  p = 0.881 | r = 0.055  p = 0.881 | r = -0.219  p = 0.544 | r = 0.455  p = 0.187 | r = -0.519  p = 0.1244 | r = -0.134  p = 0.711 | r = -0.076  p = 0.834 | r = -0.321  p = 0.367 | r = -0.412  p = 0.236 |
| Nuclear HMGB1 | r = -0.529  p = 0.116 | r = -0.055  p =0.881 |  |  | r = 0.579  p = 0.080 | **r = 0.655**  **p = 0.040** | r = -0.620  p = 0.0558 | r = -0.301  p = 0.397 | r = 0.206  p = 0.569 | r = 0.240  p = 0.505 | r = -0.402  p = 0.249 |
| Cyto. HMGB1 | r = 0.529  p = 0.116 | r = 0.055  p =0.881 |  |  | r = -0.579  p = 0.080 | **r = -0.655**  **p = 0.040** | r = 0.620  p = 0.0558 | r = 0.301  p = 0.397 | r = -0.206  p = 0.569 | r = -0.240  p = 0.505 | r = 0.402  p = 0.249 |
| Calreticulin | **r = -0.692**  **p = 0.0028** | r = -0.219  p =0.544 | r = 0.579  p = 0.080 | r = -0.579  p = 0.080 |  | r = 0.419  p = 0.228 | r = -0.624  p = 0.0536 | **r = -0.830**  **p = 0.003** | r = -0.458  p = 0.183 | r = -0.362  p = 0.303 | r = -0.034  p = 0.925 |
| CD47 | **r = -0.725**  **p = 0.018** | r = 0.455  p =0.187 | **r = 0.655**  **p = 0.040** | **r = -0.655**  **p = 0.040** | r = 0.419  p = 0.228 |  | **r = -0.908**  **p = 0.0003** | r = -0.291  p = 0.414 | r = 0.141  p = 0.698 | r = -0.180  p = 0.767 | **r = -0.809**  **p = 0.005** |
| SIRP-𝛼 | **r = 0.784**  **p = 0.007** | r = -0.519  p = 0.124 | r = -0.620  p = 0.056 | r = 0.620  p = 0.056 | r = -0.624  p = 0.054 | **r = -0.908**  **p < 0.0001** |  | r = 0.597  p = 0.068 | r = 0.197  p = 0.585 | r = 0.364  p = 0.301 | r = 0.547  p = 0.1020 |
| PD-L1 | **r = 0.730**  **p = 0.0017** | r = -0.134  p =0.711 | r = -0.301  p = 0.397 | r = 0.301  p = 0.397 | **r = -0.830**  **p = 0.003** | r = -0.291  p = 0.414 | r = 0.597  p = 0.0684 |  | **r = 0.761**  **p = 0.011** | **r = 0.719**  **p = 0.019** | r = -0.042  p = 0.909 |
| % Phago. | r = 0.248  p = 0.490 | r = -0.076  p = 0.834 | r = 0.206  p = 0.569 | r = -0.206  p = 0.569 | r = -0.458  p = 0.183 | r = 0.141  p = 0.698 | r = 0.197  p = 0.5852 | **r = 0.761**  **p = 0.011** |  | **r = 0.918**  **p < 0.0001** | r = -0.443  p = 0.199 |
| P. Index | r = 0.405  p = 0.246 | r = -0.321  p = 0.367 | r = 0.240  p = 0.505 | r = -0.240  p = 0.505 | r = -0.362  p = 0.303 | r = -0.108  p = 0.767 | r = 0.364  p = 0.3013 | **r = 0.719**  **p = 0.019** | **r = 0.918**  **p < 0.0001** |  |  |
| P. Capacity | r = 0.604  p = 0.065 | r = -0.412  p = 0.236 | r = -0.402  p = 0.249 | r = 0.402  p = 0.249 | r = -0.034  p = 0.925 | **r = -0.809**  **p = 0.005** | r = 0.547  p = 0.1020 | r = -0.042  p = 0.909 | r = -0.443  p = 0.199 | r = -0.132  p = 0.716 |  |

Degrees of Correlation: High (r: ± 0.50 – ± 1.0), Moderate (r: ± 0.30 – ± 0.49), Low (r: < ± 0.29); **Bolded values: Statistically significant**
